# A N^7^-Methylguanine-Related Gene Signature Applicable for the Prognosis and Microenvironment of Prostate Cancer

**DOI:** 10.1155/2022/8604216

**Published:** 2022-05-13

**Authors:** Wangli Mei, Xuyang Jia, Shiyong Xin, Xiang Liu, Liang Jin, Xianchao Sun, Jia-Xin Zhang, Bihui Zhang, Guosheng Yang, Ping Chen, Lin Ye

**Affiliations:** ^1^Department of Urology, Shanghai East Hospital, School of Medicine, Tongji University, Shanghai 200120, China; ^2^Department of Urology, Shanghai Tenth People's Hospital, School of Medicine, Tongji University, Shanghai 200072, China; ^3^Department of Metabolic Surgery, Shanghai Tenth People's Hospital, School of Medicine, Tongji University, Shanghai 200072, China; ^4^Department of Urology, Shanghai Putuo District People's Hospital, School of Medicine, Tongji University, Shanghai 200060, China; ^5^School of Life Science and Technology, Tongji University, Shanghai 200092, China

## Abstract

**Background:**

Despite the constant iteration of small-molecule inhibitors and immune checkpoint inhibitors, PRAD (prostate adenocarcinoma) patients with distant metastases and biochemical recurrence maintain a poor survival outcome along with an increasing morbidity in recent years. N^7^-Methylguanine, a new-found type of RNA modification, has demonstrated an essential role in tumor progression but has hardly been studied for its effect on prostate carcinoma. The current study aimed to seek m^7^G (N7-methylguanosine) related prognostic biomarkers and potential targets for PRAD treatment.

**Methods:**

42 genes related to m^7^G were collected from former literatures and GSEA (Gene Set Enrichment Analysis) website. Then, RNA-seq (RNA sequencing) and clinical data from TCGA-PRAD (The Cancer Genome Atlas-Prostate) cohort were retrieved to screen the differentially expressed m^7^G genes to further construct a multivariate Cox prognostic model for PRAD. Next, GSE116918, a prostate cancer cohort acquired from GEO (Gene Expression Omnibus) database, was analyzed for the external validation group to assess the ability to predict BFFS (biochemical failure-free survival) of our m^7^G prognostic signature. Kaplan-Meier, ROC (receiver operator characteristic), AUC (areas under ROC curve), and calibration curves were adopted to display the performance of this prognostic signature. In addition, immune infiltration analysis was implemented to evaluate the effect of these m^7^G genes on immunoinfiltrating cells. Correlation with drug susceptibility of the m^7^G signature was also analyzed by matching drug information in CellMiner database.

**Results:**

The m^7^G-related prognostic signature, including three genes (EIF3D, EIF4A1, LARP1) illustrated superior prognostic ability for PRAD in both training and validation cohorts. The 5-year AUC were 0.768 for TCGA-PRAD and 0.608 for GSE116918. It can well distinguish patients into different risk groups of biochemical recurrence (*p* =1e-04 for TCGA-PRAD and *p* =0.0186 for GSE116918). Immune infiltration analysis suggested potential regulation of m^7^G genes on neutrophils and dendritic cells in PRAD.

**Conclusions:**

A m^7^G-related prognostic signature was constructed and validated in the current study, giving new sights of m^7^G methylation in predicting the prognostic and improving the treatment of PRAD.

## 1. Introduction

Reportedly, the morbidity of PRAD in men has grown to the second highest, being the sixth leading cause of death for men [1]. About 93% PRAD patients were discovered in the local stages whose five-year OS (overall survival) rate near to 100% [2]. In contrast, the OS was less than 30% in patients accompanied with distant metastases [3]. At present, PSA (prostate-specific antigen) is the best first-step serum marker for the screening of prostate tumor, and it remains the most commonly used tumor marker [4]. Although PSA has achieved remarkable results in the early detection of prostate cancer, there is no consensus on whether PSA can effectively reduce the death risk for PRAD patients [5]. Currently, the treatment for PRAD, especially for the advanced PRAD, was still conventional radiotherapy and chemotherapy [6]. But for patients with advanced PRAD, the comprehensive efficacy was still not ideal. Therefore, what is urgently necessary is to find novel biomarkers for the diagnosis, prognosis, and treatment of PRAD to improve the survival outcome of patients in terminal stage.

It is known that m^7^G, one type of RNA modification, is significantly associated with various biological processes [7] and is widely found in eukaryotes and prokaryotes [8]. It is widely distributed in the 5′ cap region of tRNA [9], rRNA [10], and eukaryotic mRNA [11] to modulate RNA processing [12, 13], elongation [14], splicing [15], nucleation, and protein translation. M^7^G has been reported related with the occurrence of primordial dwarfism in human [8]. The level of m^7^G was significantly reduced in the primordial dwarfism patients with a missense mutation in gene encoding WDR4 [16]. Additionally, the m^7^G level was also related with tumor cell chemoresistance [16], cell cycle, and oncogenicity [17]. Decreased m^7^G tRNA modification could induce oncogenicity of lung cancer by decreasing cell proliferation, colony formation, and cell invasion [18]. Although m^7^G has had certain research results, the roles of the m^7^G-related genes and corresponding RNA modification process in PRAD remain unknown. In addition, it is unclear for the prognostic power of m^7^G-related genes in PRAD. The current study aimed to seek m^7^G-related prognostic biomarkers for terminal-stage PRAD patients, providing potential drug targets for advanced prostate cancer patients' treatment.

## 2. Material and Methods

### 2.1. Differentially Expressed N7-Methyladenosine-Related Genes

The RNA-seq data of 551 samples, including 52 normal samples and 499 PRAD samples, was downloaded in TCGA database [19] in the form of row counts. The clinical data, such as age, sex, tumor grade, Gleason score, and PSA, was also acquired from TCGA. Similar information about the external validation cohort (GSE116918) was acquired from GEO database. The m^7^G-related gene sets, “GOMF M^7^G 5 PPPN DIPHOSPHATASE ACTIVITY,” “GOMF RNA 7 METHYLGUANOSINE CAP BINDING,” and “GOMF RNA CAP BINDING” contain 26 genes which were related with m^7^G, were downloaded from the GSEA [20]. Even more, according to previous reports, we finally found other 16 m^7^G-related genes which were illustrated in supplementary materials (available here). The “limma” [21], a kind of R packages, was used to recognize m^7^G-related differentially expressed genes (mDEGs), with *p* < 0.05. To explore the potential regulating relationship, STRING (Search Tool for the Retrieval of Interacting Genes) [22] was employed to seek possible regulating relationship among these mDEGs in version 11.5.

### 2.2. Analysis and Validation of Our Prognostic Signature

These mDEGs were subsequently subjected to estimate their prognostic power by univariate Cox regression with *p* < 0.05. Then, lasso (least absolute shrinkage and selection operator) [23] Cox regression analysis was adopted for this prognostic signature construction by using R package “glmnet.” Finally, three mDEGs were included in the prognostic signature after lasso penalty. The proportional hazard was counted as the formula: risk score = ∑_*i*_^3^*Xi*∗*Yi* (*X*: coefficients, *Y*: gene expression). The forest plot was drawn by “survminer” package to show hazard ratio and confidence interval of genes. Patients were stratified into low- and high-risk clusters based on optimal cut-off; their survival difference was displayed in KM (Kaplan-Meier) curves. PCA (principal component analysis) was conducted to demonstrate the distance of subgroups by using R package stats. R packages, including “survminer” and “timeROC,” were also employed to compute ROC curves of 1, 3, 5 years for the prognostic signature. Same analytic strategy was also conducted in the external validation cohort GSE116918.

### 2.3. The Prognostic Value of This m^7^G-Related Signature

Univariate and multivariate Cox regression analyses were employed to assess prognostic value for this signature. HR of this signature and traditional clinical factors for prognostic, including Gleason score and tumor grade, were displayed in the forest map and heat map. Same strategy was also carried out in the external validation cohort GSE116918. According to multivariate Cox regression analysis' results, a nomogram combing the m^7^G-related signature and clinical factors was depicted to provide easy access to patients' prognostic risk.

The calibration curve was also demonstrated to verify the integrity of the above nomogram.

### 2.4. The Expression Validation of 3 Genes in m^7^G Signature

To understand the expression of these genes in PRAD patients with different stages, the clinical correlation analysis was utilized between PRAD clinical characteristics and the level of risk score, as well as expression level of these genes. In addition, immunohistochemical images of EIF4A1, EIF3D, and LARP1 in normal tissues and PRAD tissues for this study were downloaded from the Human Protein Atlas [24], which were used for further understanding their expression situation in proteins.

### 2.5. Downstream Regulatory Pathways and Infiltrating Immune Cells of the m^7^G-Related Signature

To determine the downstream regulatory pathway of genes in the m^7^G-related signature, differential analysis was accomplished between different risk subsets where DEGs were selected when its |log2FC| ≥ 1 and FDR < 0.05.

Then, KEGG (Kyoto Encyclopedia of Genes and Genomes) and GO (Gene Ontology) analyses were conducted with R package “clusterProfiler” [25] for these DEGs. GSVA (Gene Set Variation Analysis) [26] was utilized to analyze the relationship of some immune cells and immune pathways by R package “GSVA.” Furthermore, correlation analysis was performed between immune cells and immune pathways to computing their potential regulating relationship.

### 2.6. Drug Sensitivity Analysis for the 3 Genes in m^7^G Signature

In the data of the US NCI (National Cancer Institute) 60, a total of 60 cancer cell lines have been derived from nine different cancers [27], which was acquired from CellMiner database. Pearson's correlation analysis was utilized to assess the relationship between drug response and gene expression levels related to m^7^G.

### 2.7. Statistic and Software

The data was processed and analyzed using R 4.41 (package: limma, ggplot2, survminer, timeROC, GSVA, and so on). Statistical correlations of parametric and nonparametric variables were analyzed using the Pearson and Spearman correlations. We considered all data analyses to be statistically significant with *p* < 0.05.

## 3. Results

### 3.1. Differential Analysis between Normal and Tumor Prostate Tissues

**Figure 1:**
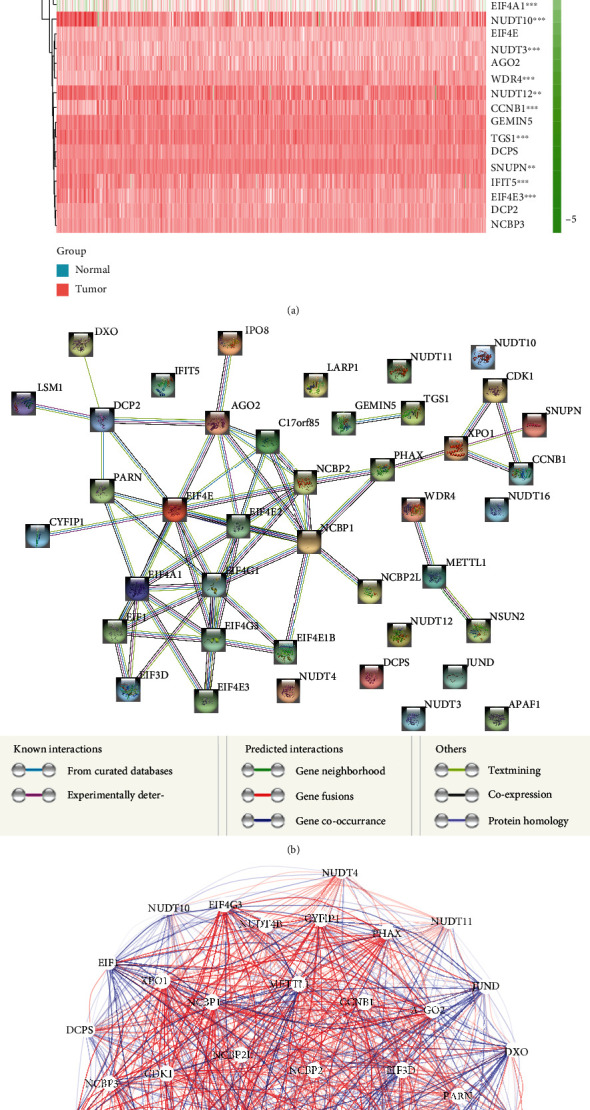
Expression of 42 m^7^G-related genes and their interactions. (a) An overview of the genes on this heat map (∗∗*P* < 0.01; ∗∗∗*P* < 0.001). (b) Network illustrating gene interactions among m^7^G-related genes. (c) A correlation network showing relationship between genes related with m^7^G (positive correlation with red line; negative correlation with blue line).

Differential analysis of the 42 m^7^G-related genes was conducted between PRAD tissues and normal prostate tissues in TCGA, and 16 DEGs were identified with *p* < 0.05. As illustrated in Figure 1(a), a heat map showed the expression levels for these mRNAs related to m^7^G. To discover the possible interactions between m^7^G-related genes, protein-protein interactions analysis was conducted (Figure 1(b)). For the interaction analysis, a minimum interaction score of 0.9 was required, and the result shows that NCBP1, NCBP2, AGO2, EIF4A1, EIF43D, EIF4G3, and EIF4E2 were hub genes. The correlation network which contained 42 m^7^G-related genes is demonstrated in Figure 1(c).

### 3.2. A m^7^G-Related Gene Prognostic Model in TCGA

**Figure 2:**
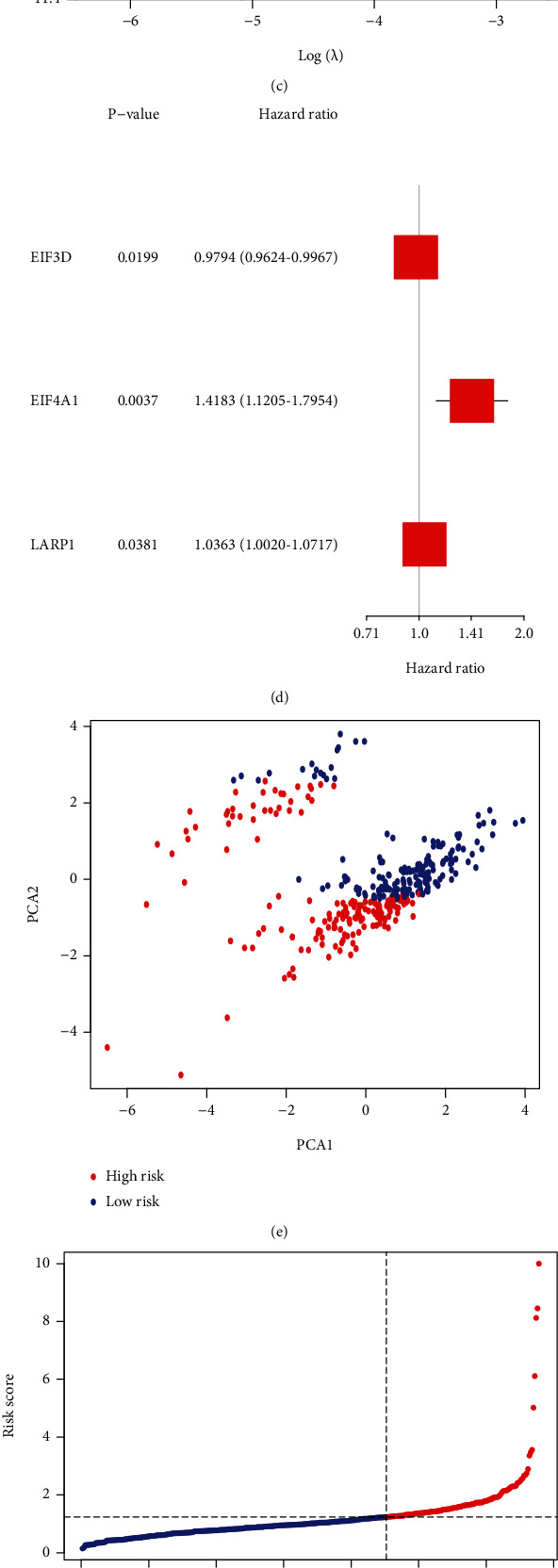
Signature of risk in TCGA. (a) Results of univariate Cox regression analysis for 16 mDEGs. (b) Lasso regression analysis of 3 genes. (c) Lasso regression analysis with cross-validation. (d) Multivariable Cox regression of the 3 genes. (e) PCA plot for PRAD patients. (f) Distributions of PRAD patients based on this signature. (g) Distributions of BFFS status and risk score. (h) KM curves for BFFS. (i) ROC curves of risk score.

After removing the sample with missing clinical data, the 339 samples were used for further analysis. Firstly, the primary screening of genes associated with BCR (biochemical recurrence) was estimated by univariate Cox regression analysis. To keep prognosis value of our prognostic signature, the cut-off *p*-value for the study was set at 0.05, and 3 genes (EIF3D, EIF4A1, LARP1) were obtained (Figure 2(a)). Then, the lasso Cox regression analysis was carried out for further screening, then a 3-gene signature was acquired with the ideal *λ* value (Figures 2(b) and 2(c)). The multivariable Cox regression was employed in the 3 genes, and the result shows that EIF3D (*p* = 0.020), EIF4A1 (*p* = 0.004), LARP1 (*p* = 0.038) could be well prognostic genes (Figure 2(d)). Based on the following calculation, risk score was computed: risk score = (−0.021∗EIF3D exp.) + (0.349∗EIF4A1 exp.) + (0.036∗LARP1 exp.). In accordance with the optimum standard, 339 patients were separated: 226 patients with low risk and 113 patients with high risk. Then, the result of PCA illustrated that PRAD patients from TCGA with varying risk scores were well separated into two different groups (Figure 2(e)). A higher number of BCR samples were collected from patients with higher risk scores; meanwhile, a shorter BFFS time was recorded than that with lower risk scores (Figures 2(f) and 2(g)). With *p* < 0.001, significant differences were found between the BFFS times of the two groups (Figure 2(h)). The sensitivity and specificity of this m^7^G gene prognostic signature were evaluated by ROC analysis. The results of ROC illustrated that AUC was about 0.768 for 1 year, 0.666 for 3 years, and 0.681 for 5 years BCR (Figure 2(i)).

### 3.3. External Validation of This Prognostic Signature

**Figure 3:**
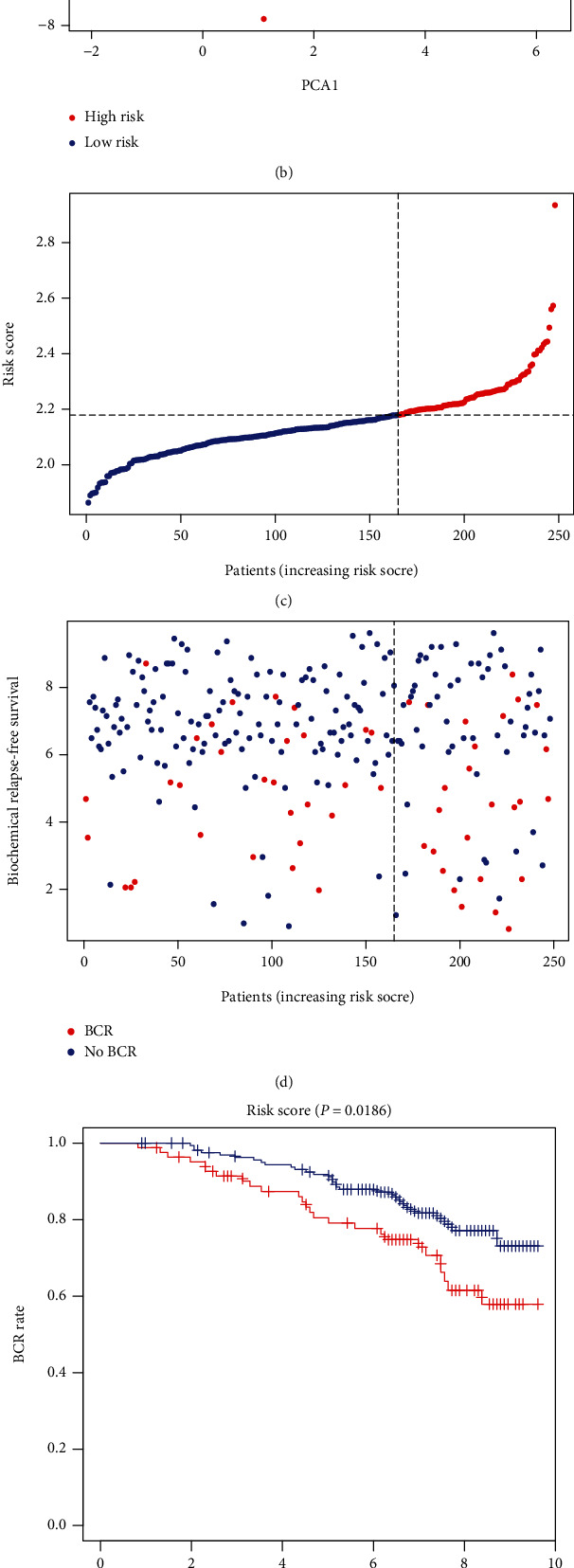
Validation of this signature in GEO. (a) Multivariable Cox regression of 3 genes in the signature. (b) PCA plot for prostate cancer patients. (c) PRAD patient distribution according to signature. (d) BFFS status and BFFS risk score distributions. (e) KM curves for the BFFS. (f) ROC curves of the signature.

The data of extra RNA-seq and clinical characteristics of 248 PRAD patients were downloaded in a GEO cohort (GSE116918) for external validation. The multivariable Cox regression of 3 genes was also conducted in GEO cohort, and the result shows EIF3D (*p* = 0.896), EIF4A1 (*p* = 0.031), LARP1 (*p* = 0.014) (Figure 3(a)). The GEO cohort was reclassified as either low or high risk based on the sores in the TCGA cohort, with 165 patients classified as low risk, and the other 83 patients as high risk. The PCA analysis illustrated significant separation of the two different risk groups (Figures 3(b) and 3(c)). Patients with lower risk scores were found to have longer BFFS time and lower BCR rate (Figure 3(d)). According to the external validation model, BFFS time also significantly associated with risk score with *p* = 0.019 (Figure 3(e)). In GEO cohort, ROC curve indicated that our prognostic signature had significant prognostic value with AUC of 0.920 for 1 year, 0.588 for 3 years, and 0.610 for 5 years depending on the BCR (Figure 3(f)).

### 3.4. Evaluation of Prognostic Value of m^7^G-Related Gene Signature

**Figure 4:**
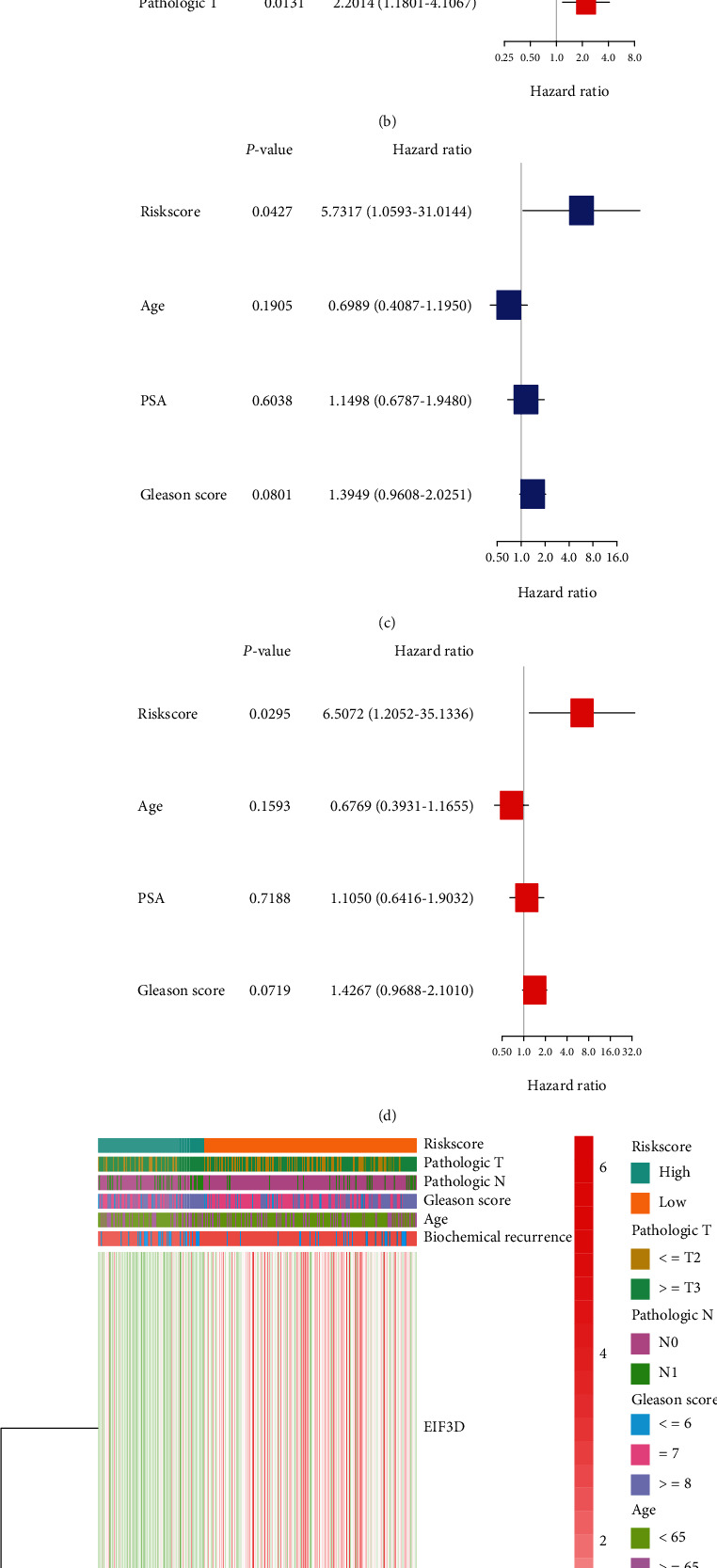
Analyses of univariate and multivariate Cox regression. (a and b) TCGA analysis using univariate and multivariate cox analysis. (c and d) Cox analysis of GEO cohort using univariate and multivariate methods. (e) The association of clinical features with risk groups shown in the heat map (low expression in green; high expression in red).

**Figure 5:**
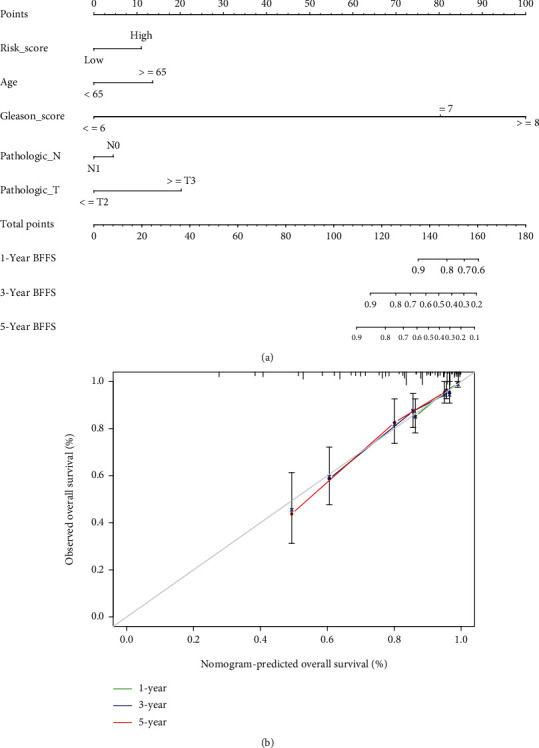
An example of a signature nomogram. (a) Prediction of 1-, 3-, and 5-year BFFS using a nomogram-based genes signature. (b) A calibration plot depicting the agreement between the BFFS prediction and the actual observation over 1, 3, and 5 years.

Table 1 summarizes clinical features of PRAD patients (339 patients from TCGA and 248 from CEO cohorts). Univariate and multivariate Cox regression analyses were employed in order to assess whether this prognostic signature could well predict the BFFS independently. As determined by outcomes of univariate Cox regression, the signature could be a predictor of survival of PRAD patients in TCGA (*p* < 0.001) and GEO (*p* = 0.043) database (Figures 4(a) and 4(c)). Using the multivariable Cox regression analysis, we found this risk signature could be a well independent predictor of BCR for PRAD patients, independently of age, gender, and other confounding factors in TCGA (*p* < 0.001, Figure 4(b)) and GEO (*p* = 0.030, Figure 4(d)) cohorts. Based on clusters divided by the risk scores, we created a heat map to illustrate the differences in the distribution of clinical characteristics between different risk groups' patients in TCGA (Figure 4(e)).  Figure 5(a) shows the situation that the prognostic nomogram for BFFS from the TCGA cohort included all significant clinical findings. In addition, the calibration curve for the probability of recurrence of biochemical abnormalities in PRAD patients at 1, 3, or 5 years showed optimal agreement with predictions derived by the nomogram (Figure 5(b)). On the blue curve, the prediction model accuracy for survival over one year was represented. Likewise, the violet curve represented survival over three years and the red curve represented survival over five years.

### 3.5. Expression Validation of the Risk Model in Prostate Cancer Patient

**Figure 6:**
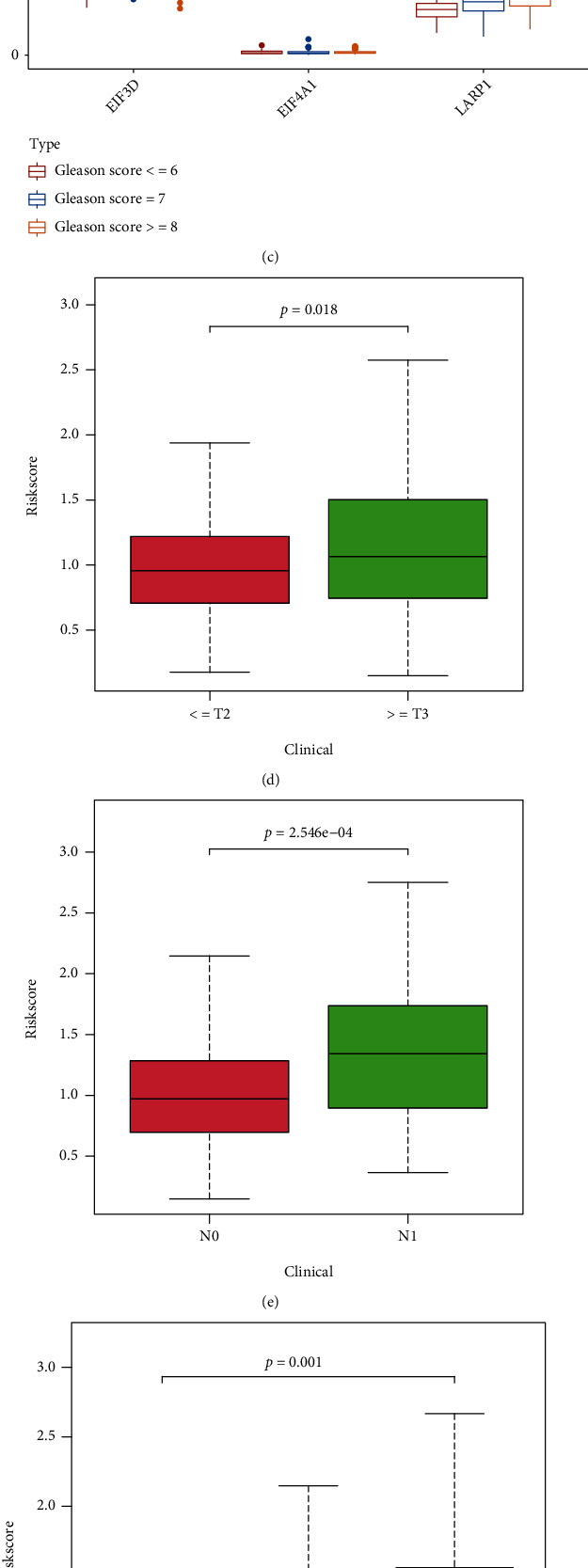
Gene expression from the signature in patients with PRAD. (a–c) The correlation between gene expression and pathologic T and N stages, as well as Gleason score, of PRAD. (d–f) Relationship between this signature and clinical features of PRAD. (g) Immunohistochemistry figures from The Human Protein Atlas database of these genes.

The correlation analysis was employed between the clinical characteristics and the expression of EIF3D, EIF4A1, and LARP1 in TCGA cohort, the risk score as well. As shown in Figures 6(a)–6(c), T classification and lymph node involvement were significantly associated with EIF3D expression; a significant association was also identified between LRP1 expression and T classification, lymph node involvement, and Gleason score (*p* < 0.05). Furthermore, risk scores were also notably correlated to T-staging in pathology, N-staging in pathology, and Gleason score for progression of PRAD (Figures 6(d)–6(f)). In accordance with the Human Protein Atlas database, we discovered that EIF3D, EIF4A1, and LARP1 were all upregulated in PRAD tissues (Figure 6(g)).

### 3.6. Functional Enrichment and Immune Activity Analysis

**Figure 7:**
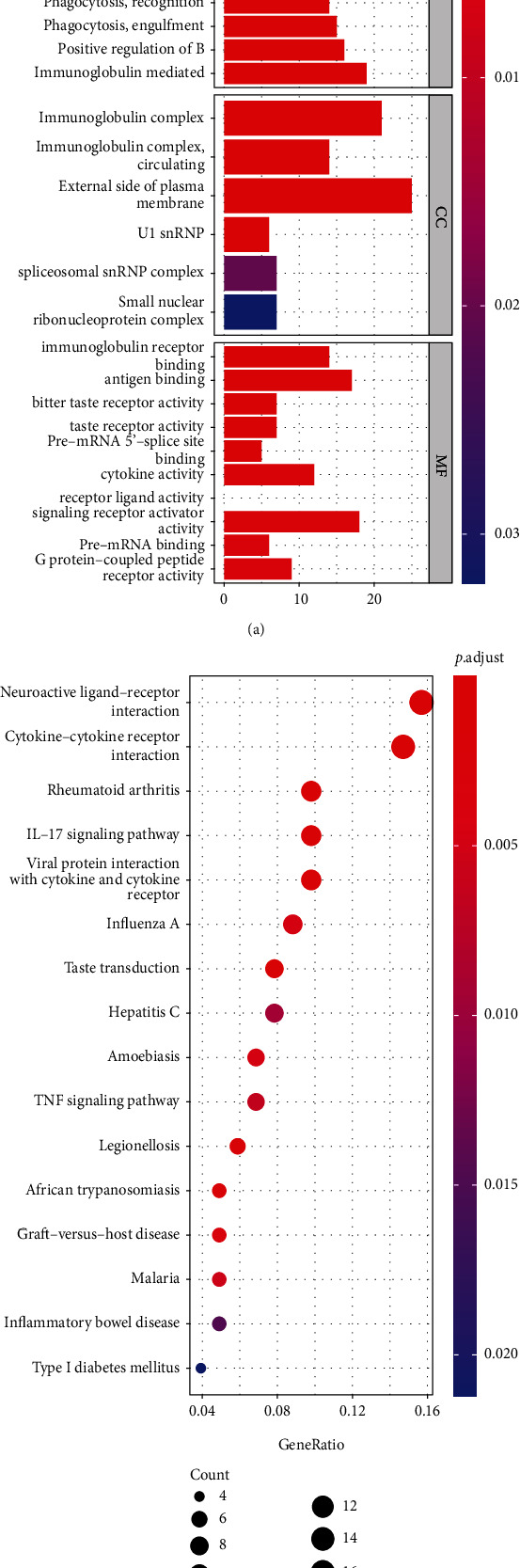
Functional analysis of KEGG and GO in TCGA. (a) Barplot graph for GO analysis (bar length means degree of enrichment and color means degree of difference). (b) Bubble graph for KEGG enrichment (bigger bubbles indicate more enriched genes, whereas deeper red means more notable differences).

Differential analysis was conducted by “limma” R package to extract genes which were differentially expressed between different risk groups to explore signaling functions and pathways. 2219 DEGs were identified in TCGA cohort under the criteria: FDR < 0.05, as well as |log2FC| ≥ 1, including 19 downregulated genes and 2200 upregulated genes in the high-risk cluster. Through these DEGs, enrichment analysis of KEGG and GO indicated that these genes were closely correlated with humoral immune response regulated by circulating immunoglobulin, immunoglobulin complex, immunoglobulin receptor binding, and neuroactive ligand-receptor interaction (Figures 7(a) and 7(b)).

**Figure 8:**
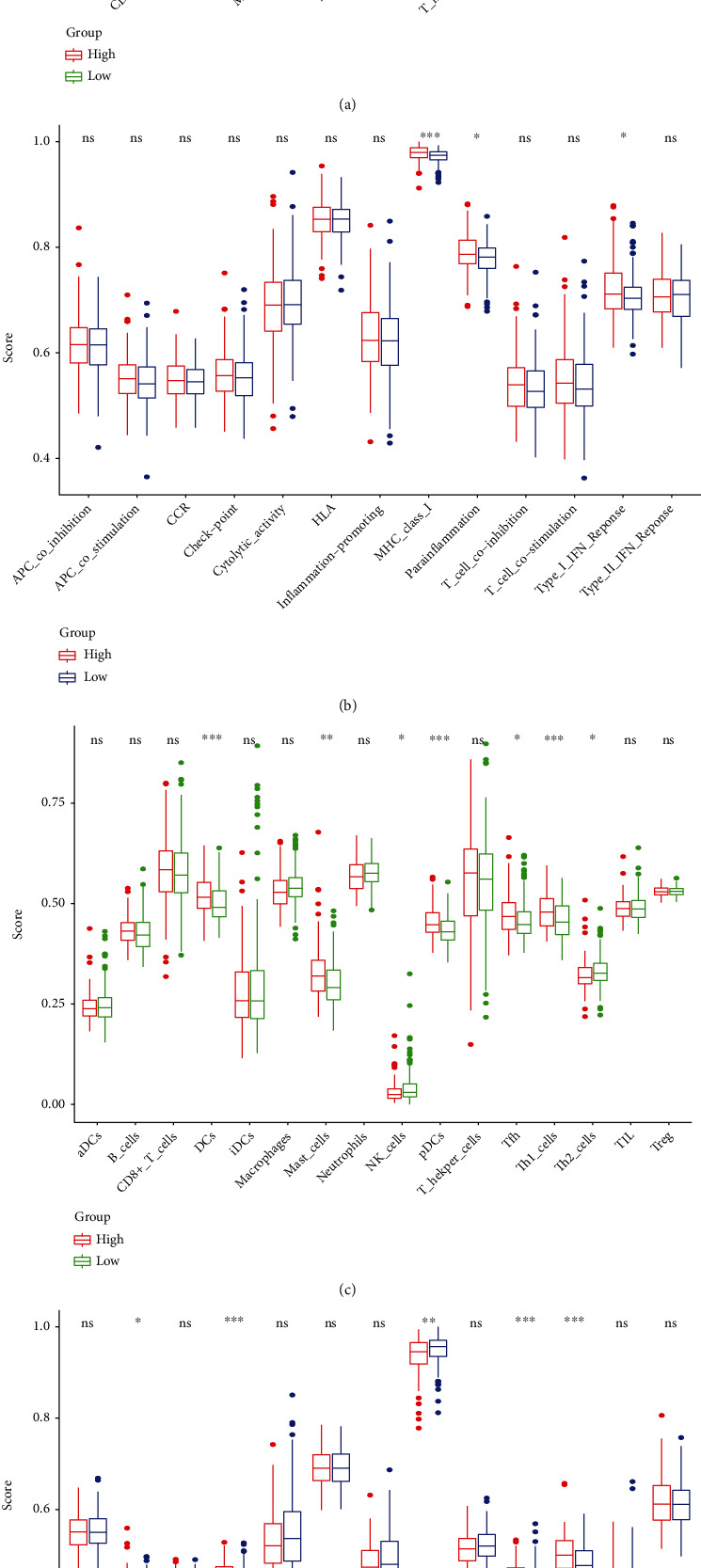
Correlation analysis between ssGSEA scores and immune cells or immune pathways. (a and b) A comparison of enrichment scores of immune cells and pathways related to immune in TCGA cohort (groups in green are at low risk; groups in red are at high risk). (c and d) A comparison of enrichment scores across GEO cohorts.

**Figure 9:**
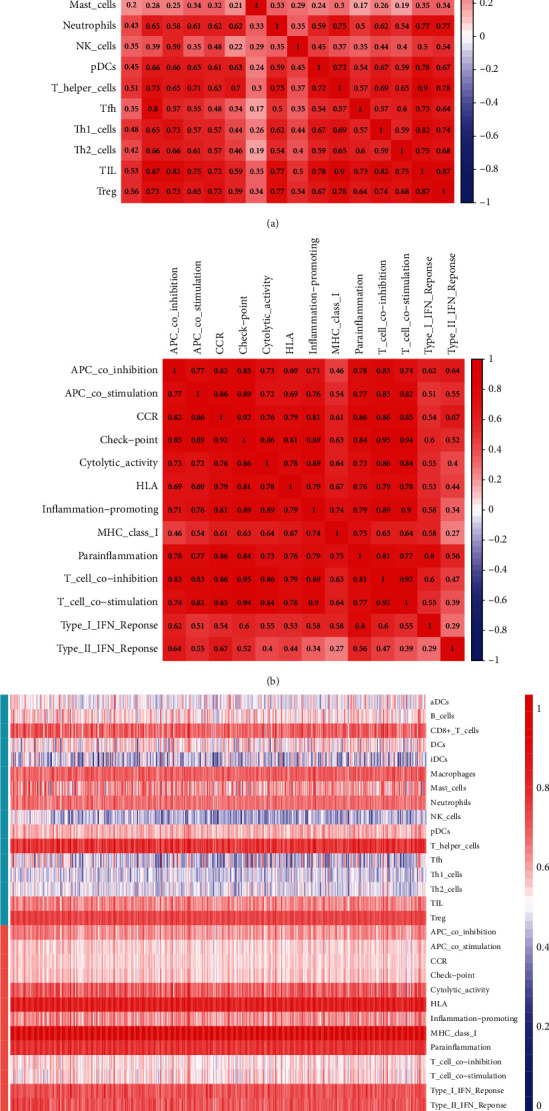
The results of immunocorrelation analysis. (a and b) Correlation between immune cells and pathways related to immune (the redder the color, the higher the correlation). (c) The heat map shows the infiltration of immune cells and pathways related to immunity.

ssGSEA analysis demonstrated a correlation between TCGA and GEO for 16 immune cell infiltration values and 13 immune-related pathway activities. Results from TCGA cohort analysis illustrated that patients with higher risk scores had less immune cell infiltration compared with low-risk participants, such as aDCs, neutrophils, and Th1 cells (Figure 8(a)). In immune-related pathways activity analysis, MHC (major histocompatibility complex) class I, parainflammation, and type I IFN (interferon) response showed the lower activity in high expression group (Figure 8(b)). Additionally, in GEO cohort, we found that DCs (dendritic cells), mast cells, pDCs (plasmacytoid DCs), and Th (T helper) cells had significant difference in subgroups (Figure 8(c)). Immune-related pathways, such as APC (antigen-presenting cell) co-stimulation, check point, and T cell co-stimulation, also had significant differences (Figure 8(d)). Then, the correlation of infiltrating immune subsets between different immune cells and pathways related to immune is demonstrated in Figures 9(a) and 9(b). The closer the correlation coefficient was to 1, the higher the correlation between immune cells or immune-related pathways. A heat map illustrated that T helper cells, HLA (human leukocyte antigen), and parainflammation were significantly associated with PRAD samples.

### 3.7. Expression of Genes with Sensitivity of Cancer Cells to Chemotherapy

**Figure 10:**
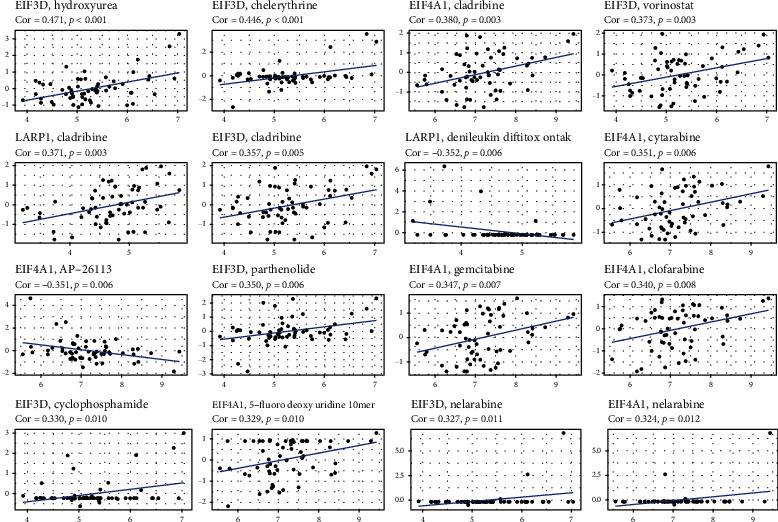
The relationship between 3 genes in the signature and the drug sensitivity.

Pearson's correlation analysis was conducted to assess whether 3 genes within NCI-60 cell lines have a relationship with drug sensitivity and there was a *p*-value < 0.01. The EIF3D, EIF4A1, and LARP1 genes have all been implicated in chemotherapeutic drug sensitivity, such as hydroxyurea, chelerythrine, methylprednisolone, cladribine, and cytarabine (Figure 10). EIF3D was identified to enhance the sensitivity of cancer cells to some chemotherapeutics, such as hydroxyurea, chelerythrine, and vorinostat, while LARP1 weakened the sensitivity of denileukin diftitox ontak.

## 4. Discussion

In our study, the differentially expressed m^7^G-related genes were analyzed between normal prostate tissues and prostate cancer tissues in TCGA, and 16 m^7^G-related genes were identified as mDEGs. Univariate and lasso Cox regression analyses were then utilized to develop this 3-gene risk signature that assessed the prognostic relevance of genes which were significantly connected with m^7^G. Taking outcomes of our study into account, we believe that this signature could be regarded as a prognostic factor to predict the BFFS for PRAD patients independently. Then, the function enrichment analysis presents that this prognostic signature was observably related with immune cells and immune-related pathways. Additionally, analysis of drug sensitivity indicated that EIF3D, EIF4A1, and LARP1 expressed by cancer cells greatly influenced the sensitivity of the cells to chemotherapy.

The mRNA cap m^7^G is reported as a ubiquitous positively charged modification [7] and can modulate nearly all stages of mRNA synthesis. However, how m^7^G-related genes related with the occurrence and progression of PRAD, as well as the survival time of PRAD patients, was still uncertain. Based on 3 genes associated with the m^7^G sequence, this study developed a signature, including EIF3D, EIF4A1, and LARP1. Later, we found the signature could predict the BFFS of PRAD patients. EIF3 is the largest of the ribosomal EIF complexes that bind to the 40S ribosome and maintains dissociation of the 40S and 60S ribosomal subunits. According to some previous studies, EIF3D, the largest subunit of EIF3, regulates the stability of the association between EIF3 subunits [28]. EIF3D has also been reported to play some important role in tumors, such as colon cancer [29], melanoma [30] and breast cancer [31]. Recent studies concluded that the EIF3D expression is critical to the occurrence of cancer by promoting protein synthesis. Overexpression and activity of EIF4A1, which is an ATP-dependent RNA helicase, have been linked to the development of certain tumors [32]. In ATP-dependent protein translation, EIF4A1 catalyzes unwinding of mRNA's 5′ UTR, an essential step in RNA processing, prior to ribosome binding, especially for mRNAs with increased lengths and complexity of the 5′ UTR. Previous studies over the past decade had proved the antitumor toxicity of EIF4A1 inhibition, maintaining a therapeutic window for normal cells [32, 33]. LARP1 had also been reported to have significant correlation with some tumor, such as colorectal cancer [34] and ovarian cancer [35]. What has been illustrated is that LRP1 complexes with PABP (poly-A binding protein) and binds an interactome of over 3000 mRNAs, among them transcripts encoding the ribosomal machinery, which lack the 5′ TOP motif [36]. Furthermore, LARP1 had been shown to contribute significantly to ribosome biogenesis through its involvement in mTOR signaling [37]. A m^7^G group or derivative at the 5′ end of an RNA molecule showed selective and noncovalent interactions with EIF3D, EIF4A1, and LARP1, according to the gene sets “GOMF RNA CAP BINDING.” In summary, EIF3D, EIF4A1, and LARP1 in the prognostic model were proved to be m^7^G-related genes. Our study showed that not every gene we examined was related to a better PRAD prognosis. Thus, the specific mechanism of these genes interacting with each other during m^7^G remains to be further explored.

Up to now, the research on mechanism of m^7^G-related genes in PRAD is still insufficient. Our study identified that EIF3D, EIF4A1, and LARP1 may be key m^7^G-related genes in PRAD. In our analyses of prognostic value of these genes, we identified a signature that may function as a prognostic factor independently. Moreover, immunoactivity analysis and function enrichment were also employed to investigate the possible functions of this signature. However, the specific mechanism of m^7^G-related genes, including EIF3D, EIF4A1, and LARP1, affecting the occurrence, development, and prognosis of PRAD deserves further research.

In conclusion, the study presented that m^7^G was closely related with PRAD, because for the expression of most of the genes related to m^7^G, there was a great difference between tumor and normal prostate tissues. Aside from that, the score calculated from the 3 m^7^G-related genes signature had significant correlation with the prognosis of PRAD patient. Also, the model may be a method for predicting BFFS independently in TCGA cohort and GEO cohort for PRAD patients. On the other hand, immunity was associated with DEGs which was identified from different risk clusters. In our study, a novel prognostic signature was identified to predict the BFFS for PRAD patients, and the genes EIF3D, EIF4A1, and LARP1, related to m^7^G, were identified as the genes representing m^7^G-related genes in PRAD for further mechanisms research. In addition, the study provides a well condition to investigate the regulating relationship between m^7^G and immunity in PRAD.

## 5. Conclusion

In our study, multiple bioinformatics analyses were utilized to identify a 3-gene signature related with m^7^G to evaluate the prognosis of PRAD patient. Our study found that the signature was significantly related with the prostate cancer clinical characteristics and immunity. Thus, the gene signature in our study could be utilized as an independent prognostic indicator for PRAD patients.

## Figures and Tables

**Table 1:** Clinical characteristics of the PRAD patients in different risk groups.

Clinical characteristics (samples)	TCGA cohort (339)		GSE116918 cohort (248)	
	Low risk	High risk	Low risk	High risk
Age (years)				
<65	156	70	47	28
≥65	70	43	118	55
Pathologic N				
N0	199	82		
N1	27	31		
Pathologic T				
T1+T2	115	28		
T3+T4	111	85		
Gleason score				
<=6	14	5	26	16
=7	126	44	58	41
>=8	86	64	81	26
PSA (ng/ml)				
<20			96	46
>=20			69	37

## Data Availability

The data could be acquired from TCGA dataset (https://portal.gdc.cancer.gov/) and GEO dataset (https://www.ncbi.nlm.nih.gov/geo/). The data of drug sensitivity could be acquired from the CellMiner database (https://discover.nci.nih.gov/cellminer). The code used in the study is available from the corresponding author on reasonable request.

## Abbreviations

m^7^G: N7-methylguanine
PRAD: Prostate adenocarcinoma
BCR: Biochemical recurrence
BFFS: Biochemical failure-free survival
RNA-seq: RNA sequencing
mDEGs: m^7^G-related differentially expressed genes
GO: Gene Ontology
KEGG: Kyoto Encyclopedia of Genes and Genomes
TCGA: The Cancer Genome Atlas
GEO: Gene Expression Omnibus
GSEA: Gene Set Enrichment Analysis
GSVA: Gene Set Variation Analysis
KM: Kaplan-Meier
PCA: Principal component analysis
ROC: Receiver operating characteristic
NCI: US National Cancer Institute.

## Supplementary Materials

Supplementary Materials Supplementary Table 1: These genes were m7G-related genes including 26 genes in (GSEA) database ((http://www.gsea-msigdb.org/gsea/index.jsp)), and 16 other genes from research previously published, which were utilized to construct the prognostic signature in PRAD. Supplementary Table 2: The results of differential expression analysis of 42 m7G-related genes, and 16 DEGs were identified with *p* < 0.05. Supplementary Table 3: Differential expression genes between high- and low-risk groups, which were selected for functional enrichment analysis and immunocorrelation analysis.
