# Supplementary material for: A N7-Methylguanine-Related Gene Signature Applicable for the Prognosis and Microenvironment of Prostate Cancer
Source: J Oncol. 2022 May 13;2022:8604216. doi: 10.1155/2022/8604216 (PMC9122703; doi:10.1155/2022/8604216)
Supplement: Supplementary Materials — Supplementary Table 1: These genes were m7G-related genes including 26 genes in (GSEA) database ((http://www.gsea-msigdb.org/gsea/index.jsp)), and 16 other genes from research previously published, which were utilized to construct the prognostic signature in PRAD. Supplementary Table 2: The results of differential expression analysis of 42 m7G-related genes, and 16 DEGs were identified with p < 0.05. Supplementary Table 3: Differential expression genes between high- and low-risk groups, which were selected for functional enrichment analysis and immunocorrelation analysis. [file 8604216.f1.zip › Supplementary table 1.docx]

M7G-related genes and their sourcs

| Gene ID | | Source |
| --- | --- | --- |
| METTL1 | Tomikawa C. 7-Methylguanosine Modifications in Transfer RNA (tRNA). Int J Mol Sci. 2018 Dec 17;19(12):4080. doi: 10.3390/ijms19124080. PMID: 30562954; PMCID: PMC6320965. | |
| WDR4 | Tomikawa C. 7-Methylguanosine Modifications in Transfer RNA (tRNA). Int J Mol Sci. 2018 Dec 17;19(12):4080. doi: 10.3390/ijms19124080. PMID: 30562954; PMCID: PMC6320966. | |
| NSUN2 | Tomikawa C. 7-Methylguanosine Modifications in Transfer RNA (tRNA). Int J Mol Sci. 2018 Dec 17;19(12):4080. doi: 10.3390/ijms19124080. PMID: 30562954; PMCID: PMC6320967. | |
| DCP2 | m7G genesets M26714.gmt | |
| DCPS | m7G genesets M26715.gmt | |
| NUDT10 | m7G genesets M26716.gmt | |
| NUDT11 | m7G genesets M26717.gmt | |
| NUDT16 | m7G genesets M26718.gmt | |
| NUDT3 | m7G genesets M26719.gmt | |
| NUDT4 | m7G genesets M26720.gmt | |
| NUDT4B | m7G genesets M26721.gmt | |
| AGO2 | m7G genesets M26066.gmt | |
| CYFIP1 | m7G genesets M26067.gmt | |
| EIF4E | m7G genesets M26068.gmt | |
| EIF4E1B | m7G genesets M26069.gmt | |
| EIF4E2 | m7G genesets M26070.gmt | |
| EIF4E3 | m7G genesets M26071.gmt | |
| GEMIN5 | m7G genesets M26072.gmt | |
| LARP1 | m7G genesets M26073.gmt | |
| NCBP1 | m7G genesets M26074.gmt | |
| NCBP2 | m7G genesets M26075.gmt | |
| NCBP3 | m7G genesets M26076.gmt | |
| EIF3D | m7G genesets M18244.gmt | |
| EIF4A1 | m7G genesets M18245.gmt | |
| EIF4G3 | m7G genesets M18246.gmt | |
| IFIT5 | m7G genesets M18247.gmt | |
| LSM1 | m7G genesets M18248.gmt | |
| NCBP2L | m7G genesets M18249.gmt | |
| SNUPN | m7G genesets M18250.gmt | |
| PHAX | Boulon S, Verheggen C, Jady BE, Girard C, Pescia C, Paul C, Ospina JK, Kiss T, Matera AG, Bordonné R, Bertrand E. PHAX and CRM1 are required sequentially to transport U3 snoRNA to nucleoli. Mol Cell. 2004 Dec 3;16(5):777-87. doi: 10.1016/j.molcel.2004.11.013. PMID: 15574332. | |
| DXO | Jiao X, Doamekpor SK, Bird JG, Nickels BE, Tong L, Hart RP, Kiledjian M. 5' End Nicotinamide Adenine Dinucleotide Cap in Human Cells Promotes RNA Decay through DXO-Mediated deNADding. Cell. 2017 Mar 9;168(6):1015-1027.e10. doi: 10.1016/j.cell.2017.02.019. PMID: 28283058; PMCID: PMC5371429. | |
| TGS1 | Monecke T, Dickmanns A, Ficner R. Structural basis for m7G-cap hypermethylation of small nuclear, small nucleolar and telomerase RNA by the dimethyltransferase TGS1. Nucleic Acids Res. 2009 Jul;37(12):3865-77. doi: 10.1093/nar/gkp249. Epub 2009 Apr 22. PMID: 19386620; PMCID: PMC2709555. | |
| NUDT12 | Wu H, Li L, Chen KM, Homolka D, Gos P, Fleury-Olela F, McCarthy AA, Pillai RS. Decapping Enzyme NUDT12 Partners with BLMH for Cytoplasmic Surveillance of NAD-Capped RNAs. Cell Rep. 2019 Dec 24;29(13):4422-4434.e13. doi: 10.1016/j.celrep.2019.11.108. PMID: 31875550. | |
| XPO1 | Sheng P, Fields C, Aadland K, Wei T, Kolaczkowski O, Gu T, Kolaczkowski B, Xie M. Dicer cleaves 5'-extended microRNA precursors originating from RNA polymerase II transcription start sites. Nucleic Acids Res. 2018 Jun 20;46(11):5737-5752. doi: 10.1093/nar/gky306. PMID: 29746670; PMCID: PMC6009592. | |
| IPO8 | Volpon L, Culjkovic-Kraljacic B, Osborne MJ, Ramteke A, Sun Q, Niesman A, Chook YM, Borden KL. Importin 8 mediates m7G cap-sensitive nuclear import of the eukaryotic translation initiation factor eIF4E. Proc Natl Acad Sci U S A. 2016 May 10;113(19):5263-8. doi: 10.1073/pnas.1524291113. Epub 2016 Apr 25. PMID: 27114554; PMCID: PMC4868427. | |
| EIF4G1 | Haimov O, Sehrawat U, Tamarkin-Ben Harush A, Bahat A, Uzonyi A, Will A, Hiraishi H, Asano K, Dikstein R. Dynamic Interaction of Eukaryotic Initiation Factor 4G1 (eIF4G1) with eIF4E and eIF1 Underlies Scanning-Dependent and -Independent Translation. Mol Cell Biol. 2018 Aug 28;38(18):e00139-18. doi: 10.1128/MCB.00139-18. PMID: 29987188; PMCID: PMC6113598. | |
| EIF1 | Haimov O, Sehrawat U, Tamarkin-Ben Harush A, Bahat A, Uzonyi A, Will A, Hiraishi H, Asano K, Dikstein R. Dynamic Interaction of Eukaryotic Initiation Factor 4G1 (eIF4G1) with eIF4E and eIF1 Underlies Scanning-Dependent and -Independent Translation. Mol Cell Biol. 2018 Aug 28;38(18):e00139-18. doi: 10.1128/MCB.00139-18. PMID: 29987188; PMCID: PMC6113598. | |
| PARN | Martînez J, Ren YG, Nilsson P, Ehrenberg M, Virtanen A. The mRNA cap structure stimulates rate of poly(A) removal and amplifies processivity of degradation. J Biol Chem. 2001 Jul 27;276(30):27923-9. doi: 10.1074/jbc.M102270200. Epub 2001 May 18. PMID: 11359775. | |
| CCNB1 | Xia P, Zhang H, Xu K, Jiang X, Gao M, Wang G, Liu Y, Yao Y, Chen X, Ma W, Zhang Z, Yuan Y. MYC-targeted WDR4 promotes proliferation, metastasis, and sorafenib resistance by inducing CCNB1 translation in hepatocellular carcinoma. Cell Death Dis. 2021 Jul 9;12(7):691. doi: 10.1038/s41419-021-03973-5. PMID: 34244479; PMCID: PMC8270967. | |
| CDK1 | Aregger M, Kaskar A, Varshney D, Fernandez-Sanchez ME, Inesta-Vaquera FA, Weidlich S, Cowling VH. CDK1-Cyclin B1 Activates RNMT, Coordinating mRNA Cap Methylation with G1 Phase Transcription. Mol Cell. 2016 Mar 3;61(5):734-746. doi: 10.1016/j.molcel.2016.02.008. PMID: 26942677; PMCID: PMC4781437. | |
| APAF1 | Andreev DE, Dmitriev SE, Zinovkin R, Terenin IM, Shatsky IN. The 5' untranslated region of Apaf-1 mRNA directs translation under apoptosis conditions via a 5' end-dependent scanning mechanism. FEBS Lett. 2012 Nov 30;586(23):4139-43. doi: 10.1016/j.febslet.2012.10.010. Epub 2012 Oct 17. PMID: 23085065. | |
| JUND | Vesely PW, Staber PB, Hoefler G, Kenner L. Translational regulation mechanisms of AP-1 proteins. Mutat Res. 2009 Jul-Aug;682(1):7-12. doi: 10.1016/j.mrrev.2009.01.001. Epub 2009 Jan 9. PMID: 19167516. | |
