# Supplementary material for: A N7-Methylguanine-Related Gene Signature Applicable for the Prognosis and Microenvironment of Prostate Cancer
Source: J Oncol. 2022 May 13;2022:8604216. doi: 10.1155/2022/8604216 (PMC9122703; doi:10.1155/2022/8604216)
Supplement: Supplementary Materials — Supplementary Table 1: These genes were m7G-related genes including 26 genes in (GSEA) database ((http://www.gsea-msigdb.org/gsea/index.jsp)), and 16 other genes from research previously published, which were utilized to construct the prognostic signature in PRAD. Supplementary Table 2: The results of differential expression analysis of 42 m7G-related genes, and 16 DEGs were identified with p < 0.05. Supplementary Table 3: Differential expression genes between high- and low-risk groups, which were selected for functional enrichment analysis and immunocorrelation analysis. [file 8604216.f1.zip › Supplementary table 2.docx]

The differential analysis of m7G-related genes

| gene | group1mea | group2mea | logFC | p | FDR |
| --- | --- | --- | --- | --- | --- |
| AGO2 | 2.571223 | 2.926097 | 0.186523 | 0.415023 | 0.495722 |
| APAF1 | 2.072807 | 1.87749 | -0.14278 | 0.117862 | 0.168936 |
| CCNB1 | 2.561822 | 4.76841 | 0.896338 | 1.20E-14 | 1.04E-13 |
| CDK1 | 1.101312 | 2.314684 | 1.071591 | 7.77E-09 | 2.78E-08 |
| CYFIP1 | 14.3607 | 16.05459 | 0.16086 | 0.009729 | 0.015495 |
| DCP2 | 3.007379 | 3.142589 | 0.063447 | 0.806576 | 0.845921 |
| DCPS | 3.966527 | 4.107365 | 0.050337 | 0.294413 | 0.372346 |
| DXO | 6.44278 | 9.510091 | 0.561776 | 5.92E-16 | 8.49E-15 |
| EIF1 | 135.8764 | 140.0467 | 0.043614 | 0.397949 | 0.488908 |
| EIF3D | 53.0064 | 65.55749 | 0.306594 | 2.65E-08 | 8.50E-08 |
| EIF4A1 | 0.88822 | 1.268703 | 0.514366 | 3.31E-07 | 9.48E-07 |
| EIF4E | 2.326483 | 2.334482 | 0.004952 | 0.947091 | 0.969641 |
| EIF4E1B | 0.00544 | 0.002265 | -1.26442 | 0.464142 | 0.525213 |
| EIF4E2 | 6.764914 | 8.314667 | 0.297587 | 1.75E-11 | 9.42E-11 |
| EIF4E3 | 5.050116 | 3.143453 | -0.683967 | 8.23E-10 | 3.54E-09 |
| EIF4G1 | 46.30774 | 63.35952 | 0.452308 | 3.74E-12 | 2.30E-11 |
| EIF4G3 | 9.195525 | 9.229844 | 0.005374 | 0.982109 | 0.982109 |
| GEMIN5 | 5.151769 | 4.881377 | -0.07778 | 0.080184 | 0.118894 |
| IFIT5 | 5.483723 | 3.992399 | -0.4579 | 2.77E-08 | 8.50E-08 |
| IPO8 | 9.992004 | 8.49752 | -0.233732 | 1.30E-05 | 3.49E-05 |
| JUND | 116.4773 | 131.209 | 0.171818 | 0.243386 | 0.317139 |
| LARP1 | 19.88236 | 25.9111 | 0.382081 | 3.72E-09 | 1.46E-08 |
| LSM1 | 11.61034 | 10.98272 | -0.080174 | 0.040649 | 0.062425 |
| METTL1 | 4.716647 | 7.314534 | 0.633004 | 1.03E-16 | 2.21E-15 |
| NCBP1 | 6.806509 | 7.244989 | 0.090068 | 0.167638 | 0.23253 |
| NCBP2 | 14.2084 | 17.55836 | 0.305415 | 1.86E-10 | 8.86E-10 |
| NCBP2L | 0.159213 | 0.250791 | 0.655523 | 0.006246 | 0.010329 |
| NCBP3 | 3.258981 | 3.236545 | -0.009967 | 0.759474 | 0.837369 |
| NSUN2 | 9.715724 | 13.02373 | 0.422749 | 2.23E-15 | 2.39E-14 |
| NUDT10 | 13.28153 | 5.49438 | -1.273393 | 3.06E-17 | 1.32E-15 |
| NUDT11 | 8.379146 | 10.59429 | 0.338413 | 0.175963 | 0.236451 |
| NUDT12 | 7.196871 | 6.000335 | -0.262327 | 0.004515 | 0.007765 |
| NUDT16 | 9.003927 | 10.49892 | 0.221614 | 1.48E-05 | 3.74E-05 |
| NUDT3 | 2.741631 | 2.368334 | -0.211162 | 0.000107 | 0.000242 |
| NUDT4 | 10.05808 | 14.37971 | 0.51568 | 0.003135 | 0.006128 |
| NUDT4B | 0.00886 | 0.013908 | 0.650427 | 0.443412 | 0.515316 |
| PARN | 12.44459 | 11.65834 | -0.094156 | 0.000733 | 0.001575 |
| PHAX | 9.551507 | 8.737936 | -0.128436 | 0.003346 | 0.006255 |
| SNUPN | 4.598192 | 5.002224 | 0.121503 | 0.004299 | 0.007703 |
| TGS1 | 6.196601 | 5.345359 | -0.21319 | 0.000807 | 0.001652 |
| WDR4 | 2.23792 | 3.230279 | 0.5295 | 2.12E-12 | 1.52E-11 |
| XPO1 | 11.00814 | 11.4484 | 0.056576 | 0.793139 | 0.845921 |
