# Supplementary material for: A N7-Methylguanine-Related Gene Signature Applicable for the Prognosis and Microenvironment of Prostate Cancer
Source: J Oncol. 2022 May 13;2022:8604216. doi: 10.1155/2022/8604216 (PMC9122703; doi:10.1155/2022/8604216)
Supplement: Supplementary Materials — Supplementary Table 1: These genes were m7G-related genes including 26 genes in (GSEA) database ((http://www.gsea-msigdb.org/gsea/index.jsp)), and 16 other genes from research previously published, which were utilized to construct the prognostic signature in PRAD. Supplementary Table 2: The results of differential expression analysis of 42 m7G-related genes, and 16 DEGs were identified with p < 0.05. Supplementary Table 3: Differential expression genes between high- and low-risk groups, which were selected for functional enrichment analysis and immunocorrelation analysis. [file 8604216.f1.zip › Supplementary table 3.docx]

Differential analysis between high and low risk groups

| gene | group1mea | group2mea | logFC | p | FDR |
| --- | --- | --- | --- | --- | --- |
| LDHAL6EP | 0.052582 | 0.159181 | 1.598021 | 0.00012 | 0.000473 |
| RNU6-377P | 0.079499 | 0.350573 | 2.140707 | 3.92E-07 | 3.19E-06 |
| AC005912.2 | 0.005965 | 0.033202 | 2.476786 | 0.005745 | 0.013308 |
| PROK1 | 3.143078 | 1.568761 | -1.00255 | 9.05E-06 | 4.97E-05 |
| RF00015 | 0.107993 | 0.221179 | 1.034272 | 4.61E-11 | 1.17E-09 |
| ZNF29P | 0.094328 | 0.285853 | 1.599506 | 0.021755 | 0.041244 |
| HIST1H1B | 0.070673 | 0.174266 | 1.302068 | 0.022188 | 0.041938 |
| AC092718.7 | 0.160979 | 0.348977 | 1.116259 | 1.63E-14 | 1.18E-12 |
| SNORA55 | 0.541699 | 1.679983 | 1.632883 | 3.48E-19 | 1.52E-16 |
| AC010653.1 | 0.040186 | 0.08411 | 1.065583 | 5.50E-06 | 3.24E-05 |
| AP001574.1 | 0.084318 | 0.175207 | 1.055148 | 0.022075 | 0.041759 |
| MIR3136 | 0.04643 | 0.160414 | 1.788669 | 1.45E-12 | 5.86E-11 |
| RNA5SP462 | 0.168638 | 0.368475 | 1.127634 | 2.97E-06 | 1.90E-05 |
| HORMAD1 | 0.012016 | 0.029714 | 1.306235 | 0.007958 | 0.017593 |
| AL139132.1 | 0.050266 | 0.130421 | 1.375506 | 3.75E-05 | 0.000171 |
| AL583844.1 | 0.009625 | 0.019937 | 1.050592 | 0.014562 | 0.029392 |
| AL353689.1 | 0.036671 | 0.251532 | 2.778045 | 2.39E-09 | 3.71E-08 |
| AC068870.3 | 0.004002 | 0.056905 | 3.829818 | 1.88E-06 | 1.26E-05 |
| TAS2R31 | 0.157723 | 0.316604 | 1.005284 | 1.70E-06 | 1.16E-05 |
| AC005899.8 | 0.015219 | 0.053145 | 1.804015 | 3.97E-07 | 3.23E-06 |
| AC006116.5 | 0.04684 | 0.12197 | 1.380716 | 0.026651 | 0.048892 |
| RF00156 | 0.014951 | 0.059303 | 1.987835 | 0.000253 | 0.000898 |
| AC083798.1 | 0.34721 | 0.790336 | 1.186657 | 3.43E-07 | 2.85E-06 |
| AL445670.1 | 0.021592 | 0.04741 | 1.134669 | 5.08E-07 | 4.01E-06 |
| AC003682.1 | 0.051343 | 0.113307 | 1.141993 | 9.18E-09 | 1.21E-07 |
| AC134407.3 | 0.056066 | 0.165712 | 1.563489 | 1.27E-17 | 2.99E-15 |
| AC005522.1 | 0.024667 | 0.086067 | 1.80289 | 0.012089 | 0.025131 |
| AC087392.4 | 0.165542 | 0.372746 | 1.170995 | 2.79E-10 | 5.68E-09 |
| LRRTM2 | 0.029358 | 0.095994 | 1.709201 | 3.40E-05 | 0.000157 |
| AL590632.1 | 0.018566 | 0.097909 | 2.398795 | 5.22E-07 | 4.10E-06 |
| RNU4-82P | 0.089406 | 0.381511 | 2.093275 | 6.20E-12 | 2.05E-10 |
| DIP2A-IT1 | 0.074424 | 0.400085 | 2.426467 | 1.54E-09 | 2.55E-08 |
| AC139792.1 | 0.017768 | 0.04383 | 1.302687 | 1.25E-06 | 8.81E-06 |
| AC022973.4 | 0.077568 | 0.190784 | 1.298414 | 2.03E-17 | 4.44E-15 |
| TH2LCRR | 0.091359 | 0.205223 | 1.167574 | 2.10E-14 | 1.49E-12 |
| AC100757.1 | 0.007827 | 0.018629 | 1.250907 | 0.000328 | 0.001126 |
| SRP14P3 | 0.029835 | 0.088741 | 1.572575 | 0.00459 | 0.010998 |
| MIR23A | 0.998128 | 3.109748 | 1.639501 | 3.02E-06 | 1.92E-05 |
| AC131649.2 | 0.108864 | 0.29813 | 1.45342 | 5.20E-12 | 1.76E-10 |
| AC087521.1 | 0.130855 | 0.274133 | 1.06691 | 0.001051 | 0.003097 |
| AC114402.1 | 0.145511 | 0.493763 | 1.762688 | 1.63E-11 | 4.78E-10 |
| AL358075.1 | 0.047735 | 0.111909 | 1.229214 | 8.02E-05 | 0.000332 |
| LINC02390 | 0.022561 | 0.07224 | 1.678989 | 0.000133 | 0.000517 |
| AC009093.4 | 0.053807 | 0.12938 | 1.265753 | 4.13E-05 | 0.000186 |
| DEFB109A | 0.10523 | 0.235551 | 1.162495 | 0.004806 | 0.011442 |
| RF01241 | 0.201046 | 0.438427 | 1.124812 | 3.27E-20 | 2.43E-17 |
| RNU1-124P | 0.059278 | 0.376499 | 2.667076 | 2.77E-05 | 0.000132 |
| AL354919.2 | 0.024457 | 0.054094 | 1.145228 | 0.025456 | 0.047087 |
| AL807761.4 | 0.004926 | 0.024121 | 2.291821 | 0.003988 | 0.009744 |
| RNU6-33P | 0.129667 | 0.337232 | 1.37893 | 0.000492 | 0.001607 |
| AL031005.1 | 0.023674 | 0.218573 | 3.206737 | 0.001123 | 0.003282 |
| RUFY4 | 0.167802 | 0.340948 | 1.02279 | 8.03E-06 | 4.47E-05 |
| ACAP2-IT1 | 0.148581 | 0.306318 | 1.043786 | 1.70E-16 | 2.65E-14 |
| AL035448.1 | 0.06362 | 0.18124 | 1.51036 | 9.40E-13 | 4.00E-11 |
| AC127164.1 | 0.030089 | 0.071031 | 1.239216 | 1.60E-08 | 1.98E-07 |
| RNA5SP187 | 0.376022 | 1.328235 | 1.820621 | 2.52E-11 | 6.91E-10 |
| AP001207.2 | 0.039232 | 0.356539 | 3.183951 | 5.12E-08 | 5.47E-07 |
| AC245100.2 | 0.211534 | 0.515665 | 1.285546 | 1.71E-20 | 1.47E-17 |
| AC002543.1 | 0.00584 | 0.033571 | 2.523051 | 6.15E-06 | 3.57E-05 |
| AC073130.2 | 0.065867 | 0.218118 | 1.727484 | 8.75E-08 | 8.75E-07 |
| AL034399.2 | 0.00779 | 0.031957 | 2.036378 | 0.000567 | 0.001817 |
| AC103739.1 | 0.106085 | 0.34348 | 1.69501 | 9.42E-05 | 0.000382 |
| SLC28A3 | 0.033382 | 0.090836 | 1.444181 | 0.008413 | 0.018452 |
| AL109615.2 | 0.011348 | 0.0229 | 1.012872 | 0.016178 | 0.032163 |
| MAPRE1P1 | 0.025179 | 0.055833 | 1.148873 | 0.000103 | 0.000411 |
| RSAD2 | 1.216385 | 2.836679 | 1.221603 | 9.23E-05 | 0.000375 |
| AC005838.2 | 0.199146 | 0.487516 | 1.291624 | 3.59E-06 | 2.23E-05 |
| AC011939.2 | 0.02066 | 0.491394 | 4.57195 | 1.43E-08 | 1.79E-07 |
| AL137222.1 | 0.013838 | 0.037722 | 1.446729 | 0.000474 | 0.001552 |
| AC034102.6 | 0.069873 | 0.141209 | 1.015036 | 2.46E-17 | 5.34E-15 |
| AL954705.1 | 0.318135 | 0.799013 | 1.328582 | 1.34E-15 | 1.48E-13 |
| AP002518.1 | 0.012678 | 0.03121 | 1.299734 | 0.00362 | 0.008975 |
| AC006441.1 | 0.035376 | 0.113799 | 1.685648 | 0.00172 | 0.004731 |
| AP001429.1 | 0.188613 | 0.576054 | 1.610775 | 2.73E-05 | 0.00013 |
| RN7SL559P | 0.056455 | 0.247082 | 2.129823 | 1.47E-09 | 2.45E-08 |
| AL139811.1 | 0.035881 | 0.10611 | 1.564259 | 4.91E-09 | 6.98E-08 |
| TRBV21-1 | 0.050043 | 0.101274 | 1.017007 | 4.10E-05 | 0.000185 |
| MIR6740 | 0.095239 | 0.255894 | 1.425925 | 1.26E-05 | 6.64E-05 |
| AP005899.1 | 0.256635 | 0.6481 | 1.336501 | 9.64E-18 | 2.42E-15 |
| MIR3138 | 0.112019 | 0.265115 | 1.242874 | 4.99E-06 | 2.96E-05 |
| RF01210 | 0.017274 | 0.048909 | 1.501513 | 2.77E-09 | 4.22E-08 |
| BBIP1P1 | 0.198287 | 0.406335 | 1.035078 | 1.61E-11 | 4.72E-10 |
| AP001775.1 | 0.082391 | 0.171104 | 1.054309 | 0.00071 | 0.002207 |
| MIR3164 | 0.173699 | 0.421836 | 1.280095 | 1.60E-06 | 1.09E-05 |
| RN7SKP287 | 0.085664 | 0.284577 | 1.732059 | 1.20E-08 | 1.54E-07 |
| KLF7-IT1 | 0.134496 | 0.436793 | 1.699391 | 1.72E-06 | 1.17E-05 |
| AL590729.1 | 0.082864 | 0.207344 | 1.32321 | 2.69E-13 | 1.34E-11 |
| RN7SL600P | 0.070721 | 0.736086 | 3.379662 | 2.05E-09 | 3.24E-08 |
| LINC01697 | 0.022494 | 0.092376 | 2.038005 | 0.002543 | 0.006609 |
| AC006449.3 | 0.041456 | 0.092432 | 1.156813 | 5.04E-07 | 3.99E-06 |
| AC018682.1 | 0.07594 | 0.489372 | 2.688003 | 1.20E-15 | 1.34E-13 |
| AC063950.1 | 0.103385 | 0.276827 | 1.42096 | 5.27E-05 | 0.00023 |
| KRT19P2 | 0.019752 | 0.063537 | 1.685618 | 0.001568 | 0.004369 |
| MIR3176 | 0.526894 | 1.108602 | 1.073157 | 1.86E-11 | 5.33E-10 |
| RPS20P4 | 0.140594 | 0.306562 | 1.12464 | 5.91E-05 | 0.000254 |
| GCNT7 | 0.097033 | 0.207453 | 1.096239 | 3.17E-11 | 8.42E-10 |
| HMGB3P4 | 0.058615 | 0.189141 | 1.69013 | 7.54E-08 | 7.67E-07 |
| MTCYBP21 | 0.03216 | 0.10832 | 1.751967 | 6.41E-13 | 2.87E-11 |
| RNU6-90P | 0.079848 | 0.250861 | 1.651566 | 4.81E-05 | 0.000212 |
| RN7SL381P | 0.30656 | 1.207661 | 1.977976 | 2.32E-22 | 7.73E-19 |
| AC068888.2 | 0.09338 | 0.250386 | 1.422964 | 3.91E-06 | 2.40E-05 |
| MYCBP2-AS1 | 0.034679 | 0.074472 | 1.102627 | 0.00785 | 0.017392 |
| AVPR1B | 0.046201 | 0.096132 | 1.057096 | 0.004943 | 0.011712 |
| HSPE1P18 | 0.053301 | 0.354582 | 2.733897 | 0.000605 | 0.001921 |
| AC019118.2 | 0.007529 | 0.02793 | 1.891279 | 5.69E-07 | 4.43E-06 |
| AL445231.1 | 0.15966 | 0.330172 | 1.048217 | 0.014656 | 0.029548 |
| AC005921.3 | 0.01871 | 0.098788 | 2.400516 | 2.99E-13 | 1.48E-11 |
| RNU4-8P | 0.0813 | 0.199689 | 1.296438 | 6.95E-10 | 1.27E-08 |
| RF00139 | 0.022328 | 0.069743 | 1.64323 | 2.84E-07 | 2.42E-06 |
| RN7SKP173 | 0.092007 | 0.202357 | 1.137085 | 2.43E-09 | 3.76E-08 |
| AC022395.1 | 0.020931 | 0.077032 | 1.879821 | 6.81E-10 | 1.25E-08 |
| AC108451.1 | 0.003494 | 0.014836 | 2.086083 | 0.000859 | 0.002605 |
| LINC00216 | 0.122202 | 0.337129 | 1.46404 | 1.02E-09 | 1.77E-08 |
| AC068189.1 | 0.017066 | 0.035929 | 1.074031 | 3.06E-05 | 0.000143 |
| RF02271 | 0.068218 | 0.179926 | 1.399184 | 4.52E-22 | 1.13E-18 |
| RF00394 | 0.025961 | 0.062933 | 1.277447 | 1.26E-05 | 6.64E-05 |
| AL133481.1 | 0.021623 | 0.083893 | 1.955989 | 1.20E-06 | 8.48E-06 |
| RNU6-930P | 0.229274 | 0.592249 | 1.369129 | 9.51E-10 | 1.67E-08 |
| AC012574.1 | 0.274309 | 0.590905 | 1.107123 | 6.72E-07 | 5.12E-06 |
| EDNRB-AS1 | 0.088272 | 0.317195 | 1.845351 | 0.00325 | 0.008187 |
| AL358232.1 | 0.097382 | 0.24647 | 1.339683 | 0.001063 | 0.003127 |
| SRP68P1 | 0.057726 | 0.124334 | 1.106931 | 0.000301 | 0.001047 |
| AL139022.1 | 0.062829 | 0.237158 | 1.916353 | 1.00E-12 | 4.22E-11 |
| OR3D1P | 0.024057 | 0.079351 | 1.721798 | 6.91E-05 | 0.000291 |
| AC100821.1 | 0.105021 | 0.212745 | 1.01845 | 4.59E-20 | 2.98E-17 |
| RF00003 | 0.212887 | 1.180522 | 2.471268 | 3.81E-12 | 1.33E-10 |
| RPL23AP90 | 0.101362 | 0.235648 | 1.21711 | 5.04E-09 | 7.14E-08 |
| AC105339.3 | 0.137592 | 0.302999 | 1.138918 | 5.46E-14 | 3.47E-12 |
| KCNMA1-AS1 | 0.014131 | 0.045822 | 1.69716 | 0.000468 | 0.001533 |
| AL162724.1 | 0.044465 | 0.20099 | 2.176369 | 0.000318 | 0.001097 |
| AC005746.1 | 0.054726 | 0.131885 | 1.268968 | 2.10E-14 | 1.49E-12 |
| SIDT1-AS1 | 0.061331 | 0.19908 | 1.698669 | 1.23E-06 | 8.63E-06 |
| NPIPB12 | 0.024606 | 0.05154 | 1.066713 | 7.45E-11 | 1.79E-09 |
| MRPS18AP1 | 0.427308 | 0.924804 | 1.113872 | 3.00E-09 | 4.53E-08 |
| AL132765.1 | 0.053481 | 0.27309 | 2.352265 | 2.83E-09 | 4.30E-08 |
| IL13 | 0.021832 | 0.056559 | 1.373304 | 0.000347 | 0.001183 |
| AP005131.6 | 0.08194 | 0.383577 | 2.226871 | 3.98E-05 | 0.00018 |
| AC006042.2 | 0.052034 | 0.152632 | 1.552531 | 0.010379 | 0.022091 |
| AC002347.2 | 0.080492 | 0.164651 | 1.032489 | 0.000327 | 0.001124 |
| PRSS37 | 0.014297 | 0.032334 | 1.177383 | 0.000339 | 0.001159 |
| AC244093.5 | 0.053187 | 0.158101 | 1.571695 | 7.58E-13 | 3.32E-11 |
| SMIM18 | 0.027361 | 0.074818 | 1.45124 | 0.016942 | 0.033428 |
| AC010735.1 | 0.058235 | 0.272182 | 2.224612 | 1.68E-07 | 1.54E-06 |
| AC008115.3 | 0.577934 | 1.259536 | 1.123916 | 8.85E-12 | 2.81E-10 |
| RN7SKP23 | 0.07525 | 0.157451 | 1.06513 | 7.57E-14 | 4.53E-12 |
| RN7SKP237 | 0.040907 | 0.198048 | 2.275418 | 0.001781 | 0.004878 |
| AC008280.1 | 0.04394 | 0.230829 | 2.393203 | 2.45E-05 | 0.000118 |
| AC009163.3 | 0.033043 | 0.111736 | 1.757668 | 2.72E-07 | 2.33E-06 |
| FAM205C | 0.003233 | 0.008819 | 1.447732 | 0.009676 | 0.020802 |
| AC083973.1 | 0.131368 | 0.303851 | 1.209748 | 3.14E-05 | 0.000147 |
| SALRNA2 | 0.005404 | 0.027597 | 2.352318 | 0.000382 | 0.001285 |
| RN7SL417P | 0.154834 | 0.325283 | 1.070976 | 8.21E-06 | 4.56E-05 |
| AL136146.1 | 0.031421 | 0.168386 | 2.421994 | 0.005042 | 0.011911 |
| AL022238.2 | 0.034519 | 0.171676 | 2.31421 | 2.26E-06 | 1.49E-05 |
| RERG-IT1 | 0.079114 | 0.207342 | 1.390007 | 1.72E-06 | 1.17E-05 |
| AC007878.1 | 0.17118 | 0.983199 | 2.521968 | 1.68E-17 | 3.80E-15 |
| CSNK1G2-AS1 | 0.016752 | 0.047832 | 1.513679 | 7.88E-12 | 2.54E-10 |
| ZNF730 | 0.060372 | 0.128862 | 1.093864 | 0.000463 | 0.00152 |
| RPL35AP32 | 0.073131 | 0.201455 | 1.461896 | 6.50E-07 | 4.97E-06 |
| RN7SL444P | 0.058923 | 0.130486 | 1.146991 | 0.007614 | 0.016945 |
| RPL7P21 | 0.067538 | 0.152753 | 1.177418 | 0.000578 | 0.001848 |
| ATF3 | 23.77575 | 50.55824 | 1.088455 | 3.59E-06 | 2.23E-05 |
| MIR1285-1 | 0.283492 | 0.764116 | 1.430482 | 1.94E-11 | 5.53E-10 |
| KPNA2P2 | 0.012592 | 0.034796 | 1.466385 | 0.003525 | 0.008776 |
| AC063965.1 | 0.116466 | 0.259381 | 1.155166 | 1.17E-08 | 1.50E-07 |
| IGHV1-58 | 0.579801 | 2.161268 | 1.898248 | 0.00731 | 0.016365 |
| ITCH-IT1 | 0.045419 | 0.128202 | 1.497049 | 0.000118 | 0.000466 |
| RNU6-759P | 0.145823 | 0.384996 | 1.400621 | 0.027267 | 0.049862 |
| XIRP1 | 0.272861 | 0.960739 | 1.815976 | 0.000144 | 0.000552 |
| AC073476.1 | 0.308656 | 0.666905 | 1.111482 | 4.96E-15 | 4.24E-13 |
| MAL2-AS1 | 0.045467 | 0.134952 | 1.569562 | 1.54E-10 | 3.41E-09 |
| Z97652.1 | 0.037223 | 0.095985 | 1.366628 | 2.86E-05 | 0.000135 |
| AC015914.1 | 0.049113 | 0.126023 | 1.359506 | 7.58E-08 | 7.70E-07 |
| AL391994.1 | 0.188608 | 0.515478 | 1.450516 | 1.35E-11 | 4.09E-10 |
| AC090607.1 | 0.089489 | 0.187816 | 1.069531 | 2.75E-12 | 1.02E-10 |
| AC107909.2 | 0.045052 | 0.133379 | 1.565853 | 6.52E-07 | 4.99E-06 |
| AC064801.2 | 0.034446 | 0.383602 | 3.477205 | 4.93E-16 | 6.39E-14 |
| HMGB1P51 | 0.016139 | 0.161822 | 3.325744 | 0.000126 | 0.000494 |
| TPI1P4 | 0.016795 | 0.067324 | 2.003121 | 1.67E-05 | 8.47E-05 |
| AL157938.3 | 0.02172 | 0.046833 | 1.108457 | 0.000612 | 0.001941 |
| AC027698.1 | 0.030872 | 0.158307 | 2.35837 | 5.06E-05 | 0.000222 |
| RNU6-1223P | 0.275233 | 0.553691 | 1.008427 | 3.17E-12 | 1.15E-10 |
| PPIAP91 | 0.09037 | 0.293474 | 1.699322 | 1.02E-09 | 1.77E-08 |
| AC099778.2 | 0.087145 | 0.215025 | 1.303011 | 1.24E-09 | 2.12E-08 |
| AC037198.1 | 0.438062 | 1.516866 | 1.791887 | 4.23E-07 | 3.41E-06 |
| AL590560.2 | 0.033727 | 0.10134 | 1.587244 | 0.000707 | 0.002199 |
| AL031595.1 | 0.007272 | 0.014903 | 1.035153 | 9.57E-05 | 0.000388 |
| MIR10B | 0.194013 | 0.43974 | 1.180493 | 4.40E-09 | 6.34E-08 |
| TSPYL6 | 0.003654 | 0.03794 | 3.376127 | 0.000224 | 0.00081 |
| AC008268.1 | 0.024358 | 0.066868 | 1.456937 | 0.017167 | 0.033806 |
| AC106037.2 | 0.039051 | 0.154361 | 1.982864 | 2.88E-06 | 1.84E-05 |
| IGHV4-39 | 8.938539 | 24.75174 | 1.469419 | 0.016714 | 0.033039 |
| AC016292.2 | 0.044782 | 0.090849 | 1.020552 | 6.20E-05 | 0.000264 |
| AC090950.1 | 0.034269 | 0.085625 | 1.321118 | 0.003225 | 0.008132 |
| HTR5BP | 0.013841 | 0.033818 | 1.288861 | 0.000121 | 0.000475 |
| AL354733.2 | 0.015609 | 0.049533 | 1.665967 | 4.20E-10 | 8.14E-09 |
| LINC01107 | 0.008004 | 0.017836 | 1.155911 | 2.04E-05 | 0.000101 |
| AC124319.1 | 0.147831 | 0.430824 | 1.543155 | 1.18E-10 | 2.68E-09 |
| AC022540.1 | 0.014994 | 0.048622 | 1.697256 | 0.000541 | 0.001744 |
| CAP2P1 | 0.035414 | 0.083121 | 1.230914 | 0.005479 | 0.012783 |
| AL136982.3 | 0.021444 | 0.205161 | 3.258083 | 9.05E-06 | 4.97E-05 |
| AC083843.2 | 0.715752 | 1.493327 | 1.060998 | 2.22E-20 | 1.80E-17 |
| MTND4P26 | 0.023052 | 0.071225 | 1.627494 | 2.29E-05 | 0.000111 |
| MIR27A | 0.700871 | 2.071676 | 1.563578 | 0.000313 | 0.001084 |
| AC027544.1 | 0.037746 | 0.113477 | 1.587995 | 8.79E-10 | 1.56E-08 |
| AC090236.2 | 0.309787 | 0.619828 | 1.00059 | 1.35E-05 | 7.02E-05 |
| AP000873.3 | 0.020845 | 0.063329 | 1.60319 | 1.03E-06 | 7.44E-06 |
| HSPA8P15 | 0.063839 | 0.18418 | 1.528608 | 1.56E-11 | 4.60E-10 |
| AC027544.2 | 0.038288 | 0.095492 | 1.318508 | 2.87E-08 | 3.28E-07 |
| SLC25A6P4 | 0.016081 | 0.035601 | 1.146598 | 1.04E-05 | 5.62E-05 |
| RN7SL834P | 0.322221 | 1.067234 | 1.727752 | 6.06E-14 | 3.78E-12 |
| CICP22 | 0.010868 | 0.024045 | 1.145639 | 1.59E-10 | 3.49E-09 |
| AC027130.1 | 0.026036 | 0.062341 | 1.259646 | 1.08E-06 | 7.74E-06 |
| AC009229.4 | 0.016437 | 0.052575 | 1.677422 | 0.006386 | 0.014566 |
| AP001931.1 | 0.278478 | 0.884598 | 1.667459 | 1.22E-19 | 6.10E-17 |
| RN7SL663P | 0.147261 | 0.308984 | 1.069158 | 1.65E-13 | 8.84E-12 |
| AC006539.2 | 0.061612 | 0.177561 | 1.527033 | 7.01E-13 | 3.10E-11 |
| AC010327.2 | 0.057994 | 0.116558 | 1.00709 | 1.31E-08 | 1.65E-07 |
| AC023509.5 | 0.021449 | 0.19704 | 3.199492 | 0.003719 | 0.009186 |
| PNMA5 | 0.029127 | 3.075237 | 6.722208 | 0.023545 | 0.044119 |
| GRPR | 1.144714 | 2.422056 | 1.081246 | 5.46E-05 | 0.000237 |
| AP000356.1 | 0.031385 | 0.086601 | 1.464321 | 0.000446 | 0.001471 |
| AC083806.2 | 0.039667 | 0.089117 | 1.167773 | 1.18E-13 | 6.61E-12 |
| LINC01695 | 0.046928 | 0.179518 | 1.935605 | 0.009061 | 0.019675 |
| AC010207.1 | 0.077079 | 0.15789 | 1.034521 | 3.76E-08 | 4.17E-07 |
| AC010768.2 | 0.042967 | 0.169272 | 1.97804 | 1.41E-07 | 1.32E-06 |
| RPL37P6 | 2.008564 | 0.952491 | -1.07639 | 2.03E-08 | 2.44E-07 |
| Z99289.2 | 0.01193 | 0.034266 | 1.52219 | 0.023791 | 0.044506 |
| AL591623.2 | 0.068379 | 0.21655 | 1.663065 | 3.77E-06 | 2.33E-05 |
| AC079035.1 | 0.033647 | 0.088746 | 1.399205 | 0.020299 | 0.038875 |
| AC011676.2 | 0.017983 | 0.074257 | 2.045851 | 1.06E-05 | 5.72E-05 |
| RN7SL239P | 0.095504 | 0.27373 | 1.519121 | 1.40E-05 | 7.26E-05 |
| AL139147.1 | 0.021111 | 0.105396 | 2.319761 | 0.004516 | 0.010845 |
| AP000866.3 | 0.276927 | 0.677637 | 1.291009 | 2.87E-12 | 1.06E-10 |
| COL4A2-AS2 | 0.014926 | 0.068057 | 2.188898 | 0.003994 | 0.009757 |
| MIR3188 | 0.101574 | 0.204521 | 1.009724 | 2.18E-05 | 0.000107 |
| IGKV1D-17 | 0.286509 | 0.913077 | 1.672155 | 0.014337 | 0.029014 |
| LRRC37A9P | 0.006889 | 0.01419 | 1.042561 | 1.63E-05 | 8.31E-05 |
| CICP10 | 0.044763 | 0.12664 | 1.500368 | 1.45E-11 | 4.36E-10 |
| AC079140.1 | 0.041757 | 0.109145 | 1.386153 | 1.12E-06 | 7.99E-06 |
| AC055811.4 | 0.482591 | 1.066699 | 1.14428 | 2.52E-22 | 7.73E-19 |
| C16orf54 | 1.03373 | 2.079638 | 1.008473 | 0.004732 | 0.011286 |
| MIR7851 | 0.070141 | 0.171901 | 1.293243 | 9.94E-07 | 7.22E-06 |
| RNU6-199P | 0.113919 | 0.2772 | 1.282913 | 0.000215 | 0.000781 |
| AC092718.6 | 0.047451 | 0.174304 | 1.877095 | 2.05E-08 | 2.45E-07 |
| AL031009.1 | 0.152645 | 0.377334 | 1.305665 | 6.57E-22 | 1.32E-18 |
| HNRNPA1P59 | 0.14936 | 0.444662 | 1.573919 | 9.70E-19 | 3.43E-16 |
| ICOS | 0.229578 | 0.56053 | 1.287807 | 1.17E-06 | 8.32E-06 |
| AC093627.3 | 0.02144 | 0.051666 | 1.268913 | 7.25E-07 | 5.48E-06 |
| SPIN2A | 0.020305 | 0.100857 | 2.312431 | 0.000193 | 0.000713 |
| GPR79 | 0.009387 | 0.036058 | 1.94155 | 3.78E-08 | 4.18E-07 |
| RPS27AP10 | 0.048152 | 0.370389 | 2.94338 | 5.22E-08 | 5.56E-07 |
| AC024267.5 | 0.406086 | 1.016677 | 1.324004 | 1.80E-13 | 9.57E-12 |
| MIR491 | 0.113249 | 0.27753 | 1.293148 | 4.97E-07 | 3.94E-06 |
| TNF | 0.181104 | 0.400636 | 1.145476 | 1.67E-05 | 8.50E-05 |
| AC100782.1 | 0.044004 | 0.152201 | 1.790287 | 2.40E-08 | 2.81E-07 |
| SMARCA5-AS1 | 0.018791 | 0.039976 | 1.0891 | 0.000446 | 0.001472 |
| AL138721.1 | 0.140348 | 0.606628 | 2.111802 | 5.01E-06 | 2.97E-05 |
| AC092168.2 | 0.064045 | 0.413094 | 2.689309 | 0.001866 | 0.005074 |
| AP001020.2 | 0.037245 | 0.089377 | 1.262841 | 9.09E-14 | 5.29E-12 |
| MIR4635 | 0.326155 | 0.70232 | 1.10657 | 4.09E-07 | 3.32E-06 |
| RF00272 | 0.040402 | 0.159406 | 1.980189 | 4.01E-11 | 1.04E-09 |
| RN7SL510P | 0.034302 | 0.077804 | 1.181566 | 3.03E-06 | 1.93E-05 |
| AC023906.5 | 0.178964 | 0.436304 | 1.285668 | 1.55E-11 | 4.57E-10 |
| GNRHR | 0.057416 | 0.121728 | 1.084136 | 0.000352 | 0.001197 |
| AC092802.2 | 0.108505 | 0.217321 | 1.002068 | 1.67E-11 | 4.86E-10 |
| LINC01594 | 0.30626 | 0.625269 | 1.02972 | 0.009103 | 0.019754 |
| MIR3174 | 0.108456 | 0.243008 | 1.163893 | 6.96E-05 | 0.000293 |
| AP001775.2 | 0.182066 | 0.444757 | 1.288552 | 3.34E-05 | 0.000155 |
| Z98048.1 | 0.029439 | 0.08775 | 1.575647 | 1.00E-06 | 7.27E-06 |
| AC115989.1 | 0.075703 | 0.180923 | 1.256951 | 1.80E-07 | 1.63E-06 |
| AC020612.3 | 0.050877 | 0.176681 | 1.796079 | 7.90E-05 | 0.000328 |
| AL121955.1 | 0.022758 | 0.158495 | 2.800012 | 2.33E-07 | 2.04E-06 |
| AC010642.1 | 0.029506 | 0.064003 | 1.117148 | 1.58E-09 | 2.61E-08 |
| RDM1P3 | 0.096647 | 0.280075 | 1.535019 | 6.27E-07 | 4.82E-06 |
| AP000560.1 | 0.100356 | 0.219527 | 1.129277 | 0.00011 | 0.000437 |
| AC099677.4 | 0.03891 | 0.082652 | 1.086902 | 3.80E-08 | 4.20E-07 |
| AC145207.8 | 0.124067 | 0.278188 | 1.164936 | 4.38E-16 | 5.73E-14 |
| AC011525.1 | 0.132059 | 0.39349 | 1.57514 | 2.52E-06 | 1.64E-05 |
| AC018695.4 | 0.148732 | 0.313199 | 1.074361 | 1.25E-17 | 2.97E-15 |
| HNRNPA3P11 | 0.032658 | 0.260476 | 2.995651 | 0.000127 | 0.000497 |
| AC092301.1 | 0.170578 | 0.346987 | 1.024452 | 6.00E-14 | 3.76E-12 |
| AL158827.2 | 0.135681 | 0.32455 | 1.258228 | 1.59E-07 | 1.46E-06 |
| AC022150.4 | 0.316724 | 0.659791 | 1.058782 | 2.31E-15 | 2.24E-13 |
| AC112512.1 | 0.021938 | 0.103769 | 2.241849 | 0.006935 | 0.015633 |
| AC005304.3 | 0.031606 | 0.122423 | 1.953618 | 0.0161 | 0.032022 |
| AC016683.1 | 0.02515 | 0.109683 | 2.124695 | 4.74E-07 | 3.78E-06 |
| ASH1L-IT1 | 0.017935 | 0.104308 | 2.539978 | 6.27E-09 | 8.67E-08 |
| AC008735.2 | 1.327445 | 2.87446 | 1.114639 | 1.55E-11 | 4.57E-10 |
| PTTG4P | 0.070235 | 0.409224 | 2.542635 | 3.68E-07 | 3.03E-06 |
| AL031577.1 | 0.297115 | 0.656921 | 1.144698 | 8.91E-11 | 2.09E-09 |
| CPA1 | 0.056179 | 0.027706 | -1.01981 | 0.007984 | 0.017639 |
| CYP3A4 | 0.038457 | 0.127194 | 1.725716 | 5.05E-07 | 3.99E-06 |
| AC036176.2 | 0.011079 | 0.03426 | 1.628679 | 0.00585 | 0.013514 |
| AC007787.1 | 0.013539 | 0.044308 | 1.710446 | 7.52E-05 | 0.000314 |
| AC010285.1 | 0.063762 | 0.163225 | 1.356091 | 2.50E-11 | 6.88E-10 |
| AC068790.7 | 0.178363 | 0.370793 | 1.055801 | 1.52E-05 | 7.81E-05 |
| AC107072.2 | 0.103955 | 0.302279 | 1.539925 | 3.32E-07 | 2.77E-06 |
| AC008033.3 | 0.01134 | 0.039783 | 1.810799 | 0.011003 | 0.023208 |
| AC107072.1 | 0.122219 | 0.254958 | 1.060792 | 0.003446 | 0.008609 |
| CICP16 | 0.039261 | 0.081577 | 1.055079 | 4.01E-16 | 5.34E-14 |
| AC099677.1 | 1.868734 | 5.927607 | 1.665389 | 7.30E-20 | 3.95E-17 |
| MYH7B | 0.329011 | 0.726133 | 1.142099 | 0.001096 | 0.00321 |
| CXCL2 | 4.536052 | 10.95973 | 1.272704 | 0.017864 | 0.034936 |
| C5orf58 | 0.02672 | 0.065582 | 1.295397 | 0.001525 | 0.004266 |
| AL365436.2 | 0.112193 | 0.227221 | 1.018114 | 1.51E-10 | 3.34E-09 |
| AC104964.1 | 0.018943 | 0.039677 | 1.066666 | 6.51E-05 | 0.000276 |
| AC141586.5 | 0.037165 | 0.1022 | 1.459386 | 5.40E-23 | 2.98E-19 |
| MTCO2P22 | 0.92474 | 0.268898 | -1.78199 | 4.61E-05 | 0.000205 |
| SNORD37 | 0.108555 | 0.270939 | 1.319541 | 3.10E-07 | 2.61E-06 |
| RF00322 | 0.016416 | 0.074732 | 2.186609 | 1.11E-08 | 1.43E-07 |
| AC048382.2 | 0.220732 | 0.460529 | 1.060995 | 8.39E-15 | 6.54E-13 |
| AC091230.1 | 1.054412 | 2.172398 | 1.04285 | 2.33E-05 | 0.000113 |
| LINC01593 | 0.072727 | 0.157991 | 1.11929 | 0.01411 | 0.028626 |
| AL034428.1 | 0.007753 | 0.018349 | 1.242759 | 3.33E-07 | 2.78E-06 |
| ZMYND19P1 | 0.027165 | 0.101584 | 1.902855 | 1.78E-09 | 2.88E-08 |
| RNA5SP151 | 0.096645 | 1.229768 | 3.669545 | 2.28E-07 | 2.00E-06 |
| AL022323.2 | 0.006425 | 0.065706 | 3.354277 | 0.000116 | 0.000458 |
| PCSK1 | 0.090851 | 0.466678 | 2.360857 | 0.011461 | 0.024016 |
| AL645924.2 | 0.051277 | 0.128107 | 1.320971 | 2.53E-07 | 2.19E-06 |
| RF00432 | 0.034385 | 0.084287 | 1.293515 | 5.00E-05 | 0.000219 |
| RNA5SP82 | 0.410765 | 0.995873 | 1.277648 | 5.95E-12 | 1.97E-10 |
| AL590627.1 | 0.064383 | 0.169965 | 1.400478 | 5.13E-18 | 1.43E-15 |
| AC006058.1 | 0.021038 | 0.046847 | 1.154948 | 0.002524 | 0.006563 |
| RNU7-45P | 0.236063 | 0.516006 | 1.128214 | 5.46E-06 | 3.22E-05 |
| SNORA22 | 0.211908 | 6.237002 | 4.879346 | 4.32E-14 | 2.81E-12 |
| AL445685.1 | 0.024998 | 0.110591 | 2.145377 | 0.000762 | 0.002349 |
| SLCO1B1 | 0.028799 | 0.092081 | 1.676894 | 0.026563 | 0.048756 |
| FAM71C | 0.004702 | 0.028971 | 2.623154 | 7.04E-08 | 7.24E-07 |
| AC006946.1 | 0.058674 | 0.176621 | 1.589868 | 3.49E-10 | 6.96E-09 |
| MIR6753 | 0.144067 | 0.300398 | 1.060135 | 1.40E-06 | 9.69E-06 |
| AC116366.2 | 0.014611 | 0.144515 | 3.306102 | 1.99E-05 | 9.87E-05 |
| AC104819.1 | 0.035114 | 0.70601 | 4.32959 | 0.013445 | 0.027515 |
| AOAH-IT1 | 0.009627 | 0.077957 | 3.01757 | 2.88E-09 | 4.37E-08 |
| AC012676.5 | 0.254977 | 0.549974 | 1.108997 | 6.35E-20 | 3.53E-17 |
| AC112496.1 | 0.05945 | 0.196123 | 1.722013 | 1.66E-10 | 3.63E-09 |
| TXNDC12-AS1 | 0.06934 | 0.169522 | 1.289706 | 0.001599 | 0.004442 |
| CMPK2 | 1.17887 | 2.500953 | 1.085073 | 2.37E-06 | 1.56E-05 |
| AC092042.1 | 0.029911 | 0.08056 | 1.429385 | 2.56E-07 | 2.22E-06 |
| LINC02325 | 0.01537 | 0.030904 | 1.007666 | 0.010107 | 0.021587 |
| AP003072.3 | 0.086476 | 0.189891 | 1.134805 | 9.05E-08 | 9.01E-07 |
| RNU1-120P | 0.118716 | 0.46939 | 1.98327 | 0.007066 | 0.015886 |
| BOLL | 0.009169 | 0.019832 | 1.112996 | 1.58E-05 | 8.07E-05 |
| IGKV1-27 | 2.922723 | 7.903874 | 1.435247 | 0.010789 | 0.022823 |
| AC087222.1 | 0.173834 | 0.424556 | 1.288249 | 2.51E-13 | 1.27E-11 |
| LINC02421 | 0.010008 | 0.033082 | 1.724921 | 4.01E-09 | 5.82E-08 |
| AC008737.1 | 0.1624 | 0.395318 | 1.28346 | 1.10E-10 | 2.53E-09 |
| AL138689.1 | 0.421135 | 0.902365 | 1.099429 | 0.006317 | 0.014429 |
| AC020765.3 | 0.017741 | 0.039035 | 1.137722 | 5.46E-05 | 0.000237 |
| AC146507.2 | 0.054521 | 0.134795 | 1.305873 | 0.000741 | 0.002294 |
| ANKUB1 | 0.006921 | 0.01504 | 1.119762 | 0.000173 | 0.000649 |
| AC018445.3 | 0.004646 | 0.034705 | 2.901131 | 0.000151 | 0.000576 |
| RAI1-AS1 | 0.039679 | 0.137104 | 1.788808 | 0.010166 | 0.021701 |
| CELF2-AS1 | 0.025137 | 0.119943 | 2.254456 | 6.84E-09 | 9.34E-08 |
| AC025271.2 | 0.052451 | 0.220706 | 2.073083 | 1.22E-13 | 6.79E-12 |
| AL450263.1 | 0.262089 | 0.587976 | 1.165701 | 3.19E-20 | 2.43E-17 |
| DDN | 0.216819 | 0.604568 | 1.479412 | 0.005265 | 0.01236 |
| Z99129.1 | 0.105385 | 0.257859 | 1.290913 | 2.08E-09 | 3.28E-08 |
| AC124276.2 | 0.025102 | 0.087838 | 1.807064 | 3.40E-05 | 0.000158 |
| C6orf223 | 0.008209 | 0.031461 | 1.938358 | 0.001204 | 0.003489 |
| AC073655.1 | 0.045597 | 0.094989 | 1.058808 | 0.005823 | 0.013464 |
| SLC7A11-AS1 | 0.016852 | 0.060203 | 1.836877 | 5.23E-08 | 5.57E-07 |
| RYKP1 | 0.022872 | 0.084433 | 1.884204 | 3.05E-07 | 2.57E-06 |
| RN7SKP163 | 0.025457 | 0.191563 | 2.911709 | 2.12E-07 | 1.87E-06 |
| AL136310.1 | 0.032522 | 0.08324 | 1.355867 | 1.02E-05 | 5.51E-05 |
| MKRN5P | 0.018895 | 0.051079 | 1.434763 | 0.006765 | 0.015298 |
| RF00554 | 0.01837 | 0.061862 | 1.751696 | 4.50E-06 | 2.71E-05 |
| AC067945.3 | 0.062308 | 0.147406 | 1.242309 | 2.69E-06 | 1.74E-05 |
| PRDX3P2 | 0.071887 | 0.19904 | 1.469246 | 4.26E-07 | 3.43E-06 |
| LINC00102 | 0.011563 | 0.066765 | 2.529576 | 9.28E-06 | 5.08E-05 |
| DUSP5P1 | 0.0914 | 0.209217 | 1.194727 | 5.61E-05 | 0.000243 |
| RN7SL49P | 0.069343 | 0.148083 | 1.094577 | 0.000686 | 0.002146 |
| AL590438.1 | 0.003643 | 0.009294 | 1.351068 | 0.010543 | 0.022387 |
| PRDX2P3 | 0.035707 | 0.093056 | 1.381891 | 3.11E-08 | 3.52E-07 |
| LINC01359 | 0.08795 | 0.249147 | 1.502239 | 2.41E-09 | 3.73E-08 |
| SH2D1A | 0.783978 | 2.531418 | 1.691061 | 0.004749 | 0.011325 |
| AC087257.2 | 0.028255 | 0.105644 | 1.902631 | 0.004534 | 0.010881 |
| AC010533.1 | 0.008936 | 0.023738 | 1.409468 | 0.00027 | 0.000952 |
| P2RY10 | 0.378435 | 0.805 | 1.088944 | 9.73E-05 | 0.000393 |
| MIR1972-1 | 0.17547 | 0.453727 | 1.370604 | 0.000152 | 0.00058 |
| SERPINA3 | 0.754553 | 1.818335 | 1.268924 | 0.001592 | 0.004427 |
| MTND5P1 | 0.051206 | 0.108889 | 1.088474 | 6.66E-10 | 1.22E-08 |
| AC004696.2 | 0.122317 | 0.324907 | 1.409408 | 1.83E-10 | 3.96E-09 |
| AC109597.2 | 0.023561 | 0.06737 | 1.515704 | 0.0007 | 0.00218 |
| AC132872.4 | 0.126228 | 0.312451 | 1.307597 | 4.15E-21 | 5.45E-18 |
| AC111186.1 | 0.093285 | 0.201707 | 1.112536 | 1.49E-05 | 7.65E-05 |
| RPS3P2 | 0.038571 | 0.143672 | 1.897178 | 6.39E-07 | 4.90E-06 |
| AC211476.2 | 0.119004 | 0.355754 | 1.579876 | 4.05E-05 | 0.000183 |
| AC079336.7 | 0.020679 | 0.05455 | 1.399414 | 3.24E-06 | 2.04E-05 |
| LINC01698 | 0.305587 | 0.098515 | -1.63317 | 0.003282 | 0.008258 |
| AC008735.1 | 0.141696 | 0.294818 | 1.057021 | 7.37E-13 | 3.25E-11 |
| RF00072 | 0.020056 | 0.099925 | 2.316835 | 0.000351 | 0.001194 |
| Z98752.2 | 0.03096 | 0.164916 | 2.413274 | 1.37E-06 | 9.55E-06 |
| AC092145.1 | 0.012244 | 0.043863 | 1.8409 | 4.63E-05 | 0.000205 |
| CYCSP52 | 0.041589 | 0.138347 | 1.734029 | 5.13E-09 | 7.25E-08 |
| SCARNA8 | 0.123266 | 2.052726 | 4.057694 | 7.01E-10 | 1.28E-08 |
| AC012186.3 | 0.014246 | 0.093967 | 2.721615 | 5.60E-07 | 4.37E-06 |
| PPP1R16B | 0.647333 | 1.306038 | 1.012617 | 4.12E-08 | 4.52E-07 |
| AC110760.2 | 0.017947 | 0.148708 | 3.050651 | 0.008587 | 0.018784 |
| IFITM5 | 0.019712 | 0.065099 | 1.723571 | 6.95E-08 | 7.15E-07 |
| RN7SL650P | 0.04178 | 0.122443 | 1.551224 | 4.34E-12 | 1.50E-10 |
| AL356801.1 | 0.333277 | 0.958512 | 1.524076 | 6.81E-16 | 8.38E-14 |
| AC005034.2 | 0.086024 | 0.268108 | 1.640008 | 3.08E-05 | 0.000144 |
| H2AFZP1 | 0.029161 | 0.160948 | 2.464469 | 1.83E-05 | 9.18E-05 |
| ODF2-AS1 | 0.111746 | 0.286038 | 1.355983 | 2.21E-10 | 4.68E-09 |
| AC027514.1 | 0.030918 | 0.073756 | 1.254306 | 0.015373 | 0.030839 |
| AC109631.1 | 0.017966 | 0.040615 | 1.176717 | 7.65E-07 | 5.73E-06 |
| AC009464.1 | 0.052368 | 0.135017 | 1.366383 | 5.05E-05 | 0.000221 |
| HNRNPA3P9 | 0.12152 | 0.394346 | 1.698267 | 3.37E-15 | 3.07E-13 |
| AL031670.1 | 0.275931 | 0.554126 | 1.005904 | 5.97E-17 | 1.11E-14 |
| AC098850.3 | 0.023556 | 0.050751 | 1.107321 | 2.02E-05 | 9.99E-05 |
| RNU6-863P | 0.136669 | 0.417233 | 1.610167 | 7.12E-09 | 9.66E-08 |
| PBOV1 | 0.033822 | 0.260366 | 2.944487 | 1.18E-09 | 2.02E-08 |
| AC027796.2 | 0.102222 | 0.239267 | 1.226919 | 1.30E-13 | 7.17E-12 |
| AC008507.2 | 0.032494 | 0.103743 | 1.674783 | 3.66E-09 | 5.40E-08 |
| AC055764.1 | 0.023626 | 0.084283 | 1.834856 | 0.004294 | 0.010385 |
| LINC00299 | 0.018407 | 0.042045 | 1.191641 | 6.17E-07 | 4.76E-06 |
| RNU4-5P | 0.061727 | 0.198519 | 1.685306 | 3.83E-08 | 4.22E-07 |
| LINC00923 | 0.026256 | 0.056668 | 1.109872 | 0.02065 | 0.039445 |
| AC131280.1 | 0.048886 | 0.139558 | 1.513376 | 0.002922 | 0.007463 |
| AP002373.1 | 0.020268 | 0.045537 | 1.16788 | 7.46E-05 | 0.000311 |
| AC032011.1 | 0.068945 | 0.138699 | 1.008445 | 0.000905 | 0.002726 |
| AC021171.1 | 0.015311 | 0.032959 | 1.106099 | 0.000101 | 0.000407 |
| AL109761.1 | 0.043182 | 0.13234 | 1.615739 | 3.49E-08 | 3.90E-07 |
| AL512306.3 | 0.111207 | 0.319482 | 1.522485 | 1.69E-18 | 5.61E-16 |
| GPR31 | 0.016074 | 0.035483 | 1.142448 | 1.41E-05 | 7.29E-05 |
| WWTR1-IT1 | 0.071102 | 0.282433 | 1.989946 | 0.004399 | 0.0106 |
| MIAT | 0.142262 | 0.382424 | 1.426618 | 1.25E-09 | 2.13E-08 |
| MIR29B2CHG | 0.436241 | 1.213003 | 1.475386 | 8.11E-15 | 6.37E-13 |
| AC107081.3 | 0.00571 | 0.027444 | 2.264926 | 0.000424 | 0.001406 |
| AC008739.5 | 0.013199 | 0.040783 | 1.62751 | 0.00317 | 0.008006 |
| AC099811.1 | 0.036696 | 0.164691 | 2.166085 | 7.88E-08 | 7.98E-07 |
| AC010894.3 | 0.046873 | 0.163462 | 1.80211 | 0.002024 | 0.005439 |
| AC007923.4 | 0.020702 | 0.055941 | 1.434154 | 9.63E-06 | 5.25E-05 |
| AC087893.1 | 0.017011 | 0.157032 | 3.206544 | 0.003146 | 0.007953 |
| AC026470.2 | 0.074024 | 0.292362 | 1.9817 | 1.69E-06 | 1.15E-05 |
| AC090241.2 | 0.003075 | 0.008335 | 1.438779 | 0.012158 | 0.025253 |
| RF00334 | 0.063698 | 0.233393 | 1.873444 | 2.00E-06 | 1.34E-05 |
| RN7SL75P | 0.076084 | 0.164858 | 1.11555 | 2.13E-08 | 2.53E-07 |
| AL136084.2 | 0.020678 | 0.041628 | 1.009443 | 0.001702 | 0.004691 |
| AC022126.1 | 0.049186 | 0.118261 | 1.265662 | 0.001006 | 0.002984 |
| KDM5C-IT1 | 0.014043 | 0.03916 | 1.47954 | 9.62E-05 | 0.000389 |
| HLCS-IT1 | 0.052201 | 0.179301 | 1.780244 | 0.000554 | 0.001781 |
| AL591623.1 | 0.012746 | 0.075164 | 2.559978 | 0.016347 | 0.032434 |
| RN7SKP75 | 0.040572 | 0.09197 | 1.180693 | 0.025525 | 0.047181 |
| AC015961.1 | 0.02289 | 0.06026 | 1.39649 | 6.09E-11 | 1.51E-09 |
| AC127070.4 | 0.070313 | 0.176739 | 1.329754 | 8.65E-15 | 6.70E-13 |
| RNU6-126P | 0.087726 | 0.316077 | 1.849192 | 2.23E-07 | 1.96E-06 |
| SNAP47-AS1 | 0.020034 | 0.049547 | 1.306376 | 9.37E-06 | 5.12E-05 |
| AC002558.2 | 0.015703 | 0.042488 | 1.435998 | 0.002791 | 0.007171 |
| SNORD101 | 1.324943 | 2.721095 | 1.038257 | 8.29E-14 | 4.92E-12 |
| AL031587.1 | 0.013415 | 0.029809 | 1.151936 | 2.65E-05 | 0.000127 |
| OFD1P17 | 0.012323 | 0.033635 | 1.448659 | 7.85E-07 | 5.86E-06 |
| AC113146.1 | 0.01344 | 0.056781 | 2.078849 | 0.000951 | 0.002844 |
| CELP | 0.007023 | 0.016749 | 1.253904 | 0.000148 | 0.000566 |
| ZNF33BP1 | 0.035246 | 0.103576 | 1.55516 | 4.72E-08 | 5.10E-07 |
| AC009269.2 | 0.028161 | 0.074172 | 1.39719 | 0.012883 | 0.026543 |
| PDZRN3-AS1 | 0.02769 | 0.082307 | 1.571624 | 0.023573 | 0.044162 |
| ANKRD44-IT1 | 0.013582 | 0.329568 | 4.600803 | 0.000238 | 0.000852 |
| RNA5SP78 | 0.205335 | 0.507682 | 1.305949 | 1.54E-09 | 2.55E-08 |
| AL356273.1 | 0.006941 | 0.048091 | 2.79247 | 8.23E-06 | 4.57E-05 |
| ZBTB40-IT1 | 0.174859 | 0.522363 | 1.578859 | 1.79E-17 | 4.01E-15 |
| AC016405.1 | 0.057861 | 0.445502 | 2.944758 | 7.00E-07 | 5.30E-06 |
| AL078645.2 | 0.011384 | 0.04253 | 1.901403 | 3.73E-05 | 0.00017 |
| AC023906.4 | 0.027145 | 0.072333 | 1.413982 | 1.78E-09 | 2.89E-08 |
| ZNF630-AS1 | 0.037385 | 0.114414 | 1.613731 | 0.000144 | 0.000553 |
| AC005264.1 | 0.01461 | 0.053811 | 1.880897 | 2.01E-08 | 2.42E-07 |
| AL645608.8 | 0.057293 | 0.135476 | 1.241611 | 1.78E-06 | 1.21E-05 |
| AC008080.1 | 0.024448 | 0.06535 | 1.41846 | 0.00222 | 0.005893 |
| PGAM1P7 | 0.080227 | 0.185254 | 1.207353 | 2.34E-12 | 8.80E-11 |
| AP002373.2 | 0.036266 | 0.105443 | 1.539771 | 0.000118 | 0.000464 |
| AL358115.1 | 0.134512 | 0.326485 | 1.279284 | 2.41E-15 | 2.31E-13 |
| AC120057.4 | 0.226583 | 0.715935 | 1.659789 | 0.006186 | 0.014174 |
| RF00154 | 0.566078 | 2.300822 | 2.023076 | 1.72E-19 | 8.18E-17 |
| AC092436.4 | 0.021823 | 0.117166 | 2.424637 | 9.73E-06 | 5.30E-05 |
| AC025031.5 | 0.018302 | 0.078263 | 2.096368 | 0.00502 | 0.01187 |
| AL355388.1 | 0.037145 | 0.074833 | 1.01049 | 1.87E-05 | 9.35E-05 |
| ARL4AP4 | 0.076426 | 0.64018 | 3.066348 | 1.60E-16 | 2.52E-14 |
| TUBB1 | 0.031175 | 0.063571 | 1.02798 | 6.65E-12 | 2.18E-10 |
| RNVU1-3 | 0.658901 | 1.546134 | 1.230533 | 9.72E-09 | 1.28E-07 |
| YWHAQP6 | 0.058462 | 0.197346 | 1.755156 | 3.57E-07 | 2.96E-06 |
| AC010307.2 | 0.020216 | 0.046828 | 1.211903 | 0.023505 | 0.044062 |
| AC132068.1 | 0.021886 | 0.186991 | 3.094892 | 0.00077 | 0.002369 |
| AC010536.2 | 0.119436 | 0.414074 | 1.793651 | 3.93E-05 | 0.000178 |
| FP325331.1 | 0.030269 | 0.061028 | 1.011617 | 0.011575 | 0.02422 |
| AL513366.1 | 0.025315 | 0.066508 | 1.393532 | 2.57E-08 | 2.97E-07 |
| PRR13P2 | 0.0303 | 0.078885 | 1.380443 | 0.014128 | 0.028659 |
| ENPP7P2 | 0.045411 | 0.100437 | 1.145187 | 0.001102 | 0.003226 |
| CFLAR-AS1 | 0.05325 | 0.260374 | 2.28973 | 2.61E-18 | 8.07E-16 |
| AC020910.3 | 0.036545 | 0.149234 | 2.02981 | 1.40E-07 | 1.31E-06 |
| AF131215.2 | 0.014206 | 0.442315 | 4.960454 | 3.04E-05 | 0.000143 |
| ST7-OT4 | 0.035281 | 0.105272 | 1.577148 | 0.000499 | 0.001627 |
| AC016526.3 | 0.003817 | 0.0077 | 1.012391 | 2.58E-06 | 1.68E-05 |
| FCF1P5 | 0.028589 | 0.220833 | 2.949428 | 0.000193 | 0.000714 |
| ERI3-IT1 | 0.062245 | 0.246791 | 1.987268 | 1.87E-11 | 5.37E-10 |
| WASF5P | 0.083365 | 0.169936 | 1.027473 | 0.000669 | 0.002097 |
| FKBP1AP1 | 0.119951 | 0.245168 | 1.031323 | 3.69E-06 | 2.29E-05 |
| MIR6739 | 0.298053 | 0.632658 | 1.085856 | 2.13E-10 | 4.52E-09 |
| AL356310.1 | 0.042487 | 0.021041 | -1.01386 | 0.005126 | 0.012073 |
| TSPO2 | 0.027323 | 0.071153 | 1.380836 | 8.43E-08 | 8.46E-07 |
| PVRIG | 0.052058 | 0.11816 | 1.182548 | 1.62E-12 | 6.40E-11 |
| PWAR1 | 0.052617 | 0.126508 | 1.26564 | 1.33E-05 | 6.94E-05 |
| LINC00308 | 0.130979 | 0.656741 | 2.325984 | 0.000176 | 0.000657 |
| ENPP7P4 | 0.099665 | 0.227302 | 1.189455 | 3.42E-05 | 0.000158 |
| ZNF483 | 0.261502 | 0.526615 | 1.009928 | 2.98E-05 | 0.00014 |
| RN7SL674P | 0.444664 | 1.866023 | 2.069178 | 7.82E-07 | 5.84E-06 |
| RNU1-100P | 0.051306 | 0.553807 | 3.432178 | 0.000196 | 0.000722 |
| AP002505.2 | 0.031079 | 0.207814 | 2.741298 | 0.013005 | 0.026762 |
| CASC19 | 0.067948 | 0.246167 | 1.857126 | 0.019366 | 0.037388 |
| DPYD-AS1 | 0.009458 | 0.049526 | 2.388603 | 0.002005 | 0.005397 |
| AC092881.1 | 0.028056 | 0.161538 | 2.525484 | 3.39E-10 | 6.76E-09 |
| Z84478.1 | 0.009315 | 0.078216 | 3.069825 | 0.000222 | 0.000802 |
| AC079416.1 | 0.034898 | 0.099029 | 1.504711 | 8.85E-07 | 6.52E-06 |
| ASTN2-AS1 | 0.013894 | 0.134344 | 3.273379 | 0.023999 | 0.044832 |
| ENPP7P10 | 0.091917 | 0.235314 | 1.356188 | 0.001185 | 0.003438 |
| AC008080.4 | 0.012758 | 0.039964 | 1.647257 | 0.000227 | 0.000818 |
| MIR4324 | 0.172514 | 0.441105 | 1.354407 | 0.019486 | 0.037578 |
| AC010978.1 | 0.017983 | 0.074488 | 2.050388 | 0.009964 | 0.021333 |
| SLC22A25 | 0.005585 | 0.020487 | 1.875208 | 3.56E-06 | 2.22E-05 |
| AC011247.2 | 0.273223 | 0.683078 | 1.321972 | 0.001942 | 0.005249 |
| AC005899.1 | 0.051289 | 0.103387 | 1.01134 | 6.72E-06 | 3.84E-05 |
| AC098848.1 | 0.067207 | 0.153522 | 1.19176 | 1.18E-05 | 6.28E-05 |
| KLB | 0.395451 | 1.968041 | 2.315188 | 0.007109 | 0.01597 |
| SNORD117 | 0.415321 | 1.494221 | 1.847094 | 1.42E-13 | 7.79E-12 |
| TAS2R3 | 0.013587 | 0.06751 | 2.312839 | 0.000116 | 0.000459 |
| TTLL7-IT1 | 0.018305 | 0.048842 | 1.415911 | 0.002399 | 0.006295 |
| AC007278.1 | 0.022046 | 0.137546 | 2.641333 | 6.30E-07 | 4.84E-06 |
| AL391058.1 | 0.155665 | 0.353768 | 1.18436 | 0.006023 | 0.013846 |
| AC091390.1 | 0.091102 | 0.208618 | 1.195301 | 6.36E-09 | 8.76E-08 |
| AC018529.2 | 0.112901 | 0.341175 | 1.595448 | 1.57E-12 | 6.24E-11 |
| AC078795.1 | 0.050412 | 0.116288 | 1.205856 | 6.00E-16 | 7.49E-14 |
| AC004975.1 | 0.163245 | 0.495316 | 1.601311 | 3.31E-15 | 3.03E-13 |
| MIR2052HG | 0.427568 | 0.960574 | 1.167743 | 1.47E-06 | 1.01E-05 |
| AC015849.4 | 0.051491 | 0.138712 | 1.429706 | 0.000765 | 0.002355 |
| IZUMO1 | 0.063599 | 0.142496 | 1.163839 | 1.23E-07 | 1.17E-06 |
| AC073487.1 | 0.403546 | 0.847683 | 1.070794 | 3.28E-12 | 1.17E-10 |
| AC066613.1 | 0.20458 | 0.427316 | 1.062637 | 1.74E-16 | 2.67E-14 |
| RF00191 | 0.020377 | 0.139486 | 2.77514 | 8.56E-06 | 4.73E-05 |
| AC015727.1 | 0.100047 | 0.303997 | 1.603375 | 1.70E-09 | 2.78E-08 |
| AC007906.1 | 0.064891 | 0.190733 | 1.555464 | 4.49E-08 | 4.88E-07 |
| SNORD9 | 0.192862 | 0.471152 | 1.288622 | 3.17E-05 | 0.000148 |
| RN7SKP271 | 0.072771 | 0.229385 | 1.656342 | 6.14E-07 | 4.74E-06 |
| AL121652.1 | 0.075677 | 0.182757 | 1.272006 | 0.009447 | 0.020378 |
| AL391863.1 | 0.010822 | 0.143423 | 3.728276 | 7.89E-05 | 0.000328 |
| AC002064.2 | 1.352939 | 9.200225 | 2.765572 | 2.77E-13 | 1.38E-11 |
| RNU7-75P | 0.183789 | 0.850738 | 2.210666 | 1.90E-06 | 1.28E-05 |
| MIR3153 | 0.211964 | 1.091298 | 2.364156 | 4.65E-13 | 2.19E-11 |
| MIR579 | 0.13815 | 0.324849 | 1.233531 | 0.000243 | 0.000867 |
| MMP3 | 0.184594 | 0.475124 | 1.363948 | 0.003158 | 0.007978 |
| CD80 | 0.066615 | 0.149846 | 1.169557 | 2.84E-08 | 3.25E-07 |
| RN7SL704P | 0.048723 | 0.120818 | 1.310178 | 1.08E-06 | 7.76E-06 |
| AC020656.2 | 0.123253 | 0.321822 | 1.384636 | 0.009889 | 0.021207 |
| AC092338.1 | 0.08975 | 0.233762 | 1.38105 | 6.58E-11 | 1.61E-09 |
| AC114781.1 | 0.013425 | 0.03036 | 1.177232 | 0.000915 | 0.002751 |
| IGLV1-44 | 9.671187 | 19.36404 | 1.001615 | 0.025315 | 0.046856 |
| AC010320.4 | 0.041149 | 0.099142 | 1.268645 | 9.85E-07 | 7.16E-06 |
| RHOA-IT1 | 0.128842 | 0.285085 | 1.145789 | 4.22E-15 | 3.66E-13 |
| RPS2P36 | 0.012945 | 0.026681 | 1.043435 | 0.000215 | 0.000784 |
| SPDYE5 | 0.083108 | 0.169065 | 1.024525 | 8.04E-15 | 6.35E-13 |
| RNU6ATAC24P | 0.178959 | 0.64976 | 1.860278 | 4.20E-10 | 8.14E-09 |
| AP001020.3 | 0.035798 | 0.113258 | 1.661655 | 1.13E-15 | 1.27E-13 |
| NARF-IT1 | 0.232393 | 0.513166 | 1.142856 | 1.11E-15 | 1.26E-13 |
| AC007496.3 | 0.008139 | 0.075733 | 3.217931 | 0.008203 | 0.018045 |
| EDDM13 | 0.125285 | 0.287352 | 1.197611 | 6.43E-05 | 0.000273 |
| AP000577.1 | 0.130467 | 0.273103 | 1.065761 | 1.72E-06 | 1.17E-05 |
| PHF2P2 | 0.008847 | 0.019887 | 1.168496 | 0.000305 | 0.001059 |
| AC131953.1 | 0.055368 | 0.121434 | 1.133045 | 8.21E-11 | 1.95E-09 |
| SLC5A9 | 0.081007 | 0.169893 | 1.068509 | 0.000235 | 0.000845 |
| CCDC196 | 0.023664 | 0.052487 | 1.149256 | 0.000118 | 0.000463 |
| AC015853.3 | 0.036851 | 0.093802 | 1.347923 | 2.08E-08 | 2.48E-07 |
| AL645933.1 | 0.011335 | 0.025393 | 1.163657 | 2.72E-07 | 2.33E-06 |
| FOSB | 30.11754 | 74.36596 | 1.304039 | 3.64E-05 | 0.000167 |
| AL161773.1 | 0.019123 | 0.316587 | 4.049198 | 0.001028 | 0.003037 |
| AC027644.2 | 0.186826 | 0.541802 | 1.53607 | 8.47E-09 | 1.13E-07 |
| RNA5SP118 | 0.321815 | 0.832529 | 1.371271 | 7.29E-08 | 7.47E-07 |
| AC007610.4 | 0.028902 | 0.067592 | 1.225714 | 1.22E-16 | 2.01E-14 |
| AL731556.2 | 0.017416 | 0.040241 | 1.208284 | 0.001051 | 0.003096 |
| CLNK | 0.205962 | 0.422694 | 1.037235 | 0.00013 | 0.000506 |
| AC135050.5 | 0.548767 | 1.195947 | 1.123888 | 4.96E-21 | 6.21E-18 |
| AL451070.1 | 0.038608 | 0.079723 | 1.046081 | 0.000248 | 0.000883 |
| SINHCAFP1 | 0.044969 | 0.104767 | 1.220171 | 5.17E-05 | 0.000226 |
| RN7SL614P | 0.063991 | 0.136613 | 1.094162 | 7.97E-09 | 1.07E-07 |
| APOA1-AS | 0.071439 | 0.175183 | 1.294071 | 9.55E-07 | 6.97E-06 |
| AL590617.1 | 0.01381 | 0.032039 | 1.214122 | 0.001187 | 0.003443 |
| NF1P11 | 0.023131 | 0.058616 | 1.341445 | 0.026189 | 0.04818 |
| ARMC2-AS1 | 0.107001 | 0.380776 | 1.831325 | 0.000274 | 0.000964 |
| AC087752.2 | 0.142825 | 0.285952 | 1.001528 | 1.45E-07 | 1.35E-06 |
| AC111170.1 | 0.013992 | 0.116285 | 3.054965 | 4.71E-06 | 2.82E-05 |
| AC245884.9 | 0.055717 | 0.114113 | 1.034265 | 8.71E-07 | 6.42E-06 |
| AC025271.1 | 0.036934 | 0.115418 | 1.643842 | 0.000678 | 0.002123 |
| AC018638.8 | 1.565995 | 3.185954 | 1.024646 | 2.16E-18 | 6.77E-16 |
| AC022613.3 | 0.053522 | 0.171422 | 1.67936 | 5.14E-11 | 1.29E-09 |
| LINC01250 | 0.006643 | 0.013993 | 1.074669 | 0.002775 | 0.007136 |
| AL138847.2 | 0.039451 | 0.118637 | 1.588416 | 2.10E-07 | 1.86E-06 |
| AC107983.1 | 7.293669 | 3.60241 | -1.01768 | 8.93E-13 | 3.83E-11 |
| MIR5581 | 1.03439 | 2.654981 | 1.359922 | 2.21E-15 | 2.17E-13 |
| AC092953.1 | 0.044249 | 0.109323 | 1.304888 | 0.003327 | 0.008352 |
| AP001646.1 | 0.012413 | 0.034504 | 1.4749 | 0.006167 | 0.014135 |
| FAM30A | 0.050064 | 0.106276 | 1.085964 | 0.025656 | 0.047391 |
| AC093010.2 | 0.005879 | 0.013572 | 1.206908 | 0.005772 | 0.01336 |
| LINC00861 | 0.136171 | 0.278521 | 1.032372 | 0.009658 | 0.020768 |
| AC104827.1 | 0.015816 | 0.070571 | 2.157671 | 0.003746 | 0.00924 |
| HSD17B3-AS1 | 0.024357 | 0.0499 | 1.034697 | 1.83E-05 | 9.17E-05 |
| AP000266.1 | 0.056187 | 0.155537 | 1.468954 | 0.00075 | 0.002319 |
| AC025423.1 | 0.034209 | 0.074226 | 1.117538 | 2.92E-05 | 0.000138 |
| AC090589.2 | 0.022603 | 0.078669 | 1.799256 | 0.00043 | 0.001424 |
| AL589990.1 | 0.170358 | 0.34954 | 1.036889 | 4.12E-07 | 3.34E-06 |
| MX2 | 0.535585 | 1.216097 | 1.183069 | 9.21E-07 | 6.75E-06 |
| BNIP3P25 | 0.036704 | 0.221207 | 2.591367 | 0.006727 | 0.01522 |
| AC124312.6 | 0.065955 | 0.272436 | 2.046353 | 1.03E-12 | 4.31E-11 |
| SCOCP1 | 0.085122 | 0.187436 | 1.138788 | 0.000122 | 0.000478 |
| LRRTM3 | 0.084027 | 0.040387 | -1.05697 | 0.008842 | 0.01926 |
| AC055748.1 | 0.022044 | 0.060867 | 1.465295 | 9.97E-09 | 1.31E-07 |
| BNIP3P42 | 0.037478 | 0.088847 | 1.245305 | 0.000172 | 0.000646 |
| AL133230.1 | 0.074091 | 0.263965 | 1.832979 | 5.57E-20 | 3.34E-17 |
| AL050341.1 | 0.120173 | 0.316661 | 1.397822 | 8.58E-12 | 2.74E-10 |
| AL353692.1 | 0.015237 | 0.047509 | 1.640648 | 2.49E-06 | 1.62E-05 |
| AC105137.1 | 0.019278 | 0.155484 | 3.011746 | 1.23E-05 | 6.52E-05 |
| RNU2-13P | 0.257676 | 0.845179 | 1.713699 | 9.87E-07 | 7.18E-06 |
| ELDR | 0.005836 | 0.012987 | 1.153875 | 0.00369 | 0.009125 |
| AL139081.1 | 0.044302 | 0.138823 | 1.647798 | 0.016252 | 0.032286 |
| SMG1P4 | 0.004657 | 0.014953 | 1.682839 | 0.000374 | 0.001262 |
| AL022323.4 | 0.011366 | 0.059355 | 2.384645 | 1.31E-05 | 6.86E-05 |
| AC100774.1 | 0.030393 | 0.200456 | 2.721468 | 1.47E-11 | 4.39E-10 |
| IGHV5-51 | 12.25335 | 42.68307 | 1.800487 | 0.009163 | 0.019861 |
| AP001266.2 | 0.044945 | 0.192141 | 2.095921 | 0.000757 | 0.002337 |
| AL139407.1 | 0.241296 | 0.529153 | 1.132879 | 3.94E-15 | 3.47E-13 |
| MTOR-AS1 | 0.016152 | 0.125401 | 2.956763 | 0.014333 | 0.029009 |
| RN7SL382P | 0.035768 | 0.155392 | 2.119165 | 8.01E-05 | 0.000331 |
| AL022323.3 | 0.00501 | 0.016375 | 1.708571 | 0.018194 | 0.035496 |
| AC104237.1 | 0.020035 | 0.041746 | 1.059108 | 1.10E-05 | 5.88E-05 |
| LENEP | 0.15056 | 0.330141 | 1.132741 | 1.48E-12 | 5.94E-11 |
| AC010531.3 | 0.059177 | 0.172798 | 1.545965 | 3.71E-12 | 1.31E-10 |
| AL021578.1 | 0.218123 | 0.518077 | 1.248026 | 3.08E-07 | 2.60E-06 |
| ZBTB20-AS1 | 0.032661 | 0.40622 | 3.636635 | 4.83E-06 | 2.88E-05 |
| AC090181.3 | 0.050206 | 0.213167 | 2.086059 | 2.15E-16 | 3.19E-14 |
| AC004678.1 | 0.064363 | 0.141949 | 1.141079 | 2.16E-08 | 2.56E-07 |
| ZBTB20-AS4 | 0.136037 | 0.393406 | 1.532019 | 0.000143 | 0.000549 |
| DIAPH2-AS1 | 0.021147 | 0.078428 | 1.8909 | 0.002355 | 0.0062 |
| AL157871.5 | 0.120322 | 0.299799 | 1.317091 | 6.94E-15 | 5.65E-13 |
| AL929601.4 | 0.03055 | 0.06674 | 1.127391 | 0.003405 | 0.008521 |
| AL592310.1 | 0.030378 | 0.082224 | 1.436504 | 0.000535 | 0.001728 |
| MIR1255A | 0.114476 | 0.570308 | 2.31669 | 0.00011 | 0.000439 |
| IGHJ3 | 2.44148 | 7.44306 | 1.60814 | 0.007389 | 0.016514 |
| AC007569.1 | 0.022581 | 0.066661 | 1.561706 | 1.31E-05 | 6.86E-05 |
| RNU6-1098P | 0.103054 | 0.418897 | 2.023189 | 6.55E-05 | 0.000278 |
| THOC7-AS1 | 0.028462 | 0.095122 | 1.740746 | 9.03E-08 | 8.99E-07 |
| MRPL37P1 | 0.096419 | 0.418666 | 2.118405 | 0.005112 | 0.012047 |
| AL161756.2 | 0.062922 | 0.190808 | 1.600483 | 1.17E-10 | 2.66E-09 |
| AL160163.1 | 0.021607 | 0.050554 | 1.226346 | 0.000184 | 0.000686 |
| AL157938.2 | 0.013285 | 0.048377 | 1.864491 | 2.80E-06 | 1.80E-05 |
| RNA5SP201 | 0.105258 | 0.346115 | 1.717316 | 8.09E-09 | 1.09E-07 |
| AL136988.2 | 0.051947 | 0.126754 | 1.286934 | 1.05E-12 | 4.38E-11 |
| AC069431.2 | 0.020811 | 0.061212 | 1.556474 | 0.002973 | 0.007578 |
| AC090774.2 | 0.05309 | 0.139142 | 1.390042 | 0.002202 | 0.005851 |
| AL513327.3 | 0.326159 | 0.751454 | 1.204107 | 1.25E-06 | 8.77E-06 |
| MIR1254-1 | 0.973904 | 2.056129 | 1.078078 | 8.81E-14 | 5.19E-12 |
| PRICKLE2-AS1 | 0.001501 | 0.006311 | 2.072469 | 0.027304 | 0.049904 |
| AC009927.1 | 0.023444 | 0.113774 | 2.27886 | 5.04E-18 | 1.42E-15 |
| AC010524.1 | 0.053599 | 0.156085 | 1.542052 | 6.74E-05 | 0.000285 |
| SNORD12B | 0.8217 | 2.340091 | 1.509881 | 4.43E-18 | 1.27E-15 |
| AC132938.5 | 0.189837 | 0.393203 | 1.050515 | 2.29E-16 | 3.34E-14 |
| ANKRD36BP2 | 0.03744 | 0.104817 | 1.485227 | 0.000563 | 0.001807 |
| AC091729.1 | 0.017584 | 0.039367 | 1.162722 | 4.32E-06 | 2.61E-05 |
| RN7SKP70 | 0.343237 | 0.855645 | 1.317809 | 4.04E-16 | 5.35E-14 |
| AL031717.1 | 0.160205 | 0.38795 | 1.275955 | 3.50E-17 | 7.05E-15 |
| RNA5SP195 | 0.094545 | 0.549906 | 2.540107 | 0.000833 | 0.002534 |
| LINC02571 | 0.325208 | 0.710591 | 1.127659 | 0.00012 | 0.000473 |
| EDRF1-AS1 | 0.10918 | 0.361068 | 1.725565 | 1.80E-20 | 1.50E-17 |
| AC018628.1 | 0.266163 | 0.600879 | 1.174763 | 0.002531 | 0.006579 |
| AL031728.1 | 0.071141 | 0.165562 | 1.218628 | 7.38E-08 | 7.54E-07 |
| AL138752.1 | 0.070907 | 0.149646 | 1.077562 | 0.000353 | 0.0012 |
| UBE2R2-AS1 | 0.164288 | 0.422893 | 1.364063 | 7.14E-08 | 7.32E-07 |
| HIF1A-AS2 | 0.070745 | 0.316804 | 2.16289 | 4.26E-06 | 2.58E-05 |
| RPL26P27 | 0.140113 | 0.34337 | 1.293171 | 1.08E-05 | 5.81E-05 |
| AC100830.1 | 0.067842 | 0.136713 | 1.010907 | 2.31E-11 | 6.40E-10 |
| AC103681.2 | 0.027622 | 0.113395 | 2.037452 | 1.77E-09 | 2.87E-08 |
| AC010306.1 | 0.022669 | 0.050368 | 1.151758 | 7.04E-05 | 0.000296 |
| TLK2P2 | 0.027502 | 0.064184 | 1.222697 | 1.65E-10 | 3.60E-09 |
| RF00586 | 0.144397 | 0.499549 | 1.790585 | 6.26E-12 | 2.07E-10 |
| PLCE1-AS1 | 0.008253 | 0.02496 | 1.59659 | 0.001632 | 0.004524 |
| AC009152.1 | 0.025685 | 0.104754 | 2.027991 | 0.000464 | 0.001525 |
| AL512656.1 | 0.123021 | 0.297614 | 1.274536 | 6.05E-13 | 2.74E-11 |
| SETP5 | 0.038125 | 0.159284 | 2.06278 | 2.81E-05 | 0.000133 |
| RNA5SP217 | 0.226441 | 0.903866 | 1.996976 | 4.42E-07 | 3.55E-06 |
| AC009133.3 | 1.177997 | 2.401172 | 1.027403 | 0.008661 | 0.018921 |
| AC011742.3 | 0.043193 | 0.249803 | 2.531928 | 2.74E-07 | 2.34E-06 |
| AC073333.1 | 0.093882 | 0.225847 | 1.266423 | 2.80E-10 | 5.70E-09 |
| AP005119.2 | 0.033909 | 0.071442 | 1.075096 | 0.020355 | 0.038965 |
| AC009542.1 | 0.023107 | 0.056414 | 1.287744 | 5.64E-09 | 7.87E-08 |
| AC005154.1 | 0.245666 | 0.603343 | 1.296279 | 7.32E-11 | 1.77E-09 |
| AC108472.1 | 0.081987 | 0.186072 | 1.182401 | 1.79E-06 | 1.22E-05 |
| AC026111.1 | 0.015055 | 0.128873 | 3.097591 | 0.000583 | 0.001859 |
| SERPIND1 | 0.083775 | 0.169896 | 1.02005 | 0.001106 | 0.003236 |
| AL355075.5 | 0.012563 | 0.027772 | 1.144472 | 0.000176 | 0.000658 |
| AC004691.1 | 0.033432 | 0.189373 | 2.501915 | 1.98E-06 | 1.32E-05 |
| HTR1DP1 | 0.016374 | 0.072597 | 2.148533 | 0.001062 | 0.003124 |
| FCAR | 0.026954 | 0.053982 | 1.001967 | 1.53E-07 | 1.41E-06 |
| SMCR5 | 0.009447 | 0.051687 | 2.451929 | 0.000889 | 0.002684 |
| RNU6ATAC16P | 0.120159 | 0.330827 | 1.461136 | 5.79E-07 | 4.50E-06 |
| LINC02362 | 0.052694 | 0.106396 | 1.013725 | 0.000131 | 0.000508 |
| AL020993.1 | 0.03409 | 0.077515 | 1.185144 | 0.000514 | 0.001668 |
| AC011510.1 | 0.051266 | 0.180604 | 1.816768 | 0.001139 | 0.003321 |
| AC097717.1 | 0.006859 | 0.014279 | 1.057842 | 2.14E-05 | 0.000105 |
| AC015802.1 | 0.019689 | 0.039969 | 1.021458 | 0.002436 | 0.006377 |
| DCTN1-AS1 | 0.011928 | 0.029293 | 1.296239 | 0.000363 | 0.001229 |
| KNOP1P2 | 0.019168 | 0.121847 | 2.668263 | 6.88E-09 | 9.39E-08 |
| AP006248.2 | 0.030228 | 0.544788 | 4.171748 | 4.54E-06 | 2.73E-05 |
| AC243654.2 | 0.044403 | 0.135985 | 1.614702 | 0.001844 | 0.005026 |
| OR5BK1P | 0.074451 | 0.223831 | 1.588052 | 1.35E-09 | 2.27E-08 |
| AC009095.1 | 0.080248 | 0.162414 | 1.01713 | 3.02E-14 | 2.03E-12 |
| AL445248.1 | 0.073752 | 0.173386 | 1.233231 | 8.04E-06 | 4.48E-05 |
| AC022445.1 | 0.067412 | 0.148666 | 1.140987 | 2.84E-05 | 0.000135 |
| AC087783.2 | 0.234283 | 0.49924 | 1.091478 | 2.10E-05 | 0.000103 |
| AC137894.1 | 0.265397 | 0.578364 | 1.123826 | 6.56E-14 | 4.03E-12 |
| SYT2 | 0.076278 | 0.155612 | 1.028611 | 2.46E-11 | 6.77E-10 |
| AC100835.1 | 0.050976 | 0.10898 | 1.096161 | 1.76E-09 | 2.86E-08 |
| AC011411.1 | 0.017336 | 0.050967 | 1.555813 | 0.014094 | 0.028601 |
| RNA5SP298 | 0.083694 | 0.17986 | 1.103687 | 6.42E-05 | 0.000272 |
| RNU6-531P | 0.932456 | 1.867804 | 1.002235 | 2.58E-07 | 2.23E-06 |
| RPL7AP2 | 0.056476 | 0.146028 | 1.37054 | 1.43E-10 | 3.18E-09 |
| AC084083.1 | 0.02477 | 0.058886 | 1.249316 | 0.000289 | 0.001007 |
| TAC3 | 0.10684 | 0.499351 | 2.224595 | 0.007504 | 0.016734 |
| AC008440.3 | 0.196391 | 0.401907 | 1.03313 | 0.001028 | 0.003037 |
| SNORD89 | 0.89758 | 1.999763 | 1.155717 | 1.63E-07 | 1.50E-06 |
| KANTR | 0.278918 | 0.566215 | 1.021507 | 2.84E-16 | 3.97E-14 |
| OR51A5P | 0.023191 | 0.100681 | 2.11815 | 0.009055 | 0.019664 |
| AC100797.1 | 0.048171 | 0.107459 | 1.157551 | 1.02E-07 | 9.96E-07 |
| MTND2P40 | 0.026749 | 0.088329 | 1.723406 | 4.05E-10 | 7.89E-09 |
| SNORA37 | 0.081897 | 0.613636 | 2.905504 | 0.003673 | 0.009089 |
| PRICKLE2-AS3 | 0.004619 | 0.045497 | 3.299984 | 0.001817 | 0.004964 |
| TET2-AS1 | 0.050828 | 0.181844 | 1.839012 | 0.000544 | 0.001752 |
| AC016590.2 | 0.319861 | 0.798092 | 1.31911 | 4.99E-16 | 6.43E-14 |
| HERC2P8 | 0.001471 | 0.003156 | 1.101244 | 0.001659 | 0.004589 |
| AL049758.1 | 0.038922 | 0.163485 | 2.070492 | 0.000838 | 0.002551 |
| AC135782.3 | 0.025696 | 0.056609 | 1.139479 | 3.88E-05 | 0.000176 |
| EEF1DP3 | 0.034383 | 0.0888 | 1.36888 | 0.000477 | 0.001561 |
| AL078645.1 | 0.080082 | 0.554078 | 2.790531 | 0.001253 | 0.003608 |
| AC107896.1 | 0.05072 | 1.162798 | 4.518908 | 1.90E-06 | 1.28E-05 |
| AC016027.5 | 0.092271 | 0.269209 | 1.54478 | 2.72E-07 | 2.33E-06 |
| EIF4EBP1P1 | 0.040619 | 0.093059 | 1.195979 | 0.000393 | 0.001319 |
| RPL7P17 | 0.055307 | 0.142257 | 1.362953 | 7.45E-08 | 7.59E-07 |
| WASHC5-AS1 | 0.115603 | 0.276465 | 1.257916 | 2.06E-11 | 5.81E-10 |
| AL161725.1 | 0.067698 | 0.15263 | 1.172839 | 2.49E-12 | 9.33E-11 |
| AC090950.2 | 0.022408 | 0.092473 | 2.045019 | 0.003079 | 0.007806 |
| AC091516.1 | 0.043317 | 0.134691 | 1.636648 | 8.86E-12 | 2.81E-10 |
| TAS2R46 | 0.022424 | 0.049967 | 1.155912 | 0.00025 | 0.000889 |
| AL162171.2 | 0.054618 | 0.134244 | 1.297421 | 0.019721 | 0.037963 |
| AC025430.1 | 0.073317 | 0.184813 | 1.333846 | 1.52E-09 | 2.53E-08 |
| MROH5 | 0.021242 | 0.04549 | 1.098639 | 3.46E-07 | 2.87E-06 |
| RNA5SP373 | 0.119412 | 0.323296 | 1.436906 | 8.63E-06 | 4.76E-05 |
| AL158166.2 | 0.016253 | 0.046308 | 1.510589 | 0.008035 | 0.017736 |
| AP003354.1 | 0.080018 | 0.275132 | 1.781721 | 9.02E-09 | 1.19E-07 |
| AC004817.3 | 0.010288 | 0.023471 | 1.189857 | 0.009012 | 0.019586 |
| AC022336.3 | 0.024653 | 0.123333 | 2.322703 | 0.000704 | 0.002191 |
| SNORA79B | 0.126901 | 0.647353 | 2.350844 | 0.000257 | 0.000912 |
| RNU6-32P | 0.064517 | 0.157683 | 1.289268 | 5.04E-06 | 2.99E-05 |
| AC092343.1 | 0.017187 | 0.092157 | 2.422791 | 0.004323 | 0.010444 |
| AP005131.3 | 0.082566 | 0.469663 | 2.508013 | 0.01811 | 0.035345 |
| SNORD116-2 | 0.234172 | 0.781022 | 1.737794 | 3.99E-06 | 2.44E-05 |
| AC093752.2 | 0.04812 | 0.096487 | 1.003704 | 1.96E-10 | 4.21E-09 |
| AC096713.1 | 0.018587 | 0.332905 | 4.162739 | 0.002973 | 0.007579 |
| RN7SKP86 | 0.044189 | 0.130616 | 1.563565 | 0.018579 | 0.036112 |
| AC008417.1 | 0.022193 | 0.056344 | 1.344165 | 8.19E-06 | 4.55E-05 |
| AP000654.1 | 0.012807 | 0.040509 | 1.661317 | 6.08E-07 | 4.69E-06 |
| RNU6-100P | 0.194849 | 0.604959 | 1.634478 | 2.62E-12 | 9.73E-11 |
| FCRL5 | 0.053476 | 0.118809 | 1.151696 | 0.010089 | 0.021558 |
| AC011379.1 | 0.0121 | 0.140208 | 3.534437 | 1.72E-05 | 8.68E-05 |
| AL357134.1 | 0.09652 | 0.357179 | 1.887746 | 0.000284 | 0.000993 |
| AC011944.2 | 0.024305 | 0.175255 | 2.850138 | 0.000479 | 0.001566 |
| AL162431.1 | 0.02392 | 0.050484 | 1.077633 | 7.49E-05 | 0.000313 |
| HIST2H2BB | 0.399525 | 0.877014 | 1.134314 | 4.76E-05 | 0.00021 |
| GAPDHP42 | 0.019849 | 0.091344 | 2.202252 | 2.79E-05 | 0.000133 |
| AC099804.1 | 0.032935 | 0.325354 | 3.304338 | 7.41E-05 | 0.00031 |
| HIGD1AP16 | 0.065569 | 0.269851 | 2.041085 | 0.010266 | 0.021887 |
| SNORD116-13 | 0.14582 | 0.667721 | 2.195052 | 9.79E-06 | 5.33E-05 |
| AF127577.6 | 0.011086 | 0.267547 | 4.592973 | 5.04E-05 | 0.000221 |
| DPRXP4 | 0.133117 | 0.288034 | 1.113541 | 1.31E-08 | 1.66E-07 |
| AL121759.1 | 0.016046 | 0.082582 | 2.363601 | 2.37E-10 | 4.94E-09 |
| AC010530.1 | 0.114584 | 0.233408 | 1.026448 | 1.34E-13 | 7.41E-12 |
| AC109460.2 | 0.313478 | 0.650089 | 1.052273 | 3.51E-21 | 5.09E-18 |
| EGR3 | 4.177544 | 8.666927 | 1.052865 | 0.000102 | 0.000408 |
| AC004024.1 | 0.056932 | 0.13406 | 1.235559 | 0.000101 | 0.000407 |
| AC134775.1 | 0.076808 | 0.28076 | 1.870018 | 9.75E-14 | 5.61E-12 |
| IGHV3-71 | 0.137198 | 0.418291 | 1.608245 | 0.013198 | 0.027093 |
| AC245884.1 | 0.042848 | 0.094401 | 1.139582 | 3.61E-09 | 5.33E-08 |
| LINC01449 | 0.010055 | 0.02487 | 1.306474 | 0.000208 | 0.000762 |
| AC020916.1 | 5.022231 | 10.22166 | 1.02523 | 3.14E-06 | 1.98E-05 |
| GLYATL1P2 | 0.249916 | 1.058737 | 2.08283 | 0.000158 | 0.000601 |
| INO80-AS1 | 0.045044 | 0.107695 | 1.257563 | 4.61E-05 | 0.000205 |
| AL356753.1 | 0.004628 | 0.02279 | 2.299994 | 7.22E-06 | 4.08E-05 |
| AL354733.3 | 0.750396 | 1.743072 | 1.215908 | 1.29E-18 | 4.49E-16 |
| EFCAB14-AS1 | 0.061949 | 0.161247 | 1.380106 | 2.69E-12 | 9.96E-11 |
| B3GALNT1P1 | 0.018196 | 0.142199 | 2.966221 | 0.001514 | 0.004242 |
| CEACAM20 | 4.2427 | 8.720485 | 1.039426 | 0.003 | 0.007641 |
| TBL1XR1-AS1 | 0.056722 | 0.409875 | 2.853203 | 0.003652 | 0.009044 |
| AC087241.4 | 0.038705 | 0.082552 | 1.092793 | 8.91E-07 | 6.55E-06 |
| AP005131.1 | 0.018863 | 0.304795 | 4.014236 | 2.87E-11 | 7.78E-10 |
| AC007619.1 | 0.153383 | 0.395378 | 1.366096 | 2.78E-08 | 3.19E-07 |
| AC055733.3 | 0.02266 | 0.240645 | 3.408661 | 0.000942 | 0.002822 |
| MED28P7 | 0.041749 | 0.089212 | 1.095487 | 2.12E-06 | 1.41E-05 |
| PWAR5 | 0.492137 | 1.575288 | 1.678483 | 8.05E-11 | 1.91E-09 |
| NCBP2-AS1 | 0.13803 | 0.279136 | 1.015988 | 1.78E-16 | 2.71E-14 |
| RPL18P10 | 0.042932 | 0.318593 | 2.891574 | 0.002777 | 0.007139 |
| RN7SKP239 | 0.210641 | 0.525773 | 1.319652 | 4.33E-11 | 1.11E-09 |
| AL392023.2 | 0.039649 | 0.085034 | 1.10075 | 2.65E-07 | 2.28E-06 |
| AC092641.1 | 0.062347 | 0.210065 | 1.752457 | 1.28E-11 | 3.90E-10 |
| RF00411 | 0.03785 | 0.106146 | 1.487668 | 2.88E-07 | 2.45E-06 |
| AL603756.1 | 0.052486 | 0.124542 | 1.246633 | 3.32E-17 | 6.79E-15 |
| AC007000.3 | 0.051159 | 0.14356 | 1.488588 | 7.04E-05 | 0.000296 |
| ACTG1P20 | 0.452059 | 0.962947 | 1.090944 | 3.84E-12 | 1.34E-10 |
| AC005277.2 | 0.056693 | 0.116701 | 1.041568 | 3.15E-05 | 0.000147 |
| AC009336.2 | 0.02137 | 0.044227 | 1.049326 | 5.72E-12 | 1.91E-10 |
| AC046158.3 | 0.005428 | 0.079178 | 3.866709 | 0.002615 | 0.006772 |
| SND1-IT1 | 0.012199 | 0.189688 | 3.958781 | 0.00073 | 0.002264 |
| AC116366.1 | 0.33374 | 0.799273 | 1.259965 | 1.60E-16 | 2.52E-14 |
| AC104024.1 | 0.009081 | 0.019712 | 1.118115 | 0.017349 | 0.034099 |
| FERP1 | 0.128153 | 0.265934 | 1.053207 | 3.06E-17 | 6.35E-15 |
| AL357874.2 | 0.025651 | 0.065986 | 1.363139 | 4.08E-06 | 2.49E-05 |
| AL136115.1 | 0.050226 | 0.282561 | 2.492066 | 3.78E-10 | 7.43E-09 |
| AL357992.1 | 0.10409 | 0.285584 | 1.456091 | 0.00011 | 0.000439 |
| TATDN1P1 | 0.056251 | 0.158341 | 1.493097 | 0.025376 | 0.046962 |
| TAS2R63P | 0.017608 | 0.057385 | 1.704432 | 4.35E-05 | 0.000194 |
| AL117381.1 | 0.065696 | 0.530089 | 3.012366 | 2.03E-05 | 0.0001 |
| AC138473.1 | 0.050704 | 0.116043 | 1.19448 | 1.52E-07 | 1.41E-06 |
| AL079301.1 | 0.118492 | 0.284724 | 1.264768 | 3.69E-06 | 2.29E-05 |
| AF131216.1 | 0.033662 | 0.103509 | 1.620554 | 8.62E-19 | 3.17E-16 |
| AC104791.2 | 0.067408 | 0.242167 | 1.845017 | 3.22E-08 | 3.63E-07 |
| AL136115.2 | 0.066264 | 0.206932 | 1.64287 | 2.03E-08 | 2.44E-07 |
| AC026348.1 | 0.045759 | 0.144592 | 1.659868 | 4.92E-09 | 7.00E-08 |
| AC104699.1 | 0.09565 | 0.335845 | 1.811963 | 5.41E-05 | 0.000235 |
| AC004692.2 | 0.019519 | 0.04475 | 1.196998 | 0.024118 | 0.044995 |
| RN7SL812P | 0.114212 | 0.284094 | 1.314652 | 4.74E-06 | 2.83E-05 |
| RNU6-282P | 0.233899 | 0.472106 | 1.013223 | 7.32E-09 | 9.90E-08 |
| AC130456.1 | 0.056981 | 0.124498 | 1.127568 | 3.19E-05 | 0.000149 |
| AL356356.1 | 0.173435 | 0.588877 | 1.763569 | 6.79E-15 | 5.54E-13 |
| LINC01303 | 0.009787 | 0.026956 | 1.461637 | 0.005706 | 0.013235 |
| AC013714.1 | 0.009351 | 0.041608 | 2.153714 | 4.02E-07 | 3.27E-06 |
| HAS3 | 0.638027 | 1.467601 | 1.201771 | 0.018845 | 0.036556 |
| LINC02099 | 0.009774 | 0.021314 | 1.124702 | 0.018049 | 0.035232 |
| SEC13P1 | 0.015302 | 0.068694 | 2.166433 | 1.98E-09 | 3.16E-08 |
| AL020995.1 | 0.009877 | 0.048306 | 2.290039 | 0.000116 | 0.000458 |
| AL359233.1 | 0.030924 | 0.069925 | 1.177095 | 0.000347 | 0.001184 |
| CCR8 | 0.105857 | 0.218834 | 1.047717 | 3.15E-06 | 1.99E-05 |
| HSD3BP4 | 0.035624 | 0.098405 | 1.465857 | 0.006519 | 0.014825 |
| DDX18P3 | 0.009944 | 0.022909 | 1.20405 | 1.22E-05 | 6.44E-05 |
| AC105389.2 | 0.171111 | 0.485174 | 1.503567 | 1.37E-12 | 5.56E-11 |
| AC092135.3 | 0.020536 | 0.098213 | 2.257733 | 0.001654 | 0.004578 |
| AC002400.1 | 0.041167 | 0.220375 | 2.420411 | 1.26E-08 | 1.61E-07 |
| HMGB3P14 | 0.030265 | 0.150367 | 2.312785 | 7.91E-09 | 1.06E-07 |
| AC005480.1 | 0.021775 | 0.058601 | 1.42826 | 5.33E-08 | 5.66E-07 |
| SPINK7 | 0.012832 | 0.035064 | 1.450301 | 1.46E-09 | 2.43E-08 |
| AC022400.2 | 0.013232 | 0.074952 | 2.501887 | 0.000496 | 0.001617 |
| AC007991.2 | 0.100967 | 0.31954 | 1.662119 | 3.99E-12 | 1.39E-10 |
| AC004893.1 | 0.024571 | 0.109673 | 2.158184 | 1.35E-09 | 2.27E-08 |
| AP001010.1 | 0.138912 | 0.290685 | 1.065287 | 3.13E-09 | 4.69E-08 |
| AC020951.1 | 0.056806 | 0.129752 | 1.191655 | 1.14E-09 | 1.97E-08 |
| AC122718.2 | 0.045477 | 0.359358 | 2.982205 | 1.22E-05 | 6.44E-05 |
| LINC01825 | 0.026987 | 0.061047 | 1.177647 | 6.59E-05 | 0.000279 |
| AC020765.4 | 0.027909 | 0.063806 | 1.192933 | 6.41E-05 | 0.000272 |
| AC019227.1 | 0.033116 | 0.228622 | 2.787382 | 0.022218 | 0.04198 |
| RN7SL368P | 0.043715 | 0.138629 | 1.665032 | 3.98E-06 | 2.44E-05 |
| AC008781.1 | 0.112509 | 0.254091 | 1.175302 | 6.74E-06 | 3.85E-05 |
| DUTP1 | 0.032761 | 0.12079 | 1.882462 | 0.000143 | 0.00055 |
| HEATR9 | 0.01955 | 0.042503 | 1.120373 | 2.28E-06 | 1.50E-05 |
| RNU6-1262P | 0.115783 | 0.48064 | 2.05353 | 1.78E-05 | 8.97E-05 |
| AC104365.2 | 0.110041 | 1.344244 | 3.610677 | 2.15E-06 | 1.42E-05 |
| RNU6-611P | 0.326409 | 0.957389 | 1.552424 | 5.46E-07 | 4.27E-06 |
| AC012157.1 | 0.051339 | 0.156203 | 1.605287 | 5.29E-15 | 4.46E-13 |
| AC108471.1 | 0.04062 | 0.340623 | 3.067903 | 0.002309 | 0.006099 |
| AC138956.2 | 0.4694 | 1.08906 | 1.214193 | 3.19E-20 | 2.43E-17 |
| AC022973.3 | 0.021073 | 0.103117 | 2.290842 | 4.02E-07 | 3.26E-06 |
| RPS6KA2-IT1 | 0.15853 | 0.38021 | 1.262038 | 1.41E-05 | 7.31E-05 |
| ANKRD61 | 0.238939 | 0.558728 | 1.225507 | 3.35E-20 | 2.43E-17 |
| AC012531.1 | 0.124865 | 0.340521 | 1.447378 | 8.49E-16 | 9.83E-14 |
| RF00426 | 0.026331 | 0.055234 | 1.068802 | 1.10E-05 | 5.91E-05 |
| CYP2G1P | 0.022712 | 0.04766 | 1.069294 | 6.80E-08 | 7.02E-07 |
| MIR590 | 0.709641 | 1.721926 | 1.278862 | 7.69E-17 | 1.36E-14 |
| AP001363.1 | 0.089451 | 0.312376 | 1.804116 | 6.16E-06 | 3.57E-05 |
| AC005753.1 | 0.1113 | 0.238789 | 1.10129 | 9.99E-05 | 0.000402 |
| AL359740.1 | 0.035277 | 0.350447 | 3.312399 | 0.000311 | 0.001077 |
| UBE2V2P3 | 0.050621 | 0.115199 | 1.186317 | 1.53E-10 | 3.40E-09 |
| GJA1P1 | 0.010885 | 0.036155 | 1.731863 | 0.000364 | 0.001234 |
| OR6L2P | 0.011482 | 0.025445 | 1.148042 | 0.000334 | 0.001145 |
| AC005042.1 | 0.038665 | 0.080887 | 1.064882 | 9.33E-05 | 0.000379 |
| MIR6814 | 0.125009 | 0.274711 | 1.135885 | 5.03E-07 | 3.98E-06 |
| GAPDHP32 | 0.025972 | 0.057162 | 1.138076 | 0.000241 | 0.000862 |
| MPRIP-AS1 | 0.015264 | 0.076319 | 2.321914 | 9.28E-09 | 1.22E-07 |
| C1QL1 | 0.707582 | 0.331193 | -1.09523 | 0.003656 | 0.009052 |
| AC016026.1 | 0.020098 | 0.042048 | 1.064999 | 1.40E-10 | 3.14E-09 |
| MIR421 | 0.18969 | 1.09609 | 2.530653 | 1.11E-12 | 4.58E-11 |
| KIZ-AS1 | 0.020115 | 0.060952 | 1.599409 | 3.35E-05 | 0.000156 |
| AC126389.1 | 0.094854 | 0.275108 | 1.536224 | 6.73E-05 | 0.000284 |
| PFN1P8 | 0.024577 | 0.071426 | 1.539161 | 0.025791 | 0.047586 |
| MTND5P28 | 0.024927 | 0.061679 | 1.307089 | 0.000242 | 0.000867 |
| AL353795.3 | 0.017658 | 0.121098 | 2.77777 | 2.23E-13 | 1.15E-11 |
| SLC16A6P1 | 0.106261 | 0.236852 | 1.156367 | 9.49E-13 | 4.03E-11 |
| AC010457.1 | 0.045448 | 0.127249 | 1.485361 | 0.002711 | 0.00699 |
| AC022432.1 | 0.158965 | 0.32171 | 1.017049 | 2.20E-09 | 3.46E-08 |
| AC008543.5 | 0.008117 | 0.020477 | 1.335042 | 4.92E-05 | 0.000216 |
| AC079584.2 | 0.016872 | 0.048023 | 1.509075 | 2.88E-06 | 1.84E-05 |
| AP003696.1 | 0.014327 | 0.130362 | 3.185749 | 0.000166 | 0.000625 |
| AC026403.1 | 20.0439 | 7.029505 | -1.51167 | 1.07E-10 | 2.46E-09 |
| RNU6-48P | 0.086342 | 0.231371 | 1.422071 | 9.01E-09 | 1.19E-07 |
| RN7SL449P | 0.055137 | 0.181595 | 1.719633 | 0.001994 | 0.005373 |
| NT5DC4 | 0.007666 | 0.017571 | 1.196682 | 1.60E-05 | 8.17E-05 |
| AC098935.2 | 0.033858 | 0.148931 | 2.137077 | 1.89E-06 | 1.27E-05 |
| AC139103.1 | 0.060557 | 0.130029 | 1.102469 | 1.54E-07 | 1.42E-06 |
| AC022905.1 | 0.003431 | 0.008385 | 1.289073 | 0.001271 | 0.003651 |
| IGHV1OR15-9 | 0.20524 | 0.438191 | 1.094247 | 0.024768 | 0.046021 |
| AC023818.1 | 0.201362 | 0.442777 | 1.136787 | 2.01E-23 | 1.47E-19 |
| ITPK1-AS1 | 0.003575 | 0.03682 | 3.364645 | 0.006206 | 0.014209 |
| MIR3186 | 0.293692 | 0.64661 | 1.138591 | 1.82E-11 | 5.23E-10 |
| AL008707.1 | 0.517277 | 1.107339 | 1.098089 | 0.00152 | 0.004256 |
| CXCL3 | 0.587364 | 1.644186 | 1.485046 | 0.005295 | 0.012412 |
| AC117503.2 | 0.131543 | 0.299784 | 1.188394 | 1.25E-17 | 2.97E-15 |
| AC245014.3 | 0.633639 | 3.714588 | 2.551469 | 1.88E-09 | 3.02E-08 |
| AC027796.1 | 0.058231 | 0.210329 | 1.852793 | 2.39E-08 | 2.81E-07 |
| AC073912.1 | 0.022112 | 0.062411 | 1.496985 | 0.000151 | 0.000578 |
| AC000036.1 | 0.007418 | 0.017762 | 1.259654 | 5.14E-05 | 0.000225 |
| AC012513.2 | 0.020216 | 0.373835 | 4.208806 | 0.010003 | 0.021403 |
| AC135068.2 | 0.089337 | 0.305064 | 1.771777 | 0.009479 | 0.020438 |
| EEF1B2P7 | 0.046673 | 0.103388 | 1.147415 | 0.000152 | 0.000578 |
| UBE2CP1 | 0.031389 | 0.197158 | 2.651026 | 0.003163 | 0.007991 |
| RNU6-26P | 0.164651 | 0.34154 | 1.052645 | 7.41E-07 | 5.58E-06 |
| AC016644.1 | 0.027237 | 0.090926 | 1.73913 | 0.000669 | 0.002097 |
| RNU1-73P | 0.047597 | 0.143156 | 1.588634 | 3.13E-07 | 2.63E-06 |
| AC132192.1 | 0.063948 | 0.167055 | 1.385362 | 4.50E-13 | 2.13E-11 |
| AC092279.2 | 0.336773 | 0.899152 | 1.416789 | 8.90E-18 | 2.29E-15 |
| AC093012.1 | 0.056443 | 0.15486 | 1.456095 | 1.79E-13 | 9.49E-12 |
| RNU6-856P | 0.310277 | 0.674487 | 1.120232 | 4.01E-09 | 5.83E-08 |
| ACMSD | 0.043229 | 0.088713 | 1.03715 | 2.04E-12 | 7.81E-11 |
| AC090114.3 | 0.494069 | 1.323097 | 1.421135 | 1.07E-15 | 1.22E-13 |
| BMS1P17 | 0.01864 | 0.041141 | 1.142167 | 0.000179 | 0.000667 |
| RNU4-38P | 0.063572 | 0.226845 | 1.835232 | 3.66E-06 | 2.27E-05 |
| AC011444.2 | 0.049326 | 0.152289 | 1.626378 | 0.016427 | 0.032577 |
| AC015799.1 | 0.037512 | 0.118607 | 1.660752 | 4.04E-06 | 2.47E-05 |
| AC134349.2 | 0.037059 | 0.173005 | 2.222918 | 2.55E-05 | 0.000123 |
| AKR7A2P1 | 0.05515 | 0.15879 | 1.525674 | 7.28E-12 | 2.37E-10 |
| KRT18P13 | 0.105607 | 0.047341 | -1.15754 | 0.000131 | 0.00051 |
| SMG1P3 | 0.161984 | 0.396461 | 1.291325 | 2.74E-18 | 8.40E-16 |
| RPS3AP38 | 0.110387 | 0.240415 | 1.122955 | 2.73E-08 | 3.14E-07 |
| AC015712.7 | 0.423948 | 2.551595 | 2.589439 | 1.84E-15 | 1.88E-13 |
| AC012615.4 | 0.054971 | 0.171896 | 1.644797 | 3.84E-17 | 7.67E-15 |
| AC018797.3 | 0.013332 | 0.069436 | 2.38075 | 0.011524 | 0.024134 |
| CRYZP1 | 0.109666 | 0.382916 | 1.80391 | 5.04E-17 | 9.78E-15 |
| AC010531.5 | 0.05162 | 0.135514 | 1.39245 | 4.26E-09 | 6.16E-08 |
| OPA1-AS1 | 0.049903 | 0.106901 | 1.099083 | 1.91E-10 | 4.11E-09 |
| AC103810.2 | 0.084291 | 0.184299 | 1.128588 | 0.001053 | 0.0031 |
| RPL12P9 | 0.060172 | 0.127639 | 1.084903 | 1.39E-08 | 1.75E-07 |
| AC024592.1 | 0.031047 | 0.066953 | 1.108668 | 5.50E-06 | 3.23E-05 |
| AL591806.2 | 0.178059 | 0.394568 | 1.147919 | 1.15E-05 | 6.13E-05 |
| AL445309.1 | 0.233313 | 0.59114 | 1.341232 | 1.58E-09 | 2.60E-08 |
| RNU6-1209P | 0.11756 | 0.243441 | 1.050179 | 6.99E-05 | 0.000294 |
| DBH | 0.426287 | 1.632134 | 1.936864 | 6.70E-06 | 3.83E-05 |
| SNORA80B | 0.561143 | 1.939749 | 1.78943 | 9.02E-10 | 1.59E-08 |
| ZBP1 | 0.155844 | 0.312952 | 1.005836 | 2.72E-05 | 0.000129 |
| AC073326.1 | 0.043068 | 0.116378 | 1.434142 | 0.002479 | 0.006468 |
| IGLV3-25 | 10.23825 | 28.12889 | 1.458083 | 0.00255 | 0.006625 |
| AP005131.4 | 0.012013 | 0.255295 | 4.409473 | 9.54E-05 | 0.000386 |
| BNIP3P40 | 0.037532 | 0.093 | 1.309098 | 4.92E-06 | 2.93E-05 |
| MIR4639 | 0.209802 | 0.475792 | 1.181306 | 4.67E-07 | 3.72E-06 |
| TRPA1 | 0.010337 | 0.043868 | 2.085385 | 0.007789 | 0.017268 |
| PCDHGC4 | 0.038079 | 0.082067 | 1.107807 | 5.58E-12 | 1.87E-10 |
| AC025043.1 | 0.093697 | 0.23861 | 1.348582 | 2.62E-13 | 1.31E-11 |
| CYCSP40 | 0.039561 | 0.116332 | 1.556117 | 0.008608 | 0.018825 |
| L29074.1 | 0.011716 | 0.032913 | 1.490214 | 2.09E-08 | 2.49E-07 |
| Z98885.3 | 0.069417 | 0.16158 | 1.218892 | 2.58E-15 | 2.44E-13 |
| AC006330.1 | 0.087251 | 0.205763 | 1.237743 | 3.98E-11 | 1.03E-09 |
| AP004289.1 | 0.004498 | 0.073702 | 4.034391 | 4.78E-06 | 2.86E-05 |
| AC120193.1 | 0.030461 | 0.100771 | 1.726057 | 2.91E-05 | 0.000137 |
| AC090739.1 | 0.262073 | 0.562049 | 1.100728 | 1.74E-12 | 6.79E-11 |
| AL135787.1 | 0.075152 | 0.179759 | 1.258176 | 0.000223 | 0.000805 |
| AC009268.1 | 0.010962 | 0.028441 | 1.375507 | 0.008101 | 0.017861 |
| HNRNPA1P40 | 0.022448 | 0.077712 | 1.791564 | 0.017343 | 0.034088 |
| CDCA4P1 | 0.081547 | 0.241212 | 1.564595 | 1.84E-10 | 3.97E-09 |
| AP001885.1 | 0.015952 | 0.103107 | 2.692317 | 0.000955 | 0.002852 |
| AC091544.7 | 0.094918 | 0.313037 | 1.721578 | 0.000106 | 0.000424 |
| AC117834.1 | 0.033857 | 0.095725 | 1.499425 | 7.19E-08 | 7.37E-07 |
| HNRNPA1P49 | 0.120777 | 0.399041 | 1.724194 | 8.76E-18 | 2.28E-15 |
| LINC00517 | 0.016705 | 0.094411 | 2.498691 | 6.68E-05 | 0.000283 |
| SEC62-AS1 | 0.154453 | 0.343582 | 1.153489 | 1.61E-14 | 1.18E-12 |
| AL031666.3 | 0.030287 | 0.143912 | 2.248404 | 4.51E-09 | 6.48E-08 |
| MIR4664 | 5.116073 | 10.23677 | 1.000653 | 1.58E-11 | 4.65E-10 |
| CADM2-AS1 | 0.008469 | 0.017476 | 1.045168 | 0.004609 | 0.011037 |
| IFNG | 0.094001 | 0.192953 | 1.037506 | 0.000749 | 0.002315 |
| AC233300.1 | 0.131576 | 0.428148 | 1.702218 | 1.70E-19 | 8.18E-17 |
| GAS8-AS1 | 0.126468 | 0.289316 | 1.19388 | 1.45E-13 | 7.95E-12 |
| SLC6A1-AS1 | 0.025551 | 0.055886 | 1.129127 | 2.87E-06 | 1.84E-05 |
| AC023825.2 | 0.021311 | 0.10573 | 2.310684 | 7.99E-07 | 5.95E-06 |
| EIF4EP1 | 0.139474 | 0.378335 | 1.439665 | 3.36E-15 | 3.07E-13 |
| AC007216.2 | 0.179823 | 0.388818 | 1.112522 | 2.92E-07 | 2.48E-06 |
| CR936218.1 | 0.451101 | 1.105214 | 1.292804 | 2.44E-14 | 1.68E-12 |
| MED15P6 | 0.047292 | 0.242426 | 2.357878 | 7.95E-07 | 5.93E-06 |
| AMZ1 | 0.037606 | 0.106389 | 1.500327 | 2.02E-10 | 4.32E-09 |
| AC007036.1 | 0.038092 | 0.079587 | 1.063026 | 0.011012 | 0.023216 |
| IGHV4-55 | 0.597527 | 1.776554 | 1.572006 | 0.01078 | 0.022808 |
| C11orf91 | 0.028272 | 0.075338 | 1.413985 | 0.000225 | 0.000812 |
| AC007991.4 | 0.021435 | 0.130194 | 2.602645 | 3.18E-07 | 2.67E-06 |
| SNORA71C | 0.605901 | 1.300496 | 1.101906 | 1.98E-14 | 1.42E-12 |
| AC007204.1 | 0.027397 | 0.061222 | 1.160057 | 0.000708 | 0.002203 |
| MIR3192 | 0.092872 | 0.412088 | 2.149633 | 4.86E-08 | 5.22E-07 |
| PDSS1P1 | 0.009547 | 0.102582 | 3.425628 | 0.000119 | 0.000469 |
| AP000786.1 | 0.151772 | 0.405922 | 1.419302 | 8.05E-12 | 2.59E-10 |
| LINC01293 | 0.073068 | 0.19419 | 1.410148 | 4.51E-06 | 2.71E-05 |
| POLR2KP1 | 0.151393 | 0.333071 | 1.137526 | 0.000206 | 0.000753 |
| AL161757.2 | 0.02833 | 0.066949 | 1.240743 | 9.26E-07 | 6.78E-06 |
| AC010329.5 | 0.005539 | 0.014415 | 1.379845 | 0.003045 | 0.007734 |
| PRDX3P1 | 0.249559 | 0.649316 | 1.379541 | 5.79E-20 | 3.40E-17 |
| PKHD1 | 0.081324 | 0.172112 | 1.081598 | 0.000396 | 0.001327 |
| HLA-T | 0.07677 | 0.161772 | 1.075345 | 7.42E-06 | 4.18E-05 |
| AL512637.1 | 0.009077 | 0.040878 | 2.171071 | 7.25E-11 | 1.75E-09 |
| MIR7856 | 0.293106 | 0.64207 | 1.131308 | 0.000173 | 0.00065 |
| MAGEB17 | 0.18252 | 0.399125 | 1.128785 | 0.000202 | 0.000742 |
| ANKRD30A | 0.153334 | 0.827863 | 2.432717 | 0.002659 | 0.006875 |
| AL590762.4 | 0.039515 | 0.332833 | 3.074341 | 0.00027 | 0.000951 |
| AC131971.1 | 0.024282 | 0.26335 | 3.439033 | 4.42E-14 | 2.86E-12 |
| AC073569.1 | 0.03137 | 0.140537 | 2.163514 | 5.91E-08 | 6.21E-07 |
| RF00190 | 0.183322 | 0.491633 | 1.423199 | 0.025222 | 0.046724 |
| AC099684.1 | 0.010432 | 0.027069 | 1.375633 | 0.000464 | 0.001525 |
| AC015853.1 | 0.065443 | 0.131414 | 1.005814 | 3.69E-06 | 2.29E-05 |
| KCNQ1OT1 | 0.056471 | 0.197538 | 1.806539 | 4.43E-13 | 2.10E-11 |
| AC106772.1 | 0.139085 | 0.324209 | 1.22096 | 1.47E-05 | 7.57E-05 |
| Z83313.1 | 0.040845 | 0.153791 | 1.912745 | 0.000906 | 0.002728 |
| AL022238.1 | 0.04371 | 0.154669 | 1.823133 | 3.93E-09 | 5.74E-08 |
| Z95152.1 | 0.052317 | 0.285999 | 2.450661 | 1.21E-12 | 4.99E-11 |
| BRD7P4 | 0.015825 | 0.13046 | 3.043304 | 0.018657 | 0.036247 |
| AL365356.1 | 0.017616 | 0.082267 | 2.223394 | 8.58E-11 | 2.02E-09 |
| AL162431.4 | 0.029512 | 0.090541 | 1.617248 | 0.004507 | 0.010827 |
| AL121601.2 | 0.018147 | 0.079216 | 2.126029 | 9.04E-12 | 2.86E-10 |
| AC109326.1 | 0.803067 | 1.993573 | 1.311764 | 0.000151 | 0.000576 |
| AC139783.2 | 0.086566 | 0.200169 | 1.209339 | 0.013312 | 0.027282 |
| CLDN20 | 0.082345 | 0.248009 | 1.590634 | 1.56E-12 | 6.22E-11 |
| AC112719.2 | 0.006576 | 0.019031 | 1.533136 | 0.013835 | 0.028156 |
| IGLV3-16 | 0.152533 | 0.337921 | 1.147559 | 0.00343 | 0.008576 |
| RN7SL441P | 0.043491 | 0.104344 | 1.262571 | 4.11E-09 | 5.96E-08 |
| AC100830.2 | 0.284903 | 0.592908 | 1.057335 | 6.35E-09 | 8.76E-08 |
| MTND1P8 | 0.080976 | 0.264631 | 1.708417 | 1.81E-11 | 5.22E-10 |
| AC025918.1 | 0.043336 | 0.10755 | 1.311364 | 2.04E-09 | 3.24E-08 |
| PHBP12 | 0.071627 | 0.206791 | 1.529594 | 3.42E-07 | 2.84E-06 |
| AL136164.3 | 0.023156 | 0.100601 | 2.119164 | 0.000443 | 0.001463 |
| IL1RL1 | 0.146594 | 0.297613 | 1.021613 | 0.017864 | 0.034936 |
| AC007922.2 | 0.031296 | 0.087329 | 1.480464 | 0.000294 | 0.001025 |
| AC009563.1 | 0.017287 | 0.049997 | 1.532155 | 0.001536 | 0.004296 |
| AL109614.1 | 0.235516 | 0.523943 | 1.153582 | 5.60E-15 | 4.68E-13 |
| AC110048.2 | 0.012511 | 0.052533 | 2.070052 | 0.000169 | 0.000638 |
| NPTN-IT1 | 0.218626 | 0.615488 | 1.493267 | 3.15E-16 | 4.28E-14 |
| AC004223.1 | 0.056709 | 0.177019 | 1.642241 | 0.000542 | 0.001746 |
| SNORA73B | 4.568213 | 73.75318 | 4.013003 | 0.005812 | 0.01344 |
| AC007362.1 | 0.008962 | 0.046877 | 2.387011 | 1.44E-05 | 7.44E-05 |
| GOLGA8O | 0.010631 | 0.02181 | 1.036677 | 1.10E-07 | 1.06E-06 |
| FPR2 | 0.042648 | 0.089637 | 1.071633 | 4.18E-06 | 2.55E-05 |
| AC253536.3 | 0.272551 | 0.764491 | 1.487971 | 2.90E-16 | 4.00E-14 |
| AL589935.1 | 0.026891 | 0.071725 | 1.415346 | 0.000142 | 0.000546 |
| RAD17P1 | 0.021963 | 0.055278 | 1.33161 | 6.61E-06 | 3.78E-05 |
| AC018761.3 | 0.038376 | 0.219032 | 2.512848 | 2.76E-09 | 4.22E-08 |
| HERC2P10 | 0.030805 | 0.069686 | 1.17773 | 1.19E-09 | 2.04E-08 |
| RNA5SP85 | 0.114418 | 0.347707 | 1.603559 | 5.40E-05 | 0.000235 |
| CRHR2 | 0.038993 | 0.154613 | 1.987385 | 0.005528 | 0.012877 |
| AC036103.1 | 0.135833 | 0.423221 | 1.639574 | 6.22E-20 | 3.53E-17 |
| AC023271.1 | 0.021961 | 0.048575 | 1.145265 | 1.93E-11 | 5.50E-10 |
| U2AF1 | 0.052697 | 0.113205 | 1.103159 | 2.92E-11 | 7.88E-10 |
| RPS26P49 | 0.031931 | 0.065482 | 1.03613 | 0.001781 | 0.004877 |
| LINC00843 | 0.180536 | 0.376486 | 1.06031 | 6.22E-14 | 3.87E-12 |
| MTATP8P2 | 5.633776 | 17.62704 | 1.645616 | 0.007437 | 0.016608 |
| RNU6-418P | 2.369415 | 4.958204 | 1.065287 | 5.38E-12 | 1.81E-10 |
| TPRKBP2 | 0.013406 | 0.054551 | 2.024749 | 7.14E-09 | 9.68E-08 |
| RPL7P57 | 0.058465 | 0.161256 | 1.463707 | 1.07E-12 | 4.47E-11 |
| RNU6-915P | 0.076278 | 0.232379 | 1.607142 | 1.80E-09 | 2.92E-08 |
| RN7SL806P | 0.044805 | 0.111343 | 1.313272 | 0.000264 | 0.000933 |
| SLC8A1-AS1 | 0.010376 | 0.04958 | 2.256485 | 0.012763 | 0.026327 |
| AL360091.1 | 0.074194 | 0.164873 | 1.15198 | 2.62E-10 | 5.39E-09 |
| AC093583.1 | 0.013993 | 0.128937 | 3.203942 | 1.92E-06 | 1.29E-05 |
| AL132657.2 | 0.036719 | 0.10396 | 1.501432 | 0.018475 | 0.035935 |
| AL445928.1 | 0.065242 | 0.176316 | 1.434279 | 2.44E-07 | 2.12E-06 |
| AC069499.2 | 0.037736 | 0.12302 | 1.704901 | 4.64E-09 | 6.64E-08 |
| AC024267.4 | 0.242379 | 0.624279 | 1.364928 | 2.58E-11 | 7.04E-10 |
| COL4A2-AS1 | 0.068499 | 0.144602 | 1.077932 | 2.65E-05 | 0.000127 |
| AC005326.1 | 0.1318 | 0.471731 | 1.839613 | 0.003028 | 0.0077 |
| STARD13-IT1 | 0.024314 | 0.158747 | 2.70688 | 6.55E-06 | 3.76E-05 |
| AC025917.1 | 0.326716 | 0.690145 | 1.078864 | 1.54E-20 | 1.42E-17 |
| RPS20P15 | 0.030807 | 0.196159 | 2.670715 | 1.41E-05 | 7.31E-05 |
| TMPRSS11F | 0.059456 | 0.165488 | 1.476827 | 0.005435 | 0.012696 |
| AL138725.1 | 0.040442 | 0.089895 | 1.152382 | 0.000358 | 0.001216 |
| OR4K12P | 0.132617 | 0.309751 | 1.223838 | 0.000917 | 0.002757 |
| AP001767.4 | 0.367118 | 0.830067 | 1.176984 | 1.71E-15 | 1.77E-13 |
| KCNA3 | 0.137186 | 0.326254 | 1.249865 | 6.40E-06 | 3.69E-05 |
| CCKAR | 0.035899 | 0.07834 | 1.125787 | 0.000409 | 0.001363 |
| RPS12P20 | 0.031283 | 0.095118 | 1.604338 | 0.000224 | 0.000808 |
| AP001269.1 | 0.018201 | 0.037622 | 1.047571 | 0.005778 | 0.013372 |
| SNX19P3 | 0.018194 | 0.042659 | 1.229399 | 0.002894 | 0.007404 |
| AC131212.2 | 0.207559 | 0.541627 | 1.383775 | 3.01E-17 | 6.33E-15 |
| AC107021.1 | 0.025885 | 0.06965 | 1.428015 | 0.000212 | 0.000774 |
| SLC10A5 | 0.548646 | 1.098468 | 1.001546 | 1.37E-05 | 7.12E-05 |
| ANKRD36 | 0.203637 | 0.439239 | 1.109005 | 2.07E-16 | 3.08E-14 |
| MLN | 0.019449 | 0.059839 | 1.621402 | 0.000624 | 0.001974 |
| AC011443.1 | 0.034579 | 0.136173 | 1.977455 | 0.000189 | 0.000703 |
| PLAC4 | 0.094227 | 0.86695 | 3.201734 | 6.72E-09 | 9.20E-08 |
| AC005740.3 | 0.116531 | 0.23833 | 1.032248 | 5.68E-06 | 3.33E-05 |
| AC004584.3 | 0.02268 | 0.052768 | 1.218245 | 1.56E-07 | 1.44E-06 |
| AL445471.1 | 0.027368 | 0.084794 | 1.631474 | 0.019755 | 0.038002 |
| TYRO3P | 0.012203 | 0.028952 | 1.246363 | 5.52E-06 | 3.25E-05 |
| AC133473.1 | 0.06907 | 0.281184 | 2.025388 | 9.57E-10 | 1.68E-08 |
| TLK1P1 | 0.005172 | 0.011765 | 1.185806 | 0.007325 | 0.016394 |
| RPL7L1P3 | 0.023052 | 0.09569 | 2.05347 | 8.03E-05 | 0.000332 |
| IGKV1OR2-108 | 0.478435 | 1.525558 | 1.672943 | 0.005162 | 0.012146 |
| DUTP2 | 0.032478 | 0.111828 | 1.783753 | 2.97E-08 | 3.38E-07 |
| AC092139.3 | 0.005109 | 0.079748 | 3.964188 | 0.000338 | 0.001158 |
| AC004884.2 | 0.106299 | 0.259613 | 1.288238 | 4.95E-05 | 0.000217 |
| AP002907.1 | 0.327998 | 0.676032 | 1.043404 | 3.18E-15 | 2.94E-13 |
| AC007834.2 | 0.072568 | 0.161359 | 1.152867 | 3.11E-09 | 4.67E-08 |
| RPS20P22 | 0.09534 | 0.197989 | 1.054265 | 0.001187 | 0.003444 |
| AC068657.1 | 0.04892 | 0.121996 | 1.318335 | 0.000666 | 0.002089 |
| AC004494.1 | 0.048609 | 0.117848 | 1.277621 | 8.21E-15 | 6.43E-13 |
| FAM53B-AS1 | 0.016788 | 0.061536 | 1.873964 | 0.004175 | 0.010146 |
| EBLN2 | 0.24936 | 0.595733 | 1.256438 | 1.38E-23 | 1.47E-19 |
| MTRNR2L4 | 0.063847 | 0.131944 | 1.04724 | 4.35E-11 | 1.12E-09 |
| SNRPGP4 | 0.245379 | 0.712722 | 1.53833 | 1.28E-11 | 3.90E-10 |
| AC006254.2 | 0.050653 | 0.118328 | 1.224077 | 0.000608 | 0.00193 |
| BIRC6-AS2 | 0.054719 | 0.142395 | 1.37979 | 4.82E-10 | 9.20E-09 |
| HNRNPA1P68 | 0.021835 | 0.324804 | 3.894858 | 1.61E-05 | 8.21E-05 |
| FLG2 | 0.004057 | 0.01084 | 1.41784 | 0.000261 | 0.000924 |
| SNORD13P1 | 0.080313 | 0.186004 | 1.211631 | 7.80E-06 | 4.36E-05 |
| ATP8A2P2 | 0.026446 | 0.159206 | 2.589778 | 1.80E-06 | 1.22E-05 |
| AC006042.3 | 0.078186 | 0.164714 | 1.074975 | 5.57E-08 | 5.89E-07 |
| LINC01482 | 0.005546 | 0.023561 | 2.086934 | 0.000193 | 0.000713 |
| AL449212.1 | 0.97741 | 2.018906 | 1.046538 | 1.88E-17 | 4.18E-15 |
| EVX1-AS | 0.316391 | 0.714788 | 1.175809 | 1.86E-05 | 9.30E-05 |
| IRS4 | 0.115204 | 0.618724 | 2.425104 | 0.003257 | 0.008199 |
| AC008895.1 | 0.098775 | 0.229357 | 1.215371 | 9.45E-12 | 2.97E-10 |
| AC068446.1 | 0.046519 | 0.103934 | 1.159757 | 2.60E-09 | 3.99E-08 |
| AL365475.1 | 0.009891 | 0.024586 | 1.313658 | 9.22E-05 | 0.000375 |
| AC011611.5 | 0.059473 | 0.131833 | 1.148398 | 1.77E-18 | 5.82E-16 |
| AC010378.1 | 0.048335 | 0.177557 | 1.877128 | 1.31E-07 | 1.24E-06 |
| IGKV2D-29 | 1.227649 | 3.762521 | 1.615802 | 0.005313 | 0.012452 |
| AKT3-IT1 | 0.038597 | 0.317469 | 3.040057 | 0.000512 | 0.001663 |
| AC067930.5 | 0.064197 | 0.156076 | 1.281669 | 8.47E-09 | 1.13E-07 |
| AC005514.1 | 0.057363 | 0.285794 | 2.316773 | 0.000405 | 0.001353 |
| IGFL2 | 0.035003 | 0.195177 | 2.479224 | 0.010399 | 0.022124 |
| AC024451.3 | 0.036126 | 0.072363 | 1.002224 | 0.000491 | 0.001602 |
| LINC01287 | 0.017886 | 0.066713 | 1.89918 | 7.15E-06 | 4.05E-05 |
| AC022960.1 | 0.047206 | 0.341282 | 2.853936 | 8.21E-07 | 6.10E-06 |
| RNU6-476P | 0.224735 | 0.551251 | 1.294486 | 8.26E-14 | 4.91E-12 |
| RNY1P9 | 0.162137 | 0.421666 | 1.378886 | 0.000745 | 0.002303 |
| TTC24 | 0.026123 | 0.053889 | 1.044694 | 2.73E-06 | 1.76E-05 |
| ATP5PDP4 | 0.336688 | 1.032656 | 1.616874 | 8.88E-07 | 6.54E-06 |
| RNVU1-15 | 0.430284 | 3.303272 | 2.940534 | 1.16E-07 | 1.11E-06 |
| AC068481.1 | 0.044504 | 0.13699 | 1.62205 | 2.27E-06 | 1.50E-05 |
| RPL30P2 | 0.048842 | 0.220862 | 2.17695 | 1.20E-06 | 8.46E-06 |
| PVALEF | 0.006423 | 0.015159 | 1.238897 | 5.04E-07 | 3.99E-06 |
| AL034418.1 | 0.019075 | 0.060114 | 1.656038 | 0.000189 | 0.000702 |
| SNORD53B | 0.168442 | 0.359207 | 1.092559 | 1.36E-08 | 1.72E-07 |
| AP000753.2 | 0.023209 | 0.136669 | 2.557942 | 8.12E-09 | 1.09E-07 |
| AC008737.3 | 0.08068 | 0.198474 | 1.29866 | 0.004255 | 0.010305 |
| SNORA2B | 0.0779 | 0.176103 | 1.176729 | 0.000233 | 0.000837 |
| SNORA58B | 0.0704 | 0.164102 | 1.220945 | 1.02E-05 | 5.53E-05 |
| AC026120.1 | 0.016365 | 0.037893 | 1.211337 | 0.002446 | 0.0064 |
| AL590233.1 | 0.033054 | 0.254963 | 2.947378 | 2.33E-05 | 0.000113 |
| AL137856.1 | 0.010954 | 0.035607 | 1.700708 | 1.77E-05 | 8.90E-05 |
| AC063976.2 | 0.015409 | 0.040418 | 1.39119 | 1.46E-10 | 3.24E-09 |
| AC090181.2 | 0.310745 | 0.650474 | 1.065759 | 0.001437 | 0.004059 |
| AC026401.1 | 0.014279 | 0.065393 | 2.195244 | 4.30E-06 | 2.60E-05 |
| AC073107.2 | 0.318039 | 0.641828 | 1.012983 | 2.53E-14 | 1.74E-12 |
| AC020763.3 | 0.032887 | 0.089842 | 1.449863 | 2.56E-07 | 2.21E-06 |
| CXADRP3 | 0.046081 | 0.136925 | 1.571137 | 0.00277 | 0.007125 |
| SNORD19 | 1.374551 | 3.151925 | 1.197273 | 2.42E-19 | 1.09E-16 |
| AC093311.1 | 0.046081 | 0.586007 | 3.668684 | 0.006041 | 0.013881 |
| AP002336.1 | 0.031618 | 0.08034 | 1.345355 | 0.00022 | 0.000797 |
| MIR3619 | 0.139308 | 0.46524 | 1.739695 | 0.000166 | 0.000626 |
| RNA5SP37 | 0.243808 | 0.552871 | 1.1812 | 4.52E-07 | 3.62E-06 |
| AC069236.1 | 0.023924 | 0.059453 | 1.31329 | 5.24E-05 | 0.000229 |
| AL024474.2 | 0.045581 | 0.253686 | 2.476537 | 8.70E-07 | 6.42E-06 |
| AL591721.1 | 0.08325 | 0.185495 | 1.15586 | 8.93E-08 | 8.91E-07 |
| UTS2B | 0.249989 | 0.68575 | 1.455817 | 0.007209 | 0.016165 |
| KCTD9P4 | 0.019465 | 0.041257 | 1.083741 | 1.64E-05 | 8.36E-05 |
| ZNF259P1 | 0.01276 | 0.03137 | 1.297763 | 0.001804 | 0.004935 |
| SPATC1 | 0.082555 | 0.198207 | 1.263576 | 2.20E-16 | 3.23E-14 |
| RNU6-387P | 0.31612 | 0.713496 | 1.174433 | 2.47E-11 | 6.80E-10 |
| AC094019.1 | 0.139631 | 0.317125 | 1.183428 | 9.74E-13 | 4.13E-11 |
| IL1B | 0.631588 | 1.420874 | 1.169723 | 0.000183 | 0.000683 |
| AL133243.4 | 0.016584 | 0.091249 | 2.460002 | 2.67E-07 | 2.29E-06 |
| AC130456.4 | 0.104675 | 0.224892 | 1.103313 | 0.000302 | 0.001049 |
| AC025539.1 | 0.00929 | 0.144801 | 3.96223 | 0.000424 | 0.001406 |
| CA15P1 | 0.054275 | 0.123917 | 1.191008 | 2.35E-06 | 1.54E-05 |
| AC124312.2 | 1.015878 | 2.785327 | 1.455119 | 1.71E-11 | 4.98E-10 |
| LDHAL6FP | 0.013518 | 0.02804 | 1.052562 | 0.000104 | 0.000418 |
| AL356750.1 | 0.029073 | 0.081625 | 1.489331 | 0.002101 | 0.005613 |
| SMIM32 | 2.939747 | 1.271491 | -1.20917 | 0.00141 | 0.003995 |
| RNU6-638P | 0.187868 | 0.815437 | 2.117852 | 4.37E-06 | 2.64E-05 |
| AC138956.1 | 0.272168 | 0.607358 | 1.15805 | 1.03E-17 | 2.55E-15 |
| AC008982.2 | 0.232433 | 0.696028 | 1.582331 | 6.39E-20 | 3.53E-17 |
| AC134349.1 | 0.030843 | 0.16269 | 2.399096 | 9.64E-05 | 0.000389 |
| AP000941.1 | 0.055156 | 0.213992 | 1.955977 | 2.91E-09 | 4.41E-08 |
| FAM71F1 | 0.056237 | 0.131637 | 1.226987 | 3.83E-06 | 2.36E-05 |
| SNORA14B | 0.344863 | 0.788216 | 1.192569 | 2.65E-05 | 0.000127 |
| AL359922.2 | 0.084312 | 0.221375 | 1.392677 | 5.72E-21 | 6.72E-18 |
| QRSL1P3 | 0.015535 | 0.259763 | 4.063582 | 1.94E-06 | 1.30E-05 |
| HMGB1P41 | 0.27612 | 0.648856 | 1.232605 | 1.32E-09 | 2.24E-08 |
| MIR616 | 0.636931 | 1.846528 | 1.535605 | 5.26E-22 | 1.21E-18 |
| AC008676.1 | 0.031799 | 0.08207 | 1.367885 | 6.42E-09 | 8.83E-08 |
| AC007347.1 | 0.030598 | 0.192386 | 2.652496 | 9.39E-09 | 1.24E-07 |
| AC114781.3 | 0.152317 | 0.335633 | 1.139808 | 7.17E-05 | 0.000301 |
| AC067931.2 | 0.008685 | 0.059097 | 2.766508 | 3.52E-05 | 0.000162 |
| AL158212.4 | 0.007767 | 0.030278 | 1.962744 | 0.002879 | 0.007371 |
| AC114402.2 | 0.028936 | 0.172914 | 2.57913 | 0.008162 | 0.017976 |
| SULT1C2 | 0.10608 | 0.220454 | 1.055322 | 0.000721 | 0.002239 |
| AP001207.1 | 0.030313 | 0.226427 | 2.901046 | 0.000276 | 0.000969 |
| LINC02475 | 0.036372 | 0.101626 | 1.482366 | 0.00252 | 0.006555 |
| TAS2R13 | 0.008233 | 0.056698 | 2.783813 | 0.0021 | 0.005612 |
| AC016831.4 | 0.021081 | 0.449095 | 4.413 | 7.08E-05 | 0.000298 |
| AC012485.1 | 0.029548 | 0.085483 | 1.532559 | 2.98E-10 | 6.05E-09 |
| SMTNL1 | 0.24556 | 0.520764 | 1.084553 | 1.11E-09 | 1.92E-08 |
| CLDN2 | 0.087264 | 0.21976 | 1.332473 | 0.000129 | 0.000504 |
| TTC21B-AS1 | 0.014992 | 0.070875 | 2.241075 | 1.07E-07 | 1.04E-06 |
| AC032044.1 | 0.120956 | 0.261386 | 1.111703 | 4.48E-17 | 8.82E-15 |
| LCMT1-AS2 | 0.013997 | 0.088533 | 2.661106 | 1.41E-12 | 5.72E-11 |
| IL20 | 0.045261 | 0.092364 | 1.02907 | 0.012934 | 0.026637 |
| RIMKLBP1 | 0.070041 | 0.168098 | 1.263029 | 2.08E-13 | 1.08E-11 |
| TCAM1P | 0.008546 | 0.017706 | 1.050846 | 0.020028 | 0.038438 |
| FOXP1-AS1 | 0.158301 | 0.383321 | 1.275888 | 0.02478 | 0.046027 |
| AC083837.1 | 0.101392 | 0.289502 | 1.513635 | 2.17E-08 | 2.58E-07 |
| AC135279.1 | 0.103073 | 0.24337 | 1.239484 | 9.25E-19 | 3.36E-16 |
| AC090948.2 | 0.262696 | 0.585587 | 1.15649 | 8.81E-07 | 6.49E-06 |
| AC004160.2 | 0.024986 | 0.052867 | 1.081227 | 0.009875 | 0.021184 |
| FCF1P1 | 0.037158 | 0.144141 | 1.955722 | 0.001431 | 0.004047 |
| RNU1-16P | 0.059499 | 0.316113 | 2.409508 | 2.93E-06 | 1.87E-05 |
| IGHV3-35 | 0.150301 | 0.312091 | 1.054111 | 0.014517 | 0.029331 |
| WNT1 | 0.035356 | 0.074728 | 1.079705 | 0.001244 | 0.003587 |
| SPDYA | 0.236313 | 0.552414 | 1.22505 | 8.48E-06 | 4.69E-05 |
| GUSBP9 | 0.095693 | 0.198271 | 1.050989 | 1.17E-10 | 2.66E-09 |
| AL603832.2 | 0.012555 | 0.02518 | 1.003957 | 1.81E-06 | 1.22E-05 |
| COL10A1 | 1.991624 | 4.844988 | 1.282548 | 0.000944 | 0.002825 |
| RNVU1-1 | 0.261015 | 0.813523 | 1.640052 | 3.35E-10 | 6.69E-09 |
| AC097467.3 | 0.010645 | 0.03202 | 1.588764 | 2.44E-13 | 1.24E-11 |
| SCARNA6 | 0.394694 | 4.594003 | 3.540947 | 5.34E-08 | 5.67E-07 |
| SNORD11 | 0.202164 | 0.430527 | 1.090578 | 2.01E-06 | 1.34E-05 |
| Z82217.1 | 0.016479 | 0.077144 | 2.226943 | 0.000494 | 0.001611 |
| AC023090.2 | 0.018012 | 0.036098 | 1.002928 | 2.22E-05 | 0.000108 |
| AL391822.1 | 0.056529 | 0.123406 | 1.126353 | 9.27E-05 | 0.000377 |
| AC010542.1 | 0.035512 | 0.081146 | 1.1922 | 7.61E-12 | 2.47E-10 |
| PGAM1P11 | 0.037939 | 0.077235 | 1.025567 | 6.08E-05 | 0.00026 |
| CCDC144NL | 0.08249 | 0.297086 | 1.848585 | 0.012604 | 0.026066 |
| SNORA38 | 0.120269 | 0.285948 | 1.249491 | 1.26E-06 | 8.85E-06 |
| HNRNPA1P57 | 1.146882 | 2.868132 | 1.322395 | 0.00711 | 0.01597 |
| MIR4312 | 0.159439 | 0.743095 | 2.22054 | 5.43E-10 | 1.02E-08 |
| AC138409.1 | 0.023828 | 0.087758 | 1.880855 | 4.70E-08 | 5.09E-07 |
| RN7SKP296 | 0.039953 | 0.12479 | 1.643114 | 1.47E-08 | 1.83E-07 |
| XAF1 | 0.493986 | 1.187829 | 1.265786 | 6.35E-13 | 2.85E-11 |
| IL1RAPL2 | 0.018943 | 0.04106 | 1.116065 | 0.017304 | 0.03403 |
| HMGN2P35 | 0.037268 | 0.232972 | 2.644146 | 6.32E-05 | 0.000269 |
| AC020913.3 | 0.153889 | 0.386587 | 1.328907 | 2.24E-06 | 1.48E-05 |
| NF1P6 | 0.056141 | 0.157017 | 1.48379 | 7.57E-10 | 1.37E-08 |
| AL353597.3 | 0.006823 | 0.015652 | 1.197791 | 0.003015 | 0.007673 |
| AC068790.4 | 0.201534 | 0.537888 | 1.416284 | 0.000329 | 0.001129 |
| PRAL | 0.014379 | 0.040858 | 1.506623 | 5.60E-06 | 3.29E-05 |
| AC053513.1 | 0.129714 | 0.32645 | 1.331531 | 1.08E-12 | 4.50E-11 |
| AC105129.1 | 0.02772 | 0.084315 | 1.604887 | 3.23E-05 | 0.00015 |
| RN7SL67P | 0.03916 | 0.179242 | 2.19446 | 0.000245 | 0.000872 |
| LINC00457 | 0.044042 | 0.176543 | 2.003058 | 2.41E-05 | 0.000117 |
| AC004943.1 | 0.10448 | 0.243417 | 1.220194 | 0.003041 | 0.007725 |
| SNORA80E | 0.184918 | 0.65089 | 1.815527 | 3.72E-08 | 4.13E-07 |
| C4orf45 | 0.014886 | 0.045694 | 1.618057 | 0.007323 | 0.016391 |
| RNU7-49P | 0.812021 | 1.748011 | 1.106125 | 1.69E-15 | 1.77E-13 |
| NRIR | 0.098584 | 0.266839 | 1.436544 | 1.44E-06 | 9.97E-06 |
| AL592437.1 | 0.018232 | 0.062118 | 1.768497 | 1.31E-05 | 6.83E-05 |
| HIST2H2AB | 0.368767 | 0.844639 | 1.195625 | 1.17E-05 | 6.23E-05 |
| AP006248.3 | 0.037444 | 0.099544 | 1.410584 | 1.09E-07 | 1.05E-06 |
| AL683842.1 | 0.021269 | 0.048717 | 1.195708 | 0.000455 | 0.001497 |
| AC008957.3 | 0.027472 | 0.093327 | 1.764337 | 1.05E-10 | 2.41E-09 |
| OSBPL10-AS1 | 0.010106 | 0.079591 | 2.977372 | 0.010871 | 0.022966 |
| AC007998.5 | 0.047167 | 0.18697 | 1.98695 | 4.93E-05 | 0.000217 |
| AC005726.5 | 0.00877 | 0.020623 | 1.233536 | 2.30E-07 | 2.02E-06 |
| AC005699.1 | 0.012553 | 0.077567 | 2.627445 | 0.00482 | 0.011467 |
| AC004890.3 | 0.10547 | 0.244378 | 1.212275 | 6.56E-14 | 4.03E-12 |
| NFKBIZ | 3.496236 | 8.94268 | 1.354905 | 8.16E-16 | 9.62E-14 |
| AC092681.2 | 0.048513 | 0.123249 | 1.345129 | 0.003495 | 0.008709 |
| AL121929.2 | 0.082765 | 0.180178 | 1.12232 | 0.00097 | 0.002891 |
| AC002064.1 | 0.778101 | 1.772337 | 1.187623 | 3.25E-05 | 0.000151 |
| AC087385.1 | 0.066506 | 0.436134 | 2.713223 | 0.000264 | 0.000934 |
| AC016954.1 | 0.018901 | 0.166843 | 3.141987 | 0.000273 | 0.00096 |
| ZBTB20 | 0.297544 | 1.310489 | 2.138932 | 2.78E-06 | 1.79E-05 |
| AC013553.1 | 0.043837 | 0.121092 | 1.465894 | 7.98E-05 | 0.00033 |
| MTCO1P28 | 0.072318 | 0.249579 | 1.787066 | 1.26E-12 | 5.19E-11 |
| ADAMTSL4-AS1 | 0.102592 | 0.292559 | 1.511809 | 1.85E-07 | 1.67E-06 |
| AL121839.1 | 0.008927 | 0.025487 | 1.513546 | 0.000154 | 0.000586 |
| AC139769.2 | 0.016815 | 0.080349 | 2.256484 | 2.18E-12 | 8.26E-11 |
| CXCR2P1 | 0.104495 | 0.306195 | 1.551017 | 1.38E-06 | 9.58E-06 |
| NAALADL2-AS2 | 0.14388 | 0.852293 | 2.566481 | 0.013364 | 0.027376 |
| RTL3 | 0.01165 | 0.023471 | 1.010574 | 0.000106 | 0.000424 |
| AL713922.2 | 0.052319 | 0.23277 | 2.153494 | 8.13E-12 | 2.61E-10 |
| AC105402.3 | 0.017557 | 0.111874 | 2.671719 | 8.93E-11 | 2.09E-09 |
| RN7SL426P | 0.029293 | 0.071012 | 1.277488 | 3.24E-05 | 0.000151 |
| AC010632.2 | 0.021541 | 0.056276 | 1.385433 | 7.27E-06 | 4.11E-05 |
| MIR5587 | 0.353337 | 0.894796 | 1.340514 | 1.07E-14 | 8.12E-13 |
| AREG | 1.283695 | 2.709785 | 1.077875 | 0.000338 | 0.001156 |
| AL645939.2 | 0.042345 | 0.11038 | 1.382226 | 1.22E-05 | 6.44E-05 |
| AL031600.1 | 0.430242 | 0.890021 | 1.048691 | 1.18E-14 | 8.86E-13 |
| Z94721.2 | 0.013799 | 0.028605 | 1.051752 | 2.04E-10 | 4.35E-09 |
| C18orf15 | 0.005287 | 0.086382 | 4.030253 | 0.000156 | 0.000594 |
| ERVW-1 | 0.011627 | 0.06296 | 2.43692 | 3.28E-11 | 8.69E-10 |
| AC012254.3 | 0.055377 | 0.1258 | 1.18377 | 1.79E-11 | 5.18E-10 |
| UHRF2P1 | 0.004373 | 0.100494 | 4.52243 | 0.001088 | 0.003191 |
| SLC10A1 | 0.027411 | 0.101225 | 1.884731 | 6.71E-08 | 6.94E-07 |
| AC006994.1 | 0.025665 | 0.093287 | 1.861892 | 0.000135 | 0.000523 |
| AL353801.1 | 0.041729 | 0.090673 | 1.119615 | 3.55E-05 | 0.000163 |
| AC138466.5 | 0.044634 | 0.093268 | 1.063248 | 4.56E-08 | 4.95E-07 |
| SNORD116-14 | 0.149401 | 0.555976 | 1.895829 | 4.78E-05 | 0.000211 |
| DDX18P1 | 0.036736 | 0.099246 | 1.433817 | 0.001564 | 0.004363 |
| LINC02100 | 0.118852 | 0.260237 | 1.130661 | 0.006551 | 0.014886 |
| AC096720.1 | 0.154945 | 0.835851 | 2.431494 | 2.36E-15 | 2.27E-13 |
| MIR4701 | 0.345411 | 0.728259 | 1.076135 | 2.77E-10 | 5.66E-09 |
| PPIAP41 | 0.080312 | 0.230739 | 1.522576 | 5.70E-08 | 6.02E-07 |
| AC079385.1 | 0.043263 | 0.108599 | 1.327794 | 3.24E-10 | 6.52E-09 |
| MFSD13B | 0.118517 | 0.270068 | 1.188227 | 8.24E-11 | 1.95E-09 |
| CCR4 | 0.409748 | 0.848449 | 1.050091 | 1.84E-05 | 9.22E-05 |
| RNU6-195P | 0.151264 | 0.480842 | 1.66849 | 4.15E-05 | 0.000187 |
| IGKV1-17 | 5.523012 | 15.01101 | 1.442494 | 0.004145 | 0.010082 |
| AC005828.6 | 0.059871 | 0.16685 | 1.47863 | 5.18E-08 | 5.53E-07 |
| Z83843.1 | 0.879742 | 1.855 | 1.076267 | 7.50E-17 | 1.34E-14 |
| CDC42-IT1 | 0.032117 | 0.125179 | 1.962573 | 5.76E-08 | 6.07E-07 |
| CYP4A22 | 0.004718 | 0.0106 | 1.167804 | 0.010325 | 0.021994 |
| MIR4782 | 0.185377 | 0.446278 | 1.267477 | 0.000333 | 0.001141 |
| AC124069.1 | 0.02323 | 0.172305 | 2.890895 | 0.0219 | 0.041482 |
| AC141586.4 | 0.019397 | 0.041754 | 1.106072 | 1.79E-11 | 5.17E-10 |
| AC139792.2 | 0.019786 | 0.045537 | 1.202554 | 2.34E-06 | 1.53E-05 |
| HTD2 | 0.023825 | 0.049712 | 1.061107 | 1.78E-10 | 3.86E-09 |
| PSMD6-AS2 | 0.121197 | 0.345418 | 1.51099 | 5.16E-16 | 6.61E-14 |
| IMMP1LP1 | 0.046083 | 0.119061 | 1.369394 | 0.000421 | 0.001398 |
| FOXP1-IT1 | 0.020407 | 0.347365 | 4.089337 | 1.57E-06 | 1.08E-05 |
| CEP295NL | 0.013276 | 0.02696 | 1.021997 | 0.002742 | 0.007061 |
| AC093330.2 | 0.005685 | 0.013222 | 1.217798 | 9.29E-05 | 0.000378 |
| C9orf84 | 0.036473 | 0.085532 | 1.229641 | 0.000879 | 0.002657 |
| AL096712.1 | 0.054255 | 0.162362 | 1.581394 | 5.00E-07 | 3.96E-06 |
| RNU6-1189P | 0.203996 | 0.650402 | 1.672787 | 5.16E-05 | 0.000226 |
| HIGD1AP14 | 0.058753 | 0.18016 | 1.616553 | 0.001976 | 0.005332 |
| IGHVIII-76-1 | 0.063735 | 0.143335 | 1.169227 | 0.001865 | 0.005073 |
| AC020900.1 | 0.033396 | 0.098342 | 1.558122 | 0.000145 | 0.000557 |
| AC096533.1 | 0.617744 | 1.921003 | 1.636779 | 6.68E-15 | 5.50E-13 |
| AC138207.7 | 0.043593 | 0.109696 | 1.331355 | 2.16E-07 | 1.91E-06 |
| EIF1AX-AS1 | 0.029691 | 0.083635 | 1.494068 | 2.60E-06 | 1.68E-05 |
| E2F3P2 | 0.034177 | 0.071972 | 1.07442 | 1.92E-11 | 5.48E-10 |
| RNU6-130P | 0.07195 | 0.351089 | 2.286774 | 2.67E-10 | 5.49E-09 |
| KCNV2 | 0.013301 | 0.030293 | 1.187405 | 4.23E-06 | 2.57E-05 |
| AC032044.2 | 0.213841 | 0.768207 | 1.844958 | 1.07E-07 | 1.04E-06 |
| MTND4P23 | 0.008335 | 0.063813 | 2.936538 | 6.77E-06 | 3.86E-05 |
| AC104117.1 | 0.023558 | 0.069047 | 1.551367 | 1.04E-06 | 7.51E-06 |
| AC090510.2 | 0.195079 | 0.407981 | 1.064442 | 2.71E-09 | 4.14E-08 |
| PFN1P4 | 0.036833 | 0.091961 | 1.320006 | 1.32E-09 | 2.25E-08 |
| AC002128.2 | 0.171044 | 0.458098 | 1.421286 | 5.26E-17 | 1.01E-14 |
| HSPD1P11 | 0.060494 | 0.277757 | 2.198958 | 1.86E-10 | 4.01E-09 |
| AL513365.2 | 0.290098 | 0.589741 | 1.023545 | 2.03E-09 | 3.23E-08 |
| RNA5SP317 | 0.233332 | 0.594599 | 1.349533 | 3.18E-09 | 4.77E-08 |
| AC020908.3 | 0.019746 | 0.051304 | 1.377511 | 0.001901 | 0.005152 |
| AC116158.1 | 0.03733 | 0.107275 | 1.522895 | 7.27E-07 | 5.49E-06 |
| AL590822.2 | 0.020959 | 0.043237 | 1.044716 | 6.47E-11 | 1.59E-09 |
| AC234917.1 | 0.049179 | 0.104211 | 1.083405 | 8.35E-06 | 4.62E-05 |
| AC110813.1 | 0.014699 | 0.251504 | 4.096786 | 0.001508 | 0.004232 |
| AL691432.1 | 0.472103 | 0.948705 | 1.006857 | 5.02E-15 | 4.27E-13 |
| STH | 0.019663 | 0.061792 | 1.651935 | 7.21E-07 | 5.45E-06 |
| NCLP1 | 0.062674 | 0.140134 | 1.160873 | 9.32E-12 | 2.94E-10 |
| AC087620.1 | 0.02589 | 0.098494 | 1.927637 | 2.16E-12 | 8.18E-11 |
| AC080112.3 | 0.086701 | 0.194504 | 1.165674 | 1.57E-13 | 8.53E-12 |
| HAVCR1 | 0.023469 | 0.066023 | 1.4922 | 0.005282 | 0.012391 |
| LINC01397 | 0.045175 | 0.091807 | 1.02307 | 0.000122 | 0.000477 |
| AC090515.5 | 0.032277 | 0.077359 | 1.261047 | 8.13E-07 | 6.05E-06 |
| AL445435.1 | 0.031405 | 0.24286 | 2.951071 | 4.47E-14 | 2.88E-12 |
| GOLGA6L3 | 0.013218 | 0.03029 | 1.196392 | 2.72E-06 | 1.75E-05 |
| IGHV3OR15-7 | 0.207383 | 0.417738 | 1.010299 | 0.020079 | 0.03852 |
| RN7SL12P | 0.107738 | 0.313302 | 1.540034 | 1.41E-06 | 9.76E-06 |
| AC025809.2 | 0.147704 | 0.301413 | 1.029029 | 1.99E-05 | 9.87E-05 |
| RNA5SP435 | 0.067232 | 0.263643 | 1.971356 | 8.16E-07 | 6.06E-06 |
| SNORD13E | 0.062266 | 0.137874 | 1.146835 | 1.59E-07 | 1.46E-06 |
| AL360169.2 | 0.013125 | 0.066822 | 2.348062 | 1.21E-05 | 6.41E-05 |
| AC127540.1 | 0.02609 | 0.080547 | 1.62632 | 2.48E-08 | 2.88E-07 |
| AL096817.1 | 0.005526 | 0.029205 | 2.401918 | 0.00026 | 0.00092 |
| SMPD4P1 | 0.00658 | 0.027025 | 2.038147 | 0.02485 | 0.046141 |
| AC015911.3 | 0.126616 | 0.262814 | 1.053579 | 2.31E-05 | 0.000112 |
| AC009977.1 | 0.418949 | 0.877035 | 1.065861 | 1.10E-13 | 6.24E-12 |
| OTUD4P1 | 0.010687 | 0.058121 | 2.443159 | 2.34E-05 | 0.000113 |
| RNVU1-19 | 0.564619 | 2.207762 | 1.967235 | 0.009526 | 0.020519 |
| AL450998.2 | 0.15753 | 0.335998 | 1.092829 | 5.02E-19 | 2.01E-16 |
| AC102945.1 | 0.026459 | 0.078345 | 1.566094 | 2.25E-06 | 1.48E-05 |
| AC107027.2 | 0.755939 | 1.571794 | 1.056069 | 3.46E-13 | 1.68E-11 |
| AL445931.1 | 0.057192 | 0.150858 | 1.399301 | 1.08E-20 | 1.10E-17 |
| AC072022.1 | 0.042821 | 0.223074 | 2.381146 | 2.67E-11 | 7.29E-10 |
| AC006460.1 | 0.017265 | 0.049118 | 1.508455 | 0.007023 | 0.015804 |
| IBSP | 0.046666 | 0.130135 | 1.47958 | 0.011715 | 0.024459 |
| AC130651.1 | 0.036604 | 0.098259 | 1.424606 | 2.00E-08 | 2.41E-07 |
| AC008985.1 | 0.023521 | 0.091409 | 1.958395 | 0.002076 | 0.005558 |
| RNU2-33P | 0.049073 | 0.106237 | 1.114279 | 2.82E-05 | 0.000134 |
| AL671986.1 | 0.118293 | 0.277221 | 1.228671 | 0.004397 | 0.010599 |
| Z86062.1 | 0.015947 | 0.04817 | 1.594864 | 0.000139 | 0.000536 |
| AC091153.2 | 0.032298 | 0.064873 | 1.006164 | 0.004652 | 0.011122 |
| AL157871.3 | 0.098885 | 0.495267 | 2.324388 | 1.23E-07 | 1.17E-06 |
| IRF4 | 0.213259 | 0.520509 | 1.287316 | 0.000152 | 0.00058 |
| HMGB3P9 | 0.017909 | 0.106261 | 2.568883 | 0.000681 | 0.002132 |
| CCDC144A | 0.029821 | 0.070869 | 1.248802 | 9.53E-07 | 6.96E-06 |
| AC024909.2 | 0.044342 | 0.093905 | 1.08254 | 4.54E-10 | 8.72E-09 |
| AC211433.1 | 0.039401 | 0.213926 | 2.44081 | 5.01E-12 | 1.70E-10 |
| AC079416.2 | 0.025089 | 0.09202 | 1.874873 | 0.000198 | 0.000729 |
| AC027601.5 | 0.027033 | 0.057375 | 1.085718 | 1.33E-08 | 1.67E-07 |
| PFN1P6 | 0.376455 | 0.769891 | 1.032176 | 1.24E-11 | 3.80E-10 |
| RNVU1-4 | 0.227328 | 0.598342 | 1.396194 | 0.000981 | 0.002921 |
| PHBP9 | 0.255885 | 0.555106 | 1.117269 | 2.73E-16 | 3.86E-14 |
| AP001610.1 | 0.040691 | 0.10212 | 1.327494 | 0.000612 | 0.001941 |
| AC004898.1 | 0.026809 | 0.062706 | 1.225869 | 9.31E-06 | 5.09E-05 |
| AL121672.2 | 0.331239 | 0.801843 | 1.275446 | 0.001377 | 0.003911 |
| AC138123.2 | 0.070806 | 0.215341 | 1.604679 | 1.30E-06 | 9.12E-06 |
| RN7SL81P | 0.256405 | 0.703324 | 1.455762 | 4.75E-20 | 2.98E-17 |
| AC124312.3 | 0.694641 | 1.439296 | 1.051024 | 2.05E-13 | 1.07E-11 |
| ERMN | 0.027306 | 0.063166 | 1.20992 | 0.005055 | 0.011938 |
| ISPD-AS1 | 0.014274 | 0.030755 | 1.107395 | 0.000295 | 0.001028 |
| AC055876.2 | 0.040219 | 0.097699 | 1.280456 | 1.09E-11 | 3.39E-10 |
| C1QBPP2 | 0.058748 | 0.144887 | 1.302323 | 0.017684 | 0.034658 |
| AC079880.2 | 0.022862 | 0.058505 | 1.35563 | 0.009859 | 0.02115 |
| LAMTOR3P2 | 0.149127 | 0.314034 | 1.074374 | 3.65E-07 | 3.01E-06 |
| MIR4728 | 0.395716 | 0.826476 | 1.062506 | 0.000259 | 0.000917 |
| AP001271.1 | 0.426687 | 0.866327 | 1.021733 | 4.53E-12 | 1.56E-10 |
| MKNK1-AS1 | 0.048021 | 0.098519 | 1.036726 | 2.11E-15 | 2.08E-13 |
| SNORA28 | 0.235699 | 2.325277 | 3.302386 | 9.51E-08 | 9.39E-07 |
| AL133406.1 | 0.018901 | 0.043768 | 1.211455 | 0.020127 | 0.038596 |
| AL021068.2 | 0.441542 | 1.256775 | 1.509103 | 1.50E-16 | 2.41E-14 |
| RGPD1 | 0.004117 | 0.01089 | 1.403207 | 0.000148 | 0.000566 |
| AC009996.1 | 0.15731 | 0.362118 | 1.202847 | 6.48E-14 | 3.99E-12 |
| TGFB3-AS1 | 0.058389 | 0.188165 | 1.68823 | 2.94E-06 | 1.88E-05 |
| IGHV4-31 | 2.702686 | 8.503599 | 1.653679 | 0.015566 | 0.031161 |
| SEPT7P6 | 0.021997 | 0.068087 | 1.63009 | 0.013473 | 0.027559 |
| AC245884.3 | 0.831581 | 1.72462 | 1.052349 | 1.61E-16 | 2.53E-14 |
| AC007370.2 | 0.004135 | 0.012468 | 1.592105 | 3.48E-06 | 2.17E-05 |
| AP000821.1 | 0.023879 | 0.07492 | 1.64963 | 1.93E-09 | 3.09E-08 |
| YRDCP1 | 0.015549 | 0.041111 | 1.40267 | 2.27E-05 | 0.00011 |
| RPL7AP10 | 0.230162 | 0.478786 | 1.05673 | 4.77E-11 | 1.21E-09 |
| AC087749.1 | 0.013709 | 0.1565 | 3.512966 | 1.43E-09 | 2.39E-08 |
| SUMO2P19 | 0.32563 | 0.784422 | 1.268397 | 8.60E-15 | 6.68E-13 |
| CCDC144B | 0.082482 | 0.175071 | 1.085785 | 1.89E-08 | 2.28E-07 |
| RN7SL192P | 0.147003 | 0.310112 | 1.076947 | 4.32E-14 | 2.81E-12 |
| ELOCP31 | 0.027588 | 0.090053 | 1.706715 | 0.000932 | 0.002795 |
| MAPK6P3 | 0.007381 | 0.028359 | 1.941884 | 0.008986 | 0.019542 |
| AL133284.1 | 0.005804 | 0.061415 | 3.403534 | 0.002114 | 0.005644 |
| RNY1P13 | 0.238358 | 1.259193 | 2.401298 | 5.99E-10 | 1.11E-08 |
| AC090695.1 | 0.080721 | 0.200108 | 1.309766 | 6.40E-11 | 1.57E-09 |
| AL161457.2 | 0.073024 | 0.183685 | 1.330799 | 6.05E-07 | 4.67E-06 |
| MIR378J | 0.465796 | 0.935402 | 1.005887 | 2.10E-10 | 4.46E-09 |
| AC008897.1 | 0.411013 | 0.863399 | 1.070845 | 8.71E-14 | 5.15E-12 |
| CLEC6A | 0.011782 | 0.028015 | 1.249622 | 0.004752 | 0.011329 |
| AC131182.1 | 0.024668 | 0.619612 | 4.650625 | 0.00012 | 0.000471 |
| AC018410.1 | 0.035767 | 0.212086 | 2.567959 | 2.23E-08 | 2.63E-07 |
| AC093525.9 | 0.022212 | 0.069853 | 1.652971 | 1.02E-14 | 7.81E-13 |
| JTBP1 | 0.033197 | 0.137441 | 2.049707 | 2.57E-06 | 1.67E-05 |
| AC117500.2 | 0.040037 | 0.114845 | 1.520285 | 9.94E-08 | 9.75E-07 |
| TDGF1P6 | 0.040146 | 0.106993 | 1.414209 | 0.001209 | 0.003499 |
| AC003991.1 | 0.12246 | 0.349361 | 1.512414 | 1.74E-16 | 2.67E-14 |
| AC133961.1 | 0.017154 | 0.058669 | 1.774026 | 8.81E-06 | 4.85E-05 |
| AL449363.1 | 0.251248 | 0.715757 | 1.510357 | 0.001548 | 0.004326 |
| LHX4 | 0.133562 | 0.297016 | 1.153034 | 4.20E-19 | 1.78E-16 |
| RN7SL767P | 0.107956 | 0.35298 | 1.709142 | 0.000304 | 0.001054 |
| AC087359.1 | 0.008164 | 0.024445 | 1.582184 | 0.000799 | 0.002443 |
| MIR6757 | 0.214459 | 0.497214 | 1.213166 | 3.69E-05 | 0.000169 |
| EPN2-AS1 | 0.032922 | 0.180244 | 2.452812 | 1.55E-08 | 1.91E-07 |
| AC114760.2 | 0.067058 | 0.310323 | 2.210279 | 1.40E-12 | 5.67E-11 |
| SNORD12C | 0.255348 | 0.521807 | 1.031048 | 2.39E-07 | 2.09E-06 |
| IGHV3-23 | 16.1957 | 35.77242 | 1.143237 | 0.006066 | 0.013925 |
| LIMS1-AS1 | 0.178227 | 0.494626 | 1.472625 | 1.18E-13 | 6.63E-12 |
| GK-IT1 | 0.028085 | 0.070724 | 1.332402 | 0.003285 | 0.008264 |
| GAPDHS | 0.009623 | 0.149002 | 3.952718 | 0.003753 | 0.009252 |
| AC144833.1 | 0.006196 | 0.079317 | 3.678247 | 0.004457 | 0.010721 |
| SYCP3 | 0.073706 | 0.166937 | 1.179455 | 8.25E-05 | 0.00034 |
| AP001469.1 | 0.118052 | 0.314965 | 1.415768 | 6.76E-14 | 4.14E-12 |
| AC097713.2 | 0.073654 | 0.036192 | -1.02511 | 0.000441 | 0.001457 |
| CHL1-AS1 | 0.018149 | 0.044069 | 1.279836 | 0.000363 | 0.001228 |
| AC104986.1 | 0.123797 | 0.249102 | 1.008758 | 2.12E-09 | 3.33E-08 |
| AL139807.1 | 0.056508 | 0.487836 | 3.10988 | 4.13E-05 | 0.000186 |
| IGLVIVOR22-2 | 0.01747 | 0.04781 | 1.452446 | 1.39E-08 | 1.75E-07 |
| AC004264.1 | 0.077113 | 0.158985 | 1.043854 | 6.04E-07 | 4.67E-06 |
| AL121985.1 | 0.010777 | 0.024548 | 1.187618 | 4.58E-07 | 3.66E-06 |
| AC022001.2 | 0.03229 | 0.117592 | 1.864644 | 0.006748 | 0.015261 |
| AC027290.2 | 0.151078 | 0.587637 | 1.959631 | 8.91E-21 | 9.45E-18 |
| AP005131.5 | 0.02258 | 0.461328 | 4.352669 | 0.001237 | 0.00357 |
| LINC01687 | 0.330729 | 0.802826 | 1.279438 | 0.000643 | 0.002026 |
| AC108058.1 | 0.136 | 0.445315 | 1.711216 | 6.76E-05 | 0.000285 |
| AC138393.3 | 0.412145 | 0.90875 | 1.14073 | 2.48E-14 | 1.71E-12 |
| ADAMTS4 | 1.327096 | 2.685046 | 1.016674 | 9.45E-05 | 0.000383 |
| AC018638.3 | 0.133253 | 0.306551 | 1.201958 | 7.55E-06 | 4.24E-05 |
| AL357093.1 | 0.029963 | 0.119931 | 2.000924 | 0.002063 | 0.005527 |
| PPFIA2-AS1 | 0.136864 | 0.276337 | 1.013679 | 0.000102 | 0.000408 |
| GPR52 | 0.011706 | 0.112682 | 3.26691 | 6.13E-05 | 0.000262 |
| HMGN2P31 | 0.270458 | 0.705837 | 1.383928 | 0.016049 | 0.031956 |
| AP002518.2 | 0.132536 | 0.827075 | 2.641639 | 8.93E-05 | 0.000365 |
| SLFN14 | 0.009534 | 0.022103 | 1.213035 | 5.35E-05 | 0.000233 |
| AL158166.1 | 0.061996 | 0.152181 | 1.29554 | 0.004362 | 0.010526 |
| AC020978.9 | 0.099941 | 0.312154 | 1.643115 | 2.13E-23 | 1.47E-19 |
| RAP1AP | 0.056222 | 0.126081 | 1.165149 | 6.94E-08 | 7.15E-07 |
| TEX26-AS1 | 0.004484 | 0.010041 | 1.163087 | 0.000143 | 0.00055 |
| AC092117.2 | 0.014284 | 0.037007 | 1.37342 | 0.000643 | 0.002024 |
| RPS2P41 | 0.026284 | 0.077421 | 1.558546 | 0.000209 | 0.000764 |
| SLC2A3P2 | 0.029357 | 0.202699 | 2.787587 | 7.36E-15 | 5.93E-13 |
| NCOA4P3 | 0.020032 | 0.041862 | 1.06335 | 7.65E-07 | 5.73E-06 |
| SNORA47 | 0.24065 | 5.719166 | 4.570799 | 2.01E-05 | 9.93E-05 |
| AC131009.4 | 0.090968 | 0.234733 | 1.367581 | 6.38E-15 | 5.27E-13 |
| MARK2P9 | 0.027191 | 0.057149 | 1.071593 | 5.79E-11 | 1.43E-09 |
| LINC02397 | 0.00951 | 0.019383 | 1.027248 | 0.02536 | 0.046935 |
| AC020978.1 | 0.052572 | 0.15595 | 1.568727 | 3.38E-10 | 6.75E-09 |
| AL353583.1 | 0.032047 | 0.178458 | 2.477328 | 1.31E-08 | 1.65E-07 |
| MIR593 | 0.28127 | 0.626458 | 1.155263 | 6.57E-10 | 1.21E-08 |
| AC004461.2 | 0.067136 | 0.242147 | 1.850718 | 6.55E-21 | 7.22E-18 |
| AC055811.3 | 0.031617 | 0.087201 | 1.463637 | 9.82E-09 | 1.29E-07 |
| AC118344.2 | 0.259132 | 0.521084 | 1.007828 | 7.21E-16 | 8.76E-14 |
| AC024901.1 | 0.019349 | 0.042201 | 1.125025 | 8.72E-06 | 4.81E-05 |
| AC022960.2 | 0.039437 | 0.105873 | 1.424715 | 0.004742 | 0.011309 |
| AP003108.5 | 0.859101 | 1.93953 | 1.174808 | 6.30E-16 | 7.83E-14 |
| SPAG17 | 0.046922 | 0.155515 | 1.728723 | 0.000303 | 0.001051 |
| CCNL2P1 | 0.032943 | 0.175112 | 2.410221 | 2.11E-08 | 2.52E-07 |
| AC011825.2 | 0.137926 | 0.294227 | 1.093039 | 2.21E-11 | 6.18E-10 |
| TUBB8P1 | 0.058219 | 0.171075 | 1.555078 | 2.24E-13 | 1.15E-11 |
| AC015849.3 | 0.841035 | 1.722727 | 1.034457 | 4.03E-22 | 1.11E-18 |
| MX1 | 3.659449 | 7.579579 | 1.050491 | 0.022832 | 0.043002 |
| AC136475.10 | 0.638134 | 1.352007 | 1.083171 | 3.90E-16 | 5.22E-14 |
| KCNJ13 | 0.033769 | 0.086202 | 1.352 | 0.00039 | 0.001309 |
| AC092658.1 | 0.023446 | 0.073376 | 1.645945 | 2.48E-08 | 2.88E-07 |
| AL157823.2 | 0.199665 | 0.42136 | 1.077475 | 2.51E-14 | 1.73E-12 |
| AC104984.6 | 0.037847 | 0.07624 | 1.010388 | 0.001004 | 0.00298 |
| AL355001.1 | 0.067094 | 0.141536 | 1.07692 | 0.000182 | 0.00068 |
| LIN28A | 0.003652 | 0.00939 | 1.362547 | 0.000727 | 0.002256 |
| RPL26P29 | 0.028095 | 0.060074 | 1.096442 | 8.75E-05 | 0.000358 |
| RHOT1P2 | 0.042852 | 0.097341 | 1.183691 | 0.013492 | 0.027593 |
| A3GALT2 | 0.022242 | 0.049188 | 1.144989 | 8.23E-09 | 1.10E-07 |
| GPR152 | 0.012438 | 0.025555 | 1.038804 | 1.62E-05 | 8.26E-05 |
| LINC01866 | 0.964711 | 2.362665 | 1.292246 | 0.008834 | 0.01925 |
| PZP | 0.031442 | 0.092025 | 1.549324 | 0.001234 | 0.003562 |
| MIR571 | 0.14156 | 0.461082 | 1.703604 | 0.001187 | 0.003444 |
| AL645949.1 | 0.048702 | 0.098032 | 1.009274 | 2.13E-07 | 1.88E-06 |
| AL161782.1 | 0.140355 | 0.300374 | 1.097679 | 0.000585 | 0.001868 |
| LINC02413 | 0.030104 | 0.095424 | 1.664387 | 0.017165 | 0.033804 |
| AC008147.2 | 0.04547 | 0.160876 | 1.822956 | 2.04E-14 | 1.45E-12 |
| AC010976.1 | 0.11853 | 0.263424 | 1.152128 | 6.87E-16 | 8.42E-14 |
| AC007431.1 | 0.007256 | 0.077639 | 3.419585 | 0.000175 | 0.000657 |
| AC027801.4 | 0.040451 | 0.155533 | 1.942992 | 0.000184 | 0.000684 |
| FCGR3B | 0.098935 | 0.21081 | 1.091398 | 7.40E-05 | 0.000309 |
| AC008521.1 | 0.061224 | 0.450807 | 2.880334 | 0.00011 | 0.000438 |
| AC009220.2 | 0.013983 | 0.030278 | 1.114569 | 1.27E-07 | 1.21E-06 |
| OCLM | 0.148348 | 0.532476 | 1.843733 | 5.20E-20 | 3.18E-17 |
| FAM217A | 0.014487 | 0.034942 | 1.270169 | 8.70E-15 | 6.72E-13 |
| VIPR1-AS1 | 0.257154 | 0.526898 | 1.034894 | 2.45E-16 | 3.54E-14 |
| POU5F1P4 | 0.016409 | 0.07048 | 2.102697 | 0.000781 | 0.002398 |
| RN7SKP80 | 0.727655 | 1.498221 | 1.041925 | 8.03E-10 | 1.45E-08 |
| HMGN1P8 | 0.109349 | 0.620218 | 2.503833 | 2.21E-11 | 6.18E-10 |
| AC010261.1 | 0.025615 | 0.056264 | 1.135252 | 0.000193 | 0.000713 |
| MIR374B | 0.122153 | 0.301399 | 1.302984 | 1.43E-07 | 1.33E-06 |
| SMAD9-IT1 | 0.28248 | 0.746117 | 1.401255 | 9.97E-11 | 2.31E-09 |
| AP001625.2 | 0.919011 | 1.96565 | 1.096853 | 7.21E-13 | 3.18E-11 |
| AL021407.3 | 0.074076 | 0.170089 | 1.19921 | 1.97E-10 | 4.23E-09 |
| AC097374.2 | 0.013478 | 0.031239 | 1.212746 | 0.001265 | 0.003636 |
| AL133352.1 | 0.043854 | 0.087928 | 1.003623 | 1.72E-14 | 1.24E-12 |
| AC011753.4 | 0.200065 | 0.565093 | 1.498022 | 1.57E-13 | 8.52E-12 |
| ACKR4 | 0.062055 | 0.140549 | 1.179449 | 9.20E-10 | 1.62E-08 |
| RNU6-593P | 0.073834 | 0.244632 | 1.728265 | 2.07E-08 | 2.48E-07 |
| PKP4-AS1 | 0.051078 | 0.105788 | 1.050396 | 1.69E-10 | 3.67E-09 |
| ALG13-AS1 | 0.457769 | 1.050462 | 1.198332 | 7.62E-15 | 6.13E-13 |
| AC097376.1 | 0.044676 | 0.094174 | 1.075839 | 7.38E-08 | 7.54E-07 |
| Z84723.1 | 0.027283 | 0.056864 | 1.059526 | 2.42E-06 | 1.59E-05 |
| AC005096.1 | 0.035593 | 0.120572 | 1.760235 | 0.004997 | 0.01182 |
| AL354893.2 | 0.053025 | 0.178709 | 1.752878 | 5.22E-09 | 7.36E-08 |
| COL11A1 | 0.107553 | 0.567771 | 2.400265 | 1.15E-06 | 8.15E-06 |
| AC004837.2 | 0.095188 | 0.194268 | 1.029195 | 5.35E-10 | 1.01E-08 |
| AL138963.1 | 0.072389 | 0.225224 | 1.637526 | 2.03E-05 | 0.0001 |
| AL360093.1 | 0.047212 | 0.149745 | 1.665268 | 3.37E-06 | 2.12E-05 |
| AC020728.1 | 0.011283 | 0.140687 | 3.640295 | 0.004309 | 0.010417 |
| NDUFA3P1 | 0.1031 | 0.299635 | 1.539163 | 4.05E-10 | 7.89E-09 |
| CBX1P2 | 0.074681 | 0.491038 | 2.717022 | 9.41E-05 | 0.000382 |
| SNORA12 | 0.316911 | 10.23302 | 5.013013 | 0.007561 | 0.016847 |
| TUBG1P | 0.032996 | 0.086086 | 1.383484 | 1.14E-07 | 1.09E-06 |
| CARS-AS1 | 0.006779 | 0.030121 | 2.151706 | 1.24E-05 | 6.56E-05 |
| DLEU2L | 0.071643 | 0.154408 | 1.107843 | 3.06E-16 | 4.18E-14 |
| SLC25A15P5 | 0.04975 | 0.225393 | 2.179664 | 0.001416 | 0.004009 |
| WARS2-IT1 | 0.020913 | 0.055156 | 1.399109 | 0.011268 | 0.023674 |
| AC007899.1 | 0.046658 | 0.129623 | 1.47411 | 4.26E-05 | 0.000191 |
| AC073210.1 | 0.262874 | 0.542531 | 1.045336 | 1.16E-14 | 8.76E-13 |
| AC090666.1 | 0.03578 | 0.140965 | 1.978103 | 1.22E-05 | 6.44E-05 |
| NDUFB4P6 | 0.036973 | 0.252284 | 2.770507 | 1.78E-05 | 8.94E-05 |
| AL136295.5 | 0.072831 | 0.165705 | 1.185994 | 3.58E-19 | 1.54E-16 |
| TDGF1P5 | 0.041706 | 0.201322 | 2.271186 | 5.87E-06 | 3.42E-05 |
| PLCXD3 | 0.192634 | 0.514381 | 1.416975 | 0.001637 | 0.004535 |
| ANGPTL3 | 0.603323 | 1.47655 | 1.291228 | 0.01378 | 0.028068 |
| AC092666.1 | 0.010355 | 0.073264 | 2.822797 | 8.19E-05 | 0.000338 |
| AC022211.1 | 0.088247 | 0.26399 | 1.580869 | 8.13E-18 | 2.15E-15 |
| AL035071.2 | 0.107467 | 0.322859 | 1.587011 | 0.000148 | 0.000566 |
| AP003969.1 | 0.03086 | 0.088531 | 1.520432 | 0.002603 | 0.006744 |
| IGHV4-4 | 0.343519 | 1.191147 | 1.793891 | 0.013194 | 0.027086 |
| IGHV3-15 | 7.416256 | 16.36353 | 1.141721 | 0.000909 | 0.002737 |
| LAX1 | 0.200013 | 0.418005 | 1.063422 | 2.06E-05 | 0.000102 |
| AC013472.1 | 0.012715 | 0.052838 | 2.055 | 2.61E-05 | 0.000125 |
| RPL23AP64 | 0.477963 | 1.096487 | 1.197916 | 1.00E-19 | 5.32E-17 |
| SIRPG-AS1 | 0.008295 | 0.0291 | 1.810628 | 8.41E-05 | 0.000346 |
| RF01183 | 0.387189 | 0.802774 | 1.051955 | 2.28E-08 | 2.69E-07 |
| CCAT2 | 0.022003 | 0.072805 | 1.726373 | 9.81E-13 | 4.15E-11 |
| AL354813.1 | 0.006599 | 0.015895 | 1.268286 | 0.000411 | 0.001369 |
| AL355336.1 | 0.028925 | 0.194671 | 2.750658 | 7.66E-10 | 1.39E-08 |
| RNY4P25 | 0.109718 | 0.362248 | 1.723172 | 3.25E-06 | 2.04E-05 |
| AC093904.2 | 0.034938 | 0.079296 | 1.182458 | 0.000243 | 0.000867 |
| ATP1B3-AS1 | 0.306759 | 1.165359 | 1.925596 | 5.75E-14 | 3.62E-12 |
| AL596247.1 | 0.065281 | 0.24231 | 1.892117 | 1.22E-05 | 6.44E-05 |
| AL049874.1 | 0.050688 | 0.165561 | 1.707637 | 2.29E-08 | 2.69E-07 |
| FAM131B | 0.163348 | 0.337755 | 1.048026 | 0.002483 | 0.006475 |
| SCARNA12 | 0.431005 | 4.681423 | 3.441172 | 9.18E-05 | 0.000374 |
| AL157871.2 | 0.110395 | 0.367687 | 1.735804 | 3.53E-13 | 1.70E-11 |
| AC099811.5 | 0.035009 | 0.181206 | 2.37185 | 2.43E-08 | 2.84E-07 |
| MIR4677 | 0.172248 | 0.684914 | 1.991435 | 2.61E-05 | 0.000125 |
| MTND6P21 | 0.033946 | 0.081206 | 1.258343 | 1.53E-08 | 1.89E-07 |
| RNU4-25P | 0.047134 | 0.308356 | 2.709758 | 5.07E-05 | 0.000222 |
| AP001605.1 | 0.005513 | 0.01856 | 1.751177 | 0.000382 | 0.001286 |
| IL6STP1 | 0.017991 | 0.043071 | 1.259404 | 2.05E-05 | 0.000101 |
| MIR6810 | 0.128273 | 0.369205 | 1.525208 | 1.17E-10 | 2.66E-09 |
| AC108704.2 | 0.096346 | 0.270282 | 1.488171 | 1.02E-19 | 5.33E-17 |
| AC007494.1 | 0.030187 | 0.125188 | 2.052115 | 8.87E-05 | 0.000363 |
| SPDYE19P | 0.030288 | 0.089664 | 1.56579 | 2.43E-08 | 2.84E-07 |
| AL356020.1 | 0.013116 | 0.036847 | 1.490223 | 0.010859 | 0.022953 |
| DUXAP1 | 0.032894 | 0.076914 | 1.225433 | 0.017115 | 0.03372 |
| AC104115.2 | 0.05245 | 0.168659 | 1.685091 | 1.68E-11 | 4.88E-10 |
| RNU6-343P | 0.172849 | 0.455949 | 1.399357 | 6.66E-07 | 5.08E-06 |
| AC093520.2 | 0.045277 | 0.102342 | 1.176564 | 0.000353 | 0.001201 |
| AL450306.1 | 0.14293 | 0.289622 | 1.018861 | 1.49E-11 | 4.44E-10 |
| AC020688.1 | 0.073184 | 0.157941 | 1.10979 | 1.77E-06 | 1.20E-05 |
| AC007728.2 | 0.068774 | 0.147646 | 1.102205 | 1.49E-06 | 1.03E-05 |
| AP003557.1 | 0.019134 | 0.051009 | 1.414578 | 0.002316 | 0.006111 |
| RNA5SP18 | 0.584133 | 1.227266 | 1.071078 | 0.00023 | 0.000829 |
| TATDN2P2 | 0.248168 | 0.582395 | 1.230682 | 2.11E-19 | 9.85E-17 |
| AL031772.1 | 0.013752 | 0.08557 | 2.637426 | 2.87E-09 | 4.36E-08 |
| AC104791.1 | 0.057147 | 0.289148 | 2.339053 | 6.36E-08 | 6.62E-07 |
| AC122129.1 | 0.266172 | 0.578931 | 1.121033 | 3.88E-21 | 5.35E-18 |
| AL591848.1 | 0.041191 | 0.265486 | 2.688243 | 1.14E-08 | 1.47E-07 |
| OSTCP8 | 0.030256 | 0.20534 | 2.762732 | 0.001276 | 0.003663 |
| AC103810.1 | 0.192893 | 0.418229 | 1.116493 | 2.16E-12 | 8.18E-11 |
| AC006141.1 | 0.008509 | 0.028645 | 1.751279 | 0.00093 | 0.00279 |
| IFI44L | 0.899733 | 2.272472 | 1.336693 | 0.000236 | 0.000848 |
| AL138787.2 | 0.153042 | 0.351118 | 1.198026 | 0.006304 | 0.014408 |
| AC020661.3 | 0.039775 | 0.15069 | 1.921632 | 1.39E-08 | 1.74E-07 |
| AC010973.1 | 0.107019 | 0.225828 | 1.077361 | 3.68E-11 | 9.64E-10 |
| NBAT1 | 0.012894 | 0.029689 | 1.20321 | 0.001476 | 0.004154 |
| SPATA46 | 0.036473 | 0.105693 | 1.534967 | 7.87E-16 | 9.40E-14 |
| AC007956.1 | 0.074516 | 0.176562 | 1.244547 | 1.55E-11 | 4.57E-10 |
| AL157838.1 | 0.55343 | 1.193935 | 1.109251 | 9.49E-15 | 7.31E-13 |
| FP325332.1 | 0.020816 | 0.065412 | 1.651858 | 4.22E-06 | 2.56E-05 |
| RNU6-720P | 0.130017 | 0.567368 | 2.12559 | 7.33E-11 | 1.77E-09 |
| OR2L1P | 0.034468 | 0.073259 | 1.087759 | 0.000128 | 0.000499 |
| AC234781.1 | 0.008636 | 0.021006 | 1.282455 | 0.000954 | 0.002851 |
| MCCD1P1 | 0.046379 | 0.107142 | 1.207995 | 0.000104 | 0.000416 |
| E2F3P1 | 0.016034 | 0.069081 | 2.107114 | 0.000284 | 0.000993 |
| IDI2-AS1 | 0.062801 | 0.125627 | 1.000282 | 2.42E-13 | 1.23E-11 |
| TUBB8P2 | 0.036041 | 0.134437 | 1.899241 | 1.39E-11 | 4.18E-10 |
| AC099811.6 | 0.01169 | 0.076335 | 2.707026 | 0.001155 | 0.003361 |
| MIR5692B | 0.512171 | 1.201637 | 1.230304 | 2.11E-12 | 8.03E-11 |
| AL354793.1 | 0.016935 | 0.043554 | 1.362823 | 1.01E-07 | 9.89E-07 |
| AC104134.1 | 0.017599 | 0.038359 | 1.124071 | 8.92E-06 | 4.91E-05 |
| AF230666.2 | 0.039303 | 0.086898 | 1.14468 | 0.000245 | 0.000873 |
| PHBP19 | 0.099808 | 0.212268 | 1.088665 | 1.26E-07 | 1.19E-06 |
| MFSD1P1 | 0.015364 | 0.241653 | 3.975331 | 4.27E-06 | 2.59E-05 |
| AC099550.1 | 0.014712 | 0.052462 | 1.834313 | 0.009018 | 0.019596 |
| TRH | 0.014714 | 0.050763 | 1.78658 | 0.007513 | 0.01675 |
| YPEL5P2 | 0.05083 | 0.167705 | 1.722161 | 1.12E-07 | 1.08E-06 |
| OR51C1P | 2.342774 | 7.085198 | 1.59659 | 2.46E-05 | 0.000119 |
| PARP15 | 0.147999 | 0.353466 | 1.255983 | 2.92E-08 | 3.33E-07 |
| TVP23CP2 | 0.023813 | 0.11335 | 2.250992 | 0.003898 | 0.009559 |
| AC068790.5 | 0.303327 | 0.635526 | 1.067075 | 6.61E-06 | 3.78E-05 |
| AL590644.1 | 0.006121 | 0.014887 | 1.282204 | 0.004198 | 0.010185 |
| AC006978.2 | 0.058371 | 0.126655 | 1.117579 | 4.09E-06 | 2.50E-05 |
| AL133330.1 | 0.11759 | 0.28458 | 1.275063 | 4.17E-05 | 0.000187 |
| RNA5SP123 | 0.072288 | 0.743131 | 3.361794 | 5.64E-09 | 7.87E-08 |
| ELOCP21 | 0.071067 | 0.144777 | 1.026579 | 0.000159 | 0.000603 |
| AC005842.1 | 0.055824 | 0.144033 | 1.367432 | 1.21E-06 | 8.51E-06 |
| RNU6-595P | 0.193313 | 0.566692 | 1.551625 | 4.80E-05 | 0.000212 |
| CNGB3 | 0.016024 | 0.038667 | 1.27085 | 1.78E-08 | 2.17E-07 |
| CLEC12B | 0.006789 | 0.015109 | 1.154018 | 4.09E-07 | 3.32E-06 |
| AC130456.7 | 0.047071 | 0.098888 | 1.070949 | 1.86E-08 | 2.25E-07 |
| FTH1P24 | 0.026563 | 0.310264 | 3.545979 | 1.56E-05 | 7.97E-05 |
| FNDC1 | 0.256101 | 0.574601 | 1.165847 | 1.13E-05 | 6.04E-05 |
| AC016586.1 | 0.239073 | 0.480588 | 1.00735 | 1.27E-15 | 1.41E-13 |
| TRIM36-IT1 | 0.042425 | 0.112617 | 1.408454 | 3.57E-06 | 2.22E-05 |
| AL592148.1 | 0.103289 | 0.568501 | 2.460479 | 9.92E-08 | 9.74E-07 |
| TAS2R64P | 0.023099 | 0.051731 | 1.163219 | 6.65E-10 | 1.22E-08 |
| AL355377.1 | 0.017573 | 0.056112 | 1.674949 | 1.96E-07 | 1.75E-06 |
| GK-AS1 | 0.061613 | 0.155726 | 1.337699 | 2.42E-15 | 2.31E-13 |
| AL162431.3 | 0.040934 | 0.125853 | 1.620376 | 5.88E-08 | 6.18E-07 |
| AL592301.1 | 0.042782 | 0.098557 | 1.20397 | 0.001028 | 0.003038 |
| AC109992.2 | 0.024048 | 0.064436 | 1.421964 | 1.88E-09 | 3.03E-08 |
| AC087477.6 | 0.016459 | 0.055944 | 1.765072 | 1.09E-06 | 7.79E-06 |
| AC011479.3 | 0.029553 | 0.089078 | 1.591763 | 0.000339 | 0.00116 |
| AL358334.3 | 0.021157 | 0.049459 | 1.225098 | 3.50E-06 | 2.18E-05 |
| AC027018.1 | 0.011875 | 0.157774 | 3.731813 | 3.44E-08 | 3.85E-07 |
| AC245884.11 | 0.007032 | 0.01633 | 1.215607 | 6.93E-05 | 0.000292 |
| AC000120.1 | 0.061252 | 0.19065 | 1.638093 | 3.26E-18 | 9.65E-16 |
| BOLA2P2 | 0.135861 | 0.303746 | 1.160732 | 4.26E-14 | 2.78E-12 |
| LINC02097 | 0.013172 | 0.035864 | 1.445056 | 6.97E-05 | 0.000293 |
| AC069257.1 | 0.029242 | 0.191963 | 2.714717 | 8.39E-09 | 1.12E-07 |
| AF131215.4 | 0.034156 | 0.483541 | 3.823445 | 5.87E-13 | 2.69E-11 |
| AC010319.1 | 0.062524 | 0.142037 | 1.183781 | 1.42E-05 | 7.36E-05 |
| LDHAL6CP | 0.010129 | 0.021089 | 1.057905 | 0.019196 | 0.037124 |
| AC009533.2 | 0.031275 | 0.06586 | 1.074378 | 7.29E-07 | 5.50E-06 |
| AC084117.1 | 0.409979 | 0.834302 | 1.025018 | 6.96E-09 | 9.48E-08 |
| DDX3P1 | 0.018364 | 0.211392 | 3.524983 | 6.76E-06 | 3.86E-05 |
| AC073957.3 | 0.281987 | 0.571075 | 1.018052 | 1.44E-18 | 4.98E-16 |
| MIR1250 | 0.075953 | 0.212557 | 1.484669 | 1.37E-06 | 9.51E-06 |
| AC096536.1 | 0.047341 | 0.102309 | 1.111786 | 8.57E-07 | 6.34E-06 |
| AC090971.2 | 0.029383 | 0.076239 | 1.375535 | 0.006328 | 0.014452 |
| AL158152.1 | 0.086638 | 0.242504 | 1.484938 | 3.05E-09 | 4.59E-08 |
| AC083805.2 | 0.047137 | 0.105472 | 1.161919 | 0.004052 | 0.009881 |
| AC010261.2 | 0.025109 | 0.092959 | 1.888388 | 1.22E-07 | 1.16E-06 |
| RPL18AP2 | 0.032471 | 0.06971 | 1.102221 | 0.006469 | 0.014732 |
| PCDH17 | 1.127987 | 2.40275 | 1.090936 | 0.00221 | 0.00587 |
| RNU6-1005P | 0.089844 | 0.285729 | 1.669161 | 4.82E-06 | 2.87E-05 |
| AC004594.1 | 0.05358 | 0.18023 | 1.750069 | 3.38E-11 | 8.91E-10 |
| AC007040.1 | 0.024976 | 0.05319 | 1.090631 | 4.30E-08 | 4.69E-07 |
| AC138951.1 | 0.015305 | 0.040311 | 1.397114 | 5.95E-06 | 3.46E-05 |
| ATP8B5P | 0.060036 | 0.139324 | 1.214541 | 1.36E-09 | 2.30E-08 |
| AC089998.3 | 0.040233 | 0.171213 | 2.089329 | 5.96E-10 | 1.11E-08 |
| RPS15AP16 | 0.09475 | 0.324595 | 1.776441 | 0.001705 | 0.004696 |
| AC013394.1 | 0.102323 | 0.283616 | 1.470805 | 3.62E-10 | 7.16E-09 |
| RNU6-1301P | 0.143426 | 0.388388 | 1.437194 | 0.002914 | 0.007446 |
| AL589743.4 | 0.122862 | 0.410638 | 1.740829 | 7.36E-10 | 1.34E-08 |
| AC007622.2 | 0.088983 | 0.192613 | 1.114103 | 4.05E-15 | 3.55E-13 |
| CATIP-AS2 | 0.082673 | 0.173891 | 1.0727 | 5.46E-05 | 0.000237 |
| DDX11L10 | 0.01817 | 0.037387 | 1.040949 | 0.002169 | 0.005771 |
| AL139317.4 | 0.049864 | 0.221645 | 2.152182 | 4.59E-11 | 1.17E-09 |
| AP001208.2 | 0.296886 | 0.797348 | 1.425299 | 6.49E-08 | 6.73E-07 |
| AC114982.1 | 0.030356 | 0.10111 | 1.735874 | 4.58E-06 | 2.75E-05 |
| AP000692.1 | 0.309545 | 0.667039 | 1.107621 | 7.50E-14 | 4.50E-12 |
| RPL21P32 | 0.043041 | 0.10607 | 1.301237 | 9.26E-06 | 5.07E-05 |
| AC008147.1 | 0.051595 | 0.117903 | 1.192295 | 0.006707 | 0.015183 |
| RN7SL809P | 0.186308 | 0.59202 | 1.667957 | 5.73E-14 | 3.61E-12 |
| AC005220.1 | 0.009973 | 0.026707 | 1.421164 | 0.000956 | 0.002854 |
| RNU6-828P | 0.09965 | 0.274184 | 1.460207 | 8.74E-07 | 6.44E-06 |
| AC005726.2 | 0.022835 | 0.053394 | 1.225431 | 1.88E-09 | 3.02E-08 |
| BNIP3P39 | 0.100577 | 0.204463 | 1.023535 | 7.35E-10 | 1.34E-08 |
| AC016722.2 | 0.121068 | 0.271986 | 1.167714 | 1.13E-22 | 5.18E-19 |
| RN7SL338P | 0.053031 | 0.294025 | 2.471026 | 2.59E-05 | 0.000124 |
| SNORA7B | 0.257424 | 0.851615 | 1.726057 | 1.98E-05 | 9.83E-05 |
| IGHV3-49 | 3.98586 | 8.446214 | 1.083414 | 0.004576 | 0.010975 |
| TCEAL3-AS1 | 0.063022 | 0.177853 | 1.496745 | 1.18E-05 | 6.29E-05 |
| AC006518.2 | 0.047059 | 0.140802 | 1.581125 | 1.35E-06 | 9.39E-06 |
| RNU5F-1 | 0.155132 | 0.389252 | 1.327204 | 5.89E-05 | 0.000253 |
| AC118465.1 | 0.041958 | 0.156279 | 1.897106 | 0.00033 | 0.001133 |
| LINC00624 | 0.068242 | 0.146841 | 1.105524 | 3.92E-11 | 1.02E-09 |
| AL162430.2 | 0.149148 | 0.300531 | 1.010769 | 7.98E-05 | 0.00033 |
| LCN1P1 | 0.035413 | 0.091728 | 1.373068 | 0.000772 | 0.002373 |
| UBE2V1P1 | 0.038571 | 0.114698 | 1.572261 | 1.87E-07 | 1.69E-06 |
| AL096803.1 | 0.267126 | 0.741835 | 1.473577 | 0.000401 | 0.001342 |
| CTLA4 | 0.50679 | 1.132127 | 1.159577 | 2.55E-05 | 0.000123 |
| MIR1302-8 | 0.061775 | 0.193156 | 1.644674 | 9.38E-06 | 5.12E-05 |
| AC092364.2 | 0.039642 | 0.101705 | 1.359305 | 0.000275 | 0.000965 |
| AC073283.1 | 0.019155 | 0.051699 | 1.432431 | 3.80E-07 | 3.11E-06 |
| AC006262.1 | 0.022188 | 0.051529 | 1.215621 | 0.021184 | 0.040288 |
| AL626787.1 | 0.041153 | 0.111656 | 1.439979 | 0.009235 | 0.019989 |
| AC108206.1 | 0.028829 | 0.079168 | 1.457392 | 0.000351 | 0.001196 |
| AP005432.1 | 0.020492 | 0.055735 | 1.443537 | 0.02016 | 0.038648 |
| AC006017.1 | 0.175642 | 0.35164 | 1.001461 | 3.34E-09 | 4.97E-08 |
| AL049637.1 | 0.019956 | 0.302807 | 3.923479 | 1.17E-05 | 6.23E-05 |
| OAS3 | 3.78554 | 8.234585 | 1.121197 | 2.05E-10 | 4.37E-09 |
| RPL32P1 | 0.105414 | 0.30422 | 1.52905 | 3.03E-09 | 4.56E-08 |
| AC073349.2 | 0.239297 | 0.613387 | 1.357998 | 5.20E-15 | 4.40E-13 |
| LARP1P1 | 0.159876 | 0.364006 | 1.187004 | 0.001259 | 0.003623 |
| MARCKSL1P1 | 0.070805 | 0.21521 | 1.603818 | 2.12E-12 | 8.06E-11 |
| NRBF2P5 | 0.127861 | 0.381844 | 1.578409 | 1.93E-15 | 1.96E-13 |
| AC116424.1 | 0.014696 | 0.049044 | 1.738667 | 1.06E-05 | 5.69E-05 |
| AC108449.1 | 0.020201 | 0.049753 | 1.30038 | 2.70E-05 | 0.000129 |
| XIAPP2 | 0.011427 | 0.048433 | 2.083569 | 0.00486 | 0.011552 |
| RN7SL473P | 0.045112 | 0.433753 | 3.265298 | 4.45E-12 | 1.54E-10 |
| AC026401.2 | 0.027159 | 0.086234 | 1.666828 | 2.06E-06 | 1.37E-05 |
| SNORA23 | 0.175512 | 5.852959 | 5.059528 | 0.006815 | 0.0154 |
| AC145285.5 | 0.017269 | 0.03502 | 1.02001 | 0.018411 | 0.035833 |
| GSN-AS1 | 0.045773 | 0.133172 | 1.540736 | 1.44E-07 | 1.34E-06 |
| POU5F2 | 0.008124 | 0.072173 | 3.151253 | 3.76E-05 | 0.000172 |
| AC027514.2 | 0.147423 | 0.380741 | 1.368846 | 3.40E-19 | 1.51E-16 |
| PCCA-AS1 | 0.047195 | 0.415796 | 3.139172 | 2.23E-10 | 4.70E-09 |
| LINC00943 | 0.008335 | 0.020169 | 1.274912 | 0.013164 | 0.02703 |
| Z97192.3 | 0.015251 | 0.041582 | 1.447111 | 0.013137 | 0.026986 |
| SNORD3B-2 | 0.085653 | 0.175959 | 1.038665 | 0.023858 | 0.044613 |
| AC073052.1 | 0.116538 | 0.27637 | 1.245798 | 4.14E-08 | 4.53E-07 |
| AL049869.3 | 0.290567 | 0.963683 | 1.729687 | 1.18E-17 | 2.88E-15 |
| AC006058.4 | 0.012609 | 0.047562 | 1.915353 | 0.003723 | 0.009194 |
| CLIP1-AS1 | 0.018376 | 0.109457 | 2.574444 | 7.63E-05 | 0.000318 |
| AC002366.1 | 0.009531 | 0.099382 | 3.382218 | 4.20E-05 | 0.000189 |
| SNORD116-26 | 0.152329 | 0.535483 | 1.813644 | 1.70E-05 | 8.62E-05 |
| AC118344.1 | 0.097471 | 0.257075 | 1.399139 | 1.46E-16 | 2.36E-14 |
| AC133552.3 | 0.019248 | 0.040692 | 1.080041 | 2.79E-05 | 0.000132 |
| AC105345.2 | 0.016478 | 0.045795 | 1.474624 | 1.37E-06 | 9.51E-06 |
| AC104984.3 | 0.030057 | 0.101984 | 1.762566 | 0.000218 | 0.000791 |
| FNTAP2 | 0.025695 | 0.102939 | 2.00225 | 0.021169 | 0.040262 |
| AC067940.1 | 0.049933 | 0.259528 | 2.377829 | 0.000339 | 0.00116 |
| SLC35E1P1 | 0.128394 | 0.259703 | 1.016291 | 8.52E-08 | 8.54E-07 |
| MARK2P8 | 0.022946 | 0.236813 | 3.36741 | 4.29E-09 | 6.20E-08 |
| AL031667.2 | 0.054006 | 0.292199 | 2.435748 | 3.65E-07 | 3.02E-06 |
| RNY1P16 | 0.344243 | 0.820056 | 1.252296 | 6.86E-07 | 5.22E-06 |
| COL18A1-AS1 | 0.002967 | 0.035509 | 3.580899 | 1.14E-05 | 6.10E-05 |
| AJ009632.2 | 0.013782 | 0.028286 | 1.037268 | 1.29E-05 | 6.74E-05 |
| MALAT1 | 18.66623 | 94.0444 | 2.332912 | 1.63E-15 | 1.72E-13 |
| AC026362.2 | 0.003983 | 0.026792 | 2.749827 | 0.010209 | 0.021779 |
| ITGB5-AS1 | 0.036995 | 0.123296 | 1.736721 | 2.79E-06 | 1.79E-05 |
| AC007546.1 | 0.134136 | 0.271843 | 1.019081 | 1.04E-06 | 7.50E-06 |
| AC018752.1 | 0.549324 | 1.362837 | 1.310883 | 2.58E-05 | 0.000124 |
| AC025871.1 | 0.019766 | 0.051671 | 1.386342 | 0.019362 | 0.037383 |
| RPS26P41 | 0.144876 | 0.618653 | 2.094311 | 5.17E-05 | 0.000226 |
| KRR1P1 | 0.204514 | 0.532328 | 1.380118 | 2.24E-13 | 1.16E-11 |
| AL136984.1 | 0.015037 | 0.091984 | 2.612866 | 2.63E-05 | 0.000126 |
| AL592430.1 | 0.152509 | 0.401378 | 1.396071 | 3.25E-12 | 1.17E-10 |
| GAPDHP33 | 0.026026 | 0.055936 | 1.10384 | 0.027235 | 0.049808 |
| IGHJ3P | 0.650636 | 1.83705 | 1.49747 | 0.000241 | 0.000862 |
| RNU6-807P | 0.181641 | 0.411902 | 1.181214 | 6.23E-10 | 1.15E-08 |
| MIR553 | 0.18691 | 0.451053 | 1.270955 | 0.000188 | 0.000697 |
| AC008731.1 | 0.129785 | 0.312646 | 1.268402 | 2.12E-12 | 8.07E-11 |
| TEDDM1 | 0.025488 | 0.05192 | 1.026472 | 2.12E-07 | 1.87E-06 |
| PSMD10P1 | 0.123299 | 0.974965 | 2.983195 | 2.01E-05 | 9.96E-05 |
| SHANK2-AS1 | 0.009092 | 0.105629 | 3.538249 | 1.16E-05 | 6.17E-05 |
| RNU6-431P | 0.178827 | 0.40992 | 1.196777 | 5.34E-11 | 1.33E-09 |
| AL353748.2 | 0.088078 | 0.365966 | 2.054851 | 1.30E-12 | 5.32E-11 |
| AC008635.1 | 0.05582 | 0.11959 | 1.099237 | 1.63E-09 | 2.67E-08 |
| AL078581.3 | 0.069908 | 0.191097 | 1.450782 | 6.45E-18 | 1.76E-15 |
| AC010627.1 | 0.048622 | 0.225404 | 2.212835 | 1.13E-06 | 8.08E-06 |
| AP003392.2 | 0.844374 | 1.772742 | 1.070029 | 4.81E-19 | 1.95E-16 |
| AC009093.7 | 0.009822 | 0.080003 | 3.025918 | 0.001859 | 0.005059 |
| AP003170.1 | 0.091148 | 0.201801 | 1.146649 | 7.37E-14 | 4.45E-12 |
| AC007114.2 | 0.038531 | 0.130295 | 1.75768 | 7.18E-14 | 4.38E-12 |
| SRRM1P3 | 0.010972 | 0.044497 | 2.01982 | 6.47E-05 | 0.000274 |
| AC024940.3 | 0.043895 | 0.170566 | 1.958194 | 4.17E-05 | 0.000187 |
| AC005546.1 | 0.064597 | 0.157531 | 1.286109 | 6.16E-14 | 3.84E-12 |
| AL024474.1 | 0.018811 | 0.081131 | 2.108645 | 4.16E-05 | 0.000187 |
| MME-AS1 | 0.049061 | 0.131049 | 1.417448 | 0.000112 | 0.000443 |
| MTCO3P29 | 0.030788 | 0.086569 | 1.491496 | 8.45E-08 | 8.48E-07 |
| VIL1 | 0.031097 | 0.10317 | 1.730166 | 0.005655 | 0.013124 |
| AL590282.1 | 0.052565 | 0.129698 | 1.302979 | 1.59E-12 | 6.31E-11 |
| SNORA71A | 0.781337 | 2.9396 | 1.911602 | 7.90E-13 | 3.44E-11 |
| FCRL1 | 0.077981 | 0.219884 | 1.495551 | 0.001504 | 0.004224 |
| AC113382.2 | 0.024309 | 0.051928 | 1.09501 | 0.000709 | 0.002206 |
| SNORD14E | 4.342184 | 9.40895 | 1.115613 | 0.001764 | 0.004839 |
| CALM2P2 | 0.763941 | 1.705409 | 1.158585 | 5.85E-21 | 6.72E-18 |
| MEP1B | 0.008094 | 0.024065 | 1.572106 | 1.29E-05 | 6.78E-05 |
| AC013643.2 | 0.026387 | 0.057842 | 1.132293 | 0.002457 | 0.006417 |
| AC132219.1 | 0.052256 | 0.109528 | 1.067618 | 6.64E-11 | 1.63E-09 |
| AC018462.1 | 0.061401 | 0.139098 | 1.179769 | 3.15E-06 | 1.98E-05 |
| AC104076.2 | 0.059362 | 0.154848 | 1.383237 | 0.000133 | 0.000515 |
| AC097382.2 | 0.018096 | 0.036882 | 1.027252 | 2.89E-17 | 6.13E-15 |
| AC010761.3 | 0.194496 | 0.585915 | 1.590951 | 3.06E-16 | 4.18E-14 |
| AL133227.1 | 0.119291 | 0.270638 | 1.181881 | 5.43E-10 | 1.02E-08 |
| SLC25A14P1 | 0.011089 | 0.038301 | 1.788182 | 1.44E-05 | 7.43E-05 |
| AC022558.1 | 0.131707 | 0.300041 | 1.187826 | 1.83E-18 | 5.94E-16 |
| ANKRD26P4 | 0.012019 | 0.026251 | 1.127016 | 1.29E-06 | 9.02E-06 |
| IGHV3-73 | 2.411601 | 8.069623 | 1.74251 | 0.001457 | 0.00411 |
| RAB11FIP1P1 | 0.421668 | 1.018425 | 1.272159 | 1.38E-20 | 1.32E-17 |
| AC078845.1 | 0.012685 | 0.028663 | 1.176036 | 1.28E-05 | 6.70E-05 |
| TAS2R50 | 0.013671 | 0.052354 | 1.937146 | 6.51E-05 | 0.000276 |
| AL049840.6 | 0.087117 | 0.258829 | 1.570982 | 6.57E-10 | 1.21E-08 |
| AL451054.1 | 0.016175 | 0.049599 | 1.616494 | 0.002951 | 0.00753 |
| AC024145.1 | 0.26736 | 0.547577 | 1.034279 | 4.06E-05 | 0.000183 |
| PM20D1 | 0.037486 | 0.122627 | 1.709853 | 0.000684 | 0.002139 |
| CCDC200 | 0.104809 | 0.209972 | 1.002434 | 1.02E-06 | 7.41E-06 |
| RNU6-190P | 0.127283 | 0.629214 | 2.305516 | 6.81E-07 | 5.18E-06 |
| AC104129.1 | 0.001704 | 0.011078 | 2.700666 | 7.98E-07 | 5.95E-06 |
| RN7SL145P | 0.182699 | 0.39508 | 1.112676 | 4.37E-10 | 8.42E-09 |
| AP000919.2 | 0.011571 | 0.042792 | 1.886792 | 1.61E-05 | 8.23E-05 |
| RN7SL333P | 0.180167 | 0.393687 | 1.127716 | 6.39E-12 | 2.10E-10 |
| AC116903.2 | 0.008717 | 0.024886 | 1.513413 | 0.000196 | 0.000723 |
| AC092802.3 | 0.032719 | 0.115589 | 1.820782 | 0.001766 | 0.004843 |
| AC093110.1 | 0.24839 | 0.692524 | 1.47926 | 4.91E-18 | 1.39E-15 |
| GSDMB | 1.276474 | 2.615135 | 1.034721 | 4.78E-06 | 2.86E-05 |
| ERVFRD-3 | 0.017369 | 0.03973 | 1.19375 | 1.17E-05 | 6.23E-05 |
| AL583832.1 | 0.073232 | 0.252162 | 1.783803 | 1.58E-15 | 1.70E-13 |
| LINC02408 | 0.015305 | 0.032143 | 1.07048 | 2.31E-05 | 0.000112 |
| AL355472.3 | 0.147369 | 0.308324 | 1.065014 | 7.53E-10 | 1.37E-08 |
| FTX | 0.308586 | 0.645239 | 1.064159 | 1.05E-16 | 1.77E-14 |
| AC068790.6 | 0.101345 | 0.258595 | 1.351419 | 2.70E-09 | 4.13E-08 |
| AL031595.3 | 0.018274 | 0.040976 | 1.165014 | 5.25E-12 | 1.77E-10 |
| PATE1 | 0.090667 | 0.702161 | 2.95315 | 0.005053 | 0.011933 |
| RPL7P39 | 0.026353 | 0.063102 | 1.25973 | 2.35E-05 | 0.000114 |
| BCRP1 | 0.023879 | 0.065946 | 1.465572 | 0.003795 | 0.009342 |
| NIP7P3 | 0.013143 | 0.271489 | 4.368497 | 9.95E-07 | 7.22E-06 |
| AC009948.5 | 0.005645 | 0.040972 | 2.859618 | 0.002502 | 0.006516 |
| AP000763.3 | 0.050984 | 0.105367 | 1.047298 | 6.93E-06 | 3.94E-05 |
| OR7E13P | 0.02787 | 0.107601 | 1.948886 | 0.000641 | 0.002021 |
| AC004223.2 | 0.106025 | 0.240774 | 1.183278 | 0.004422 | 0.010649 |
| MIR6769A | 0.195115 | 0.406191 | 1.057834 | 8.61E-08 | 8.63E-07 |
| LUCAT1 | 0.01835 | 0.041335 | 1.171535 | 0.000729 | 0.00226 |
| AC010761.4 | 0.214358 | 0.446663 | 1.059166 | 5.56E-18 | 1.53E-15 |
| AC007728.3 | 0.013236 | 0.049503 | 1.903018 | 6.84E-05 | 0.000288 |
| MIR4668 | 0.169952 | 0.406816 | 1.25925 | 2.54E-06 | 1.65E-05 |
| AL031770.1 | 0.027262 | 0.07911 | 1.536957 | 0.003169 | 0.008005 |
| SNRPGP15 | 0.509204 | 1.083742 | 1.089705 | 2.58E-13 | 1.30E-11 |
| KIF28P | 0.182815 | 0.378581 | 1.050219 | 5.33E-11 | 1.33E-09 |
| GRM5 | 0.000992 | 0.002354 | 1.246441 | 1.84E-05 | 9.23E-05 |
| MRAP | 0.018181 | 0.044247 | 1.283168 | 0.014782 | 0.029772 |
| AC025180.1 | 0.020443 | 0.065568 | 1.681375 | 0.007537 | 0.0168 |
| SRP72P2 | 0.03539 | 0.110361 | 1.640804 | 1.22E-06 | 8.58E-06 |
| AC244093.2 | 0.157067 | 0.520996 | 1.729892 | 0.004287 | 0.010369 |
| AC067945.1 | 0.041807 | 0.329838 | 2.979925 | 0.000651 | 0.002048 |
| ITGA6-AS1 | 0.081637 | 0.213932 | 1.389851 | 0.019719 | 0.03796 |
| GCNT1P1 | 0.014437 | 0.043034 | 1.575749 | 1.23E-06 | 8.65E-06 |
| RAP1BP1 | 0.049152 | 0.152805 | 1.636373 | 0.005256 | 0.01234 |
| AC011284.1 | 0.067097 | 0.213974 | 1.673121 | 0.001559 | 0.004351 |
| AC108063.1 | 0.065731 | 0.141985 | 1.111103 | 0.000847 | 0.002573 |
| SHC1P2 | 0.01183 | 0.024899 | 1.073663 | 6.27E-06 | 3.62E-05 |
| AL135999.2 | 0.034772 | 0.074446 | 1.098263 | 2.50E-06 | 1.63E-05 |
| AC092916.2 | 0.035205 | 0.071064 | 1.013347 | 0.001285 | 0.003687 |
| MIR27B | 0.658041 | 1.323362 | 1.007957 | 5.26E-05 | 0.00023 |
| AC091544.2 | 0.203428 | 0.798008 | 1.971885 | 1.08E-17 | 2.67E-15 |
| AC084871.1 | 0.106304 | 0.412684 | 1.956845 | 1.91E-10 | 4.11E-09 |
| AC124312.4 | 0.302462 | 1.928334 | 2.672529 | 6.27E-08 | 6.54E-07 |
| RNA5SP132 | 0.072808 | 0.160716 | 1.142346 | 3.36E-05 | 0.000156 |
| AC090519.2 | 0.023249 | 0.123079 | 2.404328 | 0.000758 | 0.002339 |
| RNU6-890P | 0.109887 | 0.291391 | 1.406933 | 2.14E-06 | 1.42E-05 |
| AL354726.1 | 0.03101 | 0.195633 | 2.657368 | 6.46E-08 | 6.71E-07 |
| AC008277.1 | 0.015628 | 0.075433 | 2.271024 | 1.47E-05 | 7.57E-05 |
| HLA-Z | 0.377182 | 0.818102 | 1.117019 | 0.004929 | 0.011682 |
| AC108693.1 | 0.023171 | 0.100804 | 2.121198 | 2.30E-06 | 1.51E-05 |
| AC008781.2 | 0.00672 | 0.074552 | 3.471786 | 0.000195 | 0.00072 |
| AL049840.1 | 0.784223 | 1.709469 | 1.124212 | 8.72E-22 | 1.60E-18 |
| ANKRD36C | 0.152091 | 0.37643 | 1.307453 | 1.22E-17 | 2.95E-15 |
| IGHV4-34 | 6.313966 | 17.46185 | 1.467588 | 0.022484 | 0.042421 |
| AC084880.3 | 0.040629 | 0.189676 | 2.222963 | 0.002294 | 0.006064 |
| PAGE4 | 8.296081 | 3.859942 | -1.10385 | 9.21E-07 | 6.75E-06 |
| AC231657.2 | 0.083672 | 0.182404 | 1.124318 | 7.61E-06 | 4.27E-05 |
| C1QTNF3 | 2.733322 | 7.128064 | 1.382855 | 0.006218 | 0.014231 |
| TMPRSS11CP | 0.100887 | 0.29815 | 1.5633 | 0.0006 | 0.001908 |
| RN7SL566P | 0.256821 | 0.541468 | 1.076112 | 1.38E-09 | 2.32E-08 |
| C9orf131 | 0.011338 | 0.051438 | 2.181632 | 7.81E-12 | 2.53E-10 |
| AL035658.1 | 0.043202 | 0.09119 | 1.077765 | 1.21E-09 | 2.07E-08 |
| MYO18B | 0.122635 | 0.279291 | 1.187399 | 0.003739 | 0.009223 |
| AC060234.2 | 0.017962 | 0.04783 | 1.413001 | 8.07E-07 | 6.01E-06 |
| AC138409.2 | 0.258159 | 0.54862 | 1.087548 | 3.48E-13 | 1.68E-11 |
| AC092574.2 | 0.055995 | 0.313785 | 2.486405 | 5.00E-12 | 1.70E-10 |
| AC024267.3 | 0.223208 | 0.49545 | 1.150354 | 1.04E-16 | 1.77E-14 |
| ETF1P1 | 0.025422 | 0.055426 | 1.124472 | 0.000544 | 0.001754 |
| MTND4P15 | 0.014284 | 0.072489 | 2.343318 | 1.76E-12 | 6.87E-11 |
| AC006518.1 | 0.01218 | 0.040435 | 1.731068 | 3.53E-06 | 2.20E-05 |
| AC009120.3 | 0.386647 | 0.818536 | 1.08203 | 3.10E-17 | 6.38E-15 |
| AC120349.3 | 0.020988 | 0.177028 | 3.076354 | 3.45E-07 | 2.87E-06 |
| LIF | 1.398777 | 3.164816 | 1.177955 | 2.35E-05 | 0.000114 |
| TAS2R43 | 0.029042 | 0.061707 | 1.087285 | 2.08E-06 | 1.38E-05 |
| EGFR-AS1 | 0.051383 | 0.10354 | 1.010827 | 1.28E-05 | 6.70E-05 |
| SLC4A9 | 0.020752 | 0.050032 | 1.269575 | 0.004768 | 0.011365 |
| AC026310.3 | 0.004571 | 0.010108 | 1.145079 | 0.004137 | 0.010063 |
| AL034345.1 | 0.510826 | 1.059894 | 1.053017 | 0.001228 | 0.003547 |
| AL512310.11 | 0.274259 | 0.688584 | 1.328096 | 0.000212 | 0.000772 |
| AC008894.2 | 0.021248 | 0.048371 | 1.186858 | 3.96E-15 | 3.47E-13 |
| RF02119 | 0.22325 | 0.527925 | 1.241672 | 1.90E-05 | 9.48E-05 |
| SRMP2 | 0.014971 | 0.109322 | 2.868333 | 0.003206 | 0.008088 |
| AL158068.2 | 0.017298 | 0.038333 | 1.148004 | 0.000391 | 0.001312 |
| AC091588.1 | 0.03413 | 0.196875 | 2.528169 | 6.64E-05 | 0.000281 |
| REG4 | 7.986156 | 1.994934 | -2.00116 | 0.000245 | 0.000873 |
| TPM3P4 | 0.05792 | 0.133314 | 1.202695 | 0.000273 | 0.000962 |
| AC073592.3 | 0.011298 | 0.040177 | 1.830277 | 3.67E-09 | 5.41E-08 |
| SNORA15B-2 | 0.098888 | 0.26396 | 1.416451 | 7.89E-07 | 5.89E-06 |
| AC091959.2 | 0.049055 | 0.123543 | 1.332549 | 0.000396 | 0.001327 |
| RN7SL535P | 0.065471 | 0.154528 | 1.238954 | 3.04E-08 | 3.44E-07 |
| BNIP3P26 | 0.045459 | 0.128333 | 1.497253 | 7.69E-11 | 1.84E-09 |
| MIR210 | 0.315592 | 0.643532 | 1.027952 | 1.48E-06 | 1.02E-05 |
| KRT8P26 | 0.028044 | 0.082661 | 1.559517 | 1.26E-05 | 6.64E-05 |
| AL138789.1 | 0.020797 | 0.052045 | 1.323344 | 2.95E-05 | 0.000139 |
| PATE4 | 0.018666 | 0.137047 | 2.876215 | 0.000596 | 0.001896 |
| IQSEC3P1 | 0.019986 | 0.044355 | 1.150079 | 0.000286 | 0.001 |
| SYF2P2 | 0.016065 | 0.088973 | 2.469444 | 7.26E-06 | 4.10E-05 |
| RNU1-103P | 0.268639 | 0.674017 | 1.327115 | 5.46E-10 | 1.03E-08 |
| RNU6-1157P | 0.302398 | 0.820361 | 1.439812 | 1.59E-15 | 1.70E-13 |
| AC007787.2 | 0.238979 | 0.671093 | 1.489632 | 6.69E-19 | 2.53E-16 |
| AC007216.4 | 0.22228 | 0.450115 | 1.017914 | 8.11E-18 | 2.15E-15 |
| CXCL8 | 2.466852 | 5.316091 | 1.107695 | 0.002379 | 0.006252 |
| HSPE1P26 | 0.067992 | 0.397029 | 2.545817 | 2.01E-10 | 4.31E-09 |
| AC008742.1 | 0.011478 | 0.030568 | 1.413155 | 0.000344 | 0.001175 |
| NUDT19P5 | 0.042084 | 0.103581 | 1.299414 | 0.00033 | 0.001134 |
| SNORD67 | 0.083587 | 0.574981 | 2.782168 | 3.85E-06 | 2.37E-05 |
| AC025188.1 | 0.046164 | 0.129914 | 1.492721 | 2.34E-10 | 4.88E-09 |
| SNORD15B | 0.671652 | 9.536358 | 3.827653 | 5.79E-05 | 0.00025 |
| SUGT1P2 | 0.04224 | 0.105565 | 1.321461 | 2.14E-08 | 2.54E-07 |
| AL512791.1 | 0.473401 | 1.093187 | 1.207406 | 1.95E-16 | 2.93E-14 |
| AP000925.1 | 0.027543 | 0.099683 | 1.855683 | 1.87E-08 | 2.27E-07 |
| AC068397.1 | 0.023547 | 0.111323 | 2.241109 | 0.02151 | 0.040829 |
| AP003170.3 | 0.256109 | 0.528911 | 1.046268 | 2.15E-10 | 4.56E-09 |
| AL445490.1 | 0.044539 | 0.229045 | 2.362498 | 2.16E-12 | 8.18E-11 |
| PHACTR2P1 | 0.021419 | 0.070154 | 1.711608 | 4.55E-06 | 2.73E-05 |
| AP003072.5 | 0.015327 | 0.046902 | 1.613605 | 2.82E-06 | 1.81E-05 |
| AC002401.3 | 0.032866 | 0.090213 | 1.45671 | 0.000145 | 0.000556 |
| RNA5SP345 | 0.096307 | 0.197779 | 1.038174 | 2.18E-05 | 0.000107 |
| RN7SL851P | 0.044087 | 0.128105 | 1.538902 | 3.00E-07 | 2.54E-06 |
| AC087286.4 | 0.018844 | 0.222787 | 3.563493 | 3.92E-05 | 0.000178 |
| AC023855.1 | 0.022455 | 0.087193 | 1.957208 | 5.44E-07 | 4.26E-06 |
| AC127024.3 | 0.041566 | 0.129138 | 1.635442 | 0.002439 | 0.006384 |
| AL359922.3 | 0.012017 | 0.033568 | 1.481981 | 0.000447 | 0.001473 |
| AC009303.2 | 0.006429 | 0.029979 | 2.22125 | 0.019255 | 0.037219 |
| AC091117.2 | 0.082451 | 0.611324 | 2.89032 | 8.31E-08 | 8.36E-07 |
| AC100827.4 | 0.014913 | 0.039577 | 1.408073 | 4.15E-08 | 4.54E-07 |
| AC027763.1 | 0.069022 | 0.183092 | 1.407438 | 3.68E-05 | 0.000168 |
| KHDC1L | 0.39354 | 0.119812 | -1.71573 | 0.011077 | 0.023337 |
| AP001350.1 | 0.034258 | 0.081823 | 1.256045 | 2.39E-14 | 1.66E-12 |
| TMEM75 | 0.021778 | 0.317701 | 3.866694 | 3.18E-08 | 3.59E-07 |
| AC018644.1 | 0.058534 | 0.153023 | 1.386394 | 1.52E-17 | 3.53E-15 |
| SNORA54 | 0.14242 | 0.627814 | 2.140183 | 5.64E-05 | 0.000244 |
| PFN1P2 | 0.10387 | 0.219044 | 1.07644 | 1.75E-14 | 1.26E-12 |
| AL138895.2 | 0.077505 | 0.260071 | 1.746544 | 0.007122 | 0.015997 |
| AC134407.1 | 0.069029 | 0.153663 | 1.154498 | 7.38E-06 | 4.16E-05 |
| AL353768.1 | 0.026649 | 0.07625 | 1.516663 | 3.40E-09 | 5.05E-08 |
| RN7SKP16 | 0.242335 | 0.523178 | 1.110302 | 1.91E-11 | 5.47E-10 |
| AC005540.1 | 0.058564 | 0.122063 | 1.059541 | 4.07E-05 | 0.000184 |
| AC009303.1 | 0.02653 | 0.075133 | 1.501801 | 2.03E-06 | 1.35E-05 |
| KRT18P57 | 0.01516 | 0.065637 | 2.114262 | 0.005005 | 0.011838 |
| AP000593.3 | 0.072408 | 0.156008 | 1.107389 | 1.09E-07 | 1.06E-06 |
| RN7SL688P | 0.059029 | 0.123439 | 1.064312 | 0.000861 | 0.002611 |
| NAA11 | 0.015675 | 0.25435 | 4.020275 | 0.007477 | 0.016682 |
| NADK2-AS1 | 0.202463 | 0.512676 | 1.340389 | 7.69E-17 | 1.36E-14 |
| LINC01614 | 0.141103 | 0.426277 | 1.595039 | 0.001858 | 0.005058 |
| AC011939.1 | 0.049687 | 0.11112 | 1.161192 | 1.11E-06 | 7.93E-06 |
| SMG1P6 | 0.016684 | 0.034111 | 1.031752 | 1.51E-05 | 7.78E-05 |
| AC138028.1 | 0.020769 | 0.043858 | 1.078363 | 2.43E-07 | 2.11E-06 |
| AL034550.1 | 0.089667 | 0.239347 | 1.416461 | 2.01E-14 | 1.44E-12 |
| AL365277.1 | 0.15181 | 0.418282 | 1.462208 | 3.43E-17 | 6.96E-15 |
| AC012618.2 | 0.116533 | 0.248452 | 1.09223 | 1.32E-09 | 2.24E-08 |
| CCR6 | 0.018738 | 0.041575 | 1.149776 | 0.000891 | 0.002688 |
| AC009994.1 | 0.047892 | 0.22777 | 2.249713 | 1.45E-08 | 1.80E-07 |
| AC020916.2 | 0.048562 | 0.139829 | 1.525779 | 6.14E-05 | 0.000262 |
| HMGB1P14 | 0.120875 | 0.306454 | 1.342148 | 4.47E-09 | 6.42E-08 |
| AL356218.2 | 0.06881 | 0.147854 | 1.103483 | 4.27E-06 | 2.59E-05 |
| AC131235.2 | 0.015646 | 0.033282 | 1.088933 | 8.73E-06 | 4.81E-05 |
| RN7SL487P | 0.027241 | 0.098401 | 1.852881 | 5.11E-07 | 4.03E-06 |
| AC010624.4 | 0.322976 | 0.688609 | 1.092257 | 0.011725 | 0.024477 |
| C3orf22 | 0.013738 | 0.029942 | 1.124006 | 0.001095 | 0.003208 |
| HSD3BP5 | 0.182799 | 0.452997 | 1.309248 | 5.67E-09 | 7.90E-08 |
| MIR3944 | 0.082168 | 0.219556 | 1.417934 | 6.18E-05 | 0.000264 |
| AC017101.1 | 0.019632 | 0.067074 | 1.772502 | 1.31E-08 | 1.66E-07 |
| AC023206.1 | 0.046593 | 0.174687 | 1.906579 | 0.001978 | 0.005335 |
| AC091181.1 | 0.040502 | 0.280981 | 2.794391 | 3.71E-10 | 7.31E-09 |
| CSTP2 | 0.038087 | 0.082281 | 1.111258 | 0.001854 | 0.005049 |
| SPDYE2 | 0.028845 | 0.110169 | 1.933345 | 4.62E-12 | 1.59E-10 |
| AC093849.1 | 0.007139 | 0.023055 | 1.691379 | 2.71E-07 | 2.32E-06 |
| TRPV1 | 0.026346 | 0.053565 | 1.023697 | 8.02E-15 | 6.35E-13 |
| BTBD18 | 0.01854 | 0.047835 | 1.367422 | 0.00017 | 0.000638 |
| PCDHA9 | 0.013392 | 0.031165 | 1.218568 | 0.016171 | 0.032151 |
| MTCO2P27 | 0.018818 | 0.046872 | 1.316576 | 1.87E-07 | 1.68E-06 |
| C15orf54 | 0.004538 | 0.035745 | 2.977553 | 1.53E-05 | 7.88E-05 |
| MIR3657 | 0.056333 | 0.136539 | 1.277272 | 9.25E-08 | 9.17E-07 |
| AC091053.2 | 0.030412 | 0.083968 | 1.465184 | 1.91E-07 | 1.72E-06 |
| AC105046.1 | 0.035493 | 0.128281 | 1.853695 | 4.06E-05 | 0.000183 |
| AL360268.1 | 0.026105 | 0.126794 | 2.280114 | 3.35E-07 | 2.80E-06 |
| U73169.1 | 0.046333 | 0.105785 | 1.191037 | 1.12E-05 | 6.01E-05 |
| MIR3197 | 0.967039 | 2.117799 | 1.130919 | 0.000783 | 0.0024 |
| SLC25A39P1 | 0.01074 | 0.080891 | 2.912921 | 0.001552 | 0.004335 |
| KRTAP5-7 | 0.014679 | 0.029775 | 1.020312 | 1.17E-05 | 6.23E-05 |
| IGHV3-13 | 1.034342 | 3.788659 | 1.872974 | 0.009093 | 0.019735 |
| AC010480.1 | 0.097118 | 3.105804 | 4.99909 | 1.55E-09 | 2.56E-08 |
| GPR174 | 0.204688 | 0.452166 | 1.143425 | 4.75E-05 | 0.00021 |
| AC026771.1 | 0.084715 | 0.257142 | 1.601867 | 6.28E-14 | 3.89E-12 |
| DDTP1 | 0.036031 | 0.09334 | 1.373257 | 1.23E-05 | 6.48E-05 |
| IDO2 | 0.022382 | 0.200687 | 3.164534 | 0.001156 | 0.003362 |
| AP000919.1 | 0.035773 | 0.09491 | 1.407681 | 0.000105 | 0.000419 |
| IGHV3-30 | 6.345474 | 17.90065 | 1.496212 | 0.014373 | 0.029073 |
| RN7SL15P | 0.082127 | 0.246004 | 1.582749 | 1.09E-10 | 2.50E-09 |
| SNORD62B | 1.100164 | 2.697496 | 1.293902 | 1.62E-18 | 5.46E-16 |
| MIR378H | 0.186125 | 0.387795 | 1.059025 | 2.99E-05 | 0.00014 |
| MIR5685 | 0.692447 | 1.500438 | 1.115609 | 8.85E-14 | 5.20E-12 |
| SNORD116-27 | 0.226462 | 0.666799 | 1.557984 | 0.003523 | 0.008771 |
| AC245884.10 | 0.101992 | 0.274055 | 1.426004 | 0.005298 | 0.012418 |
| AC020910.1 | 0.031845 | 0.095035 | 1.577416 | 0.00025 | 0.00089 |
| RN7SL648P | 0.055343 | 0.825163 | 3.898199 | 8.94E-05 | 0.000365 |
| IL6 | 1.588439 | 3.722407 | 1.228626 | 0.003548 | 0.008821 |
| HTR1F | 0.040855 | 0.083222 | 1.026454 | 0.00167 | 0.004613 |
| AC007673.1 | 0.026887 | 0.099817 | 1.892379 | 7.28E-09 | 9.85E-08 |
| SNORA22B | 0.091589 | 0.239505 | 1.38681 | 6.55E-07 | 5.01E-06 |
| AC005757.1 | 0.018956 | 0.04698 | 1.309431 | 0.000225 | 0.000811 |
| RN7SKP74 | 0.068418 | 0.1906 | 1.478104 | 5.43E-09 | 7.60E-08 |
| PRAME | 0.335025 | 0.825972 | 1.301826 | 0.001067 | 0.003136 |
| AC015971.1 | 0.086801 | 0.276347 | 1.670695 | 1.61E-20 | 1.43E-17 |
| AC002401.2 | 0.026487 | 0.053523 | 1.014897 | 0.016704 | 0.033028 |
| AC010271.1 | 0.271258 | 0.701591 | 1.370963 | 6.45E-19 | 2.47E-16 |
| SGK2 | 0.273854 | 0.558208 | 1.027398 | 6.89E-06 | 3.92E-05 |
| AC104564.5 | 0.038283 | 0.101303 | 1.403904 | 0.000198 | 0.000729 |
| AC145285.3 | 0.105543 | 0.221425 | 1.068986 | 3.66E-07 | 3.02E-06 |
| ARAP1-AS2 | 0.053035 | 0.147207 | 1.472816 | 4.83E-13 | 2.26E-11 |
| AC010168.1 | 0.141709 | 0.332777 | 1.231625 | 0.006575 | 0.014933 |
| OR1H1P | 0.026311 | 0.057123 | 1.118372 | 3.15E-05 | 0.000147 |
| AC003681.1 | 0.067741 | 0.154353 | 1.188131 | 1.32E-19 | 6.49E-17 |
| AC016597.1 | 0.028848 | 0.062164 | 1.107603 | 1.31E-05 | 6.84E-05 |
| AC068491.4 | 0.006391 | 0.014435 | 1.175394 | 0.025716 | 0.047473 |
| FLJ42393 | 0.030361 | 0.316451 | 3.381713 | 5.19E-06 | 3.07E-05 |
| AC127024.8 | 0.057355 | 0.174076 | 1.601727 | 3.21E-15 | 2.96E-13 |
| Z95331.1 | 0.030621 | 0.070509 | 1.203298 | 3.93E-06 | 2.41E-05 |
| AC019072.1 | 0.00873 | 0.093526 | 3.421351 | 4.02E-05 | 0.000182 |
| ADAM12 | 0.124633 | 0.267021 | 1.099273 | 0.000192 | 0.000711 |
| RPS12P17 | 0.033655 | 0.128761 | 1.93581 | 0.025278 | 0.046809 |
| RUVBL1-AS1 | 0.02301 | 0.064286 | 1.482251 | 3.25E-06 | 2.04E-05 |
| OR52V1P | 0.012231 | 0.031001 | 1.341731 | 3.53E-05 | 0.000163 |
| MIR4512 | 0.178033 | 0.375366 | 1.076148 | 7.42E-08 | 7.57E-07 |
| AC022336.1 | 0.037533 | 0.088738 | 1.241413 | 5.08E-09 | 7.19E-08 |
| LINC00954 | 0.070293 | 0.152623 | 1.118526 | 2.50E-05 | 0.00012 |
| AC009093.1 | 0.039866 | 0.201443 | 2.337126 | 2.15E-06 | 1.43E-05 |
| AC104109.4 | 0.054979 | 0.128314 | 1.222718 | 1.74E-21 | 2.82E-18 |
| TAS2R30 | 0.011944 | 0.089873 | 2.911613 | 0.002833 | 0.007269 |
| AP001189.1 | 0.198394 | 0.407067 | 1.036898 | 1.53E-12 | 6.12E-11 |
| OR7E117P | 0.022227 | 0.049538 | 1.15621 | 8.66E-06 | 4.78E-05 |
| ATG12P2 | 0.102573 | 0.528593 | 2.365505 | 1.66E-06 | 1.13E-05 |
| AC111170.3 | 0.062906 | 0.153855 | 1.290311 | 0.00152 | 0.004256 |
| AC023830.1 | 0.022302 | 0.045125 | 1.016743 | 4.80E-05 | 0.000212 |
| MYCBP2-AS2 | 0.033064 | 0.105561 | 1.674743 | 2.72E-05 | 0.000129 |
| MRTFA-AS1 | 0.019042 | 0.095452 | 2.325569 | 0.026339 | 0.048406 |
| C1orf147 | 0.024784 | 0.070916 | 1.516702 | 4.09E-09 | 5.93E-08 |
| AC073367.1 | 0.099011 | 0.221965 | 1.164671 | 7.91E-10 | 1.43E-08 |
| LY6H | 0.592292 | 0.175593 | -1.75407 | 1.92E-06 | 1.29E-05 |
| AL591806.1 | 0.008587 | 0.032677 | 1.928062 | 0.000109 | 0.000435 |
| AL353807.4 | 0.427897 | 1.035043 | 1.274354 | 1.29E-14 | 9.65E-13 |
| RNA5SP494 | 0.157962 | 0.342514 | 1.11658 | 0.000236 | 0.000846 |
| AC007952.4 | 0.065514 | 0.179448 | 1.453685 | 0.000359 | 0.001219 |
| AL353593.3 | 0.094789 | 0.36764 | 1.955503 | 1.55E-08 | 1.92E-07 |
| FNDC9 | 0.00878 | 0.032249 | 1.876987 | 3.06E-06 | 1.94E-05 |
| HSPE1P11 | 0.062009 | 0.246254 | 1.989585 | 0.019696 | 0.037925 |
| AC239798.2 | 0.037407 | 0.085525 | 1.193041 | 0.00079 | 0.002419 |
| AC099811.4 | 0.041686 | 0.152632 | 1.872412 | 2.63E-05 | 0.000126 |
| VDAC1P11 | 0.012428 | 0.057804 | 2.217619 | 7.25E-11 | 1.75E-09 |
| MTCYBP35 | 0.041415 | 0.085415 | 1.044339 | 0.009913 | 0.021252 |
| SNORA71B | 0.543129 | 1.173543 | 1.111504 | 1.14E-13 | 6.42E-12 |
| RPL13P4 | 0.015169 | 0.070783 | 2.222236 | 9.10E-08 | 9.05E-07 |
| AC087286.2 | 0.091148 | 0.438637 | 2.266746 | 1.31E-07 | 1.24E-06 |
| AC108463.3 | 0.014683 | 0.037352 | 1.346995 | 1.29E-05 | 6.78E-05 |
| AL162274.1 | 0.096959 | 0.241066 | 1.313983 | 5.52E-17 | 1.04E-14 |
| AP000688.2 | 0.056596 | 0.213941 | 1.918442 | 0.002807 | 0.007208 |
| AC110792.2 | 0.008318 | 0.043482 | 2.386043 | 0.000213 | 0.000775 |
| AC067852.3 | 0.154846 | 0.390982 | 1.336266 | 2.89E-18 | 8.75E-16 |
| Z98885.1 | 0.038778 | 0.200895 | 2.373131 | 0.000929 | 0.002786 |
| RN7SKP299 | 0.037411 | 0.333881 | 3.157791 | 0.00159 | 0.004424 |
| ZBTB20-AS5 | 0.006482 | 0.814132 | 6.972744 | 0.002496 | 0.006503 |
| CEACAM22P | 0.615353 | 1.560659 | 1.342669 | 0.003309 | 0.008314 |
| CIDEC | 0.131424 | 0.063912 | -1.04008 | 0.000166 | 0.000626 |
| AL138828.1 | 0.004137 | 0.028357 | 2.776956 | 0.007264 | 0.016276 |
| AL031281.1 | 0.031681 | 0.151744 | 2.259934 | 0.000544 | 0.001752 |
| IGHV1-12 | 0.228475 | 0.645587 | 1.498574 | 0.001514 | 0.004242 |
| MYLK-AS2 | 0.015972 | 0.056021 | 1.810437 | 0.002858 | 0.007322 |
| AL445933.2 | 0.048105 | 0.175075 | 1.863701 | 5.34E-05 | 0.000232 |
| EREG | 0.021602 | 0.048184 | 1.157363 | 1.10E-05 | 5.89E-05 |
| BACH1-IT2 | 0.109081 | 0.224356 | 1.040386 | 0.000696 | 0.00217 |
| IGHV3-21 | 5.480012 | 11.37594 | 1.053735 | 0.009366 | 0.020232 |
| AC108010.1 | 0.830895 | 1.939408 | 1.222878 | 9.46E-18 | 2.39E-15 |
| KCND3-AS1 | 0.01737 | 0.182467 | 3.392954 | 0.000236 | 0.000848 |
| TAF9BP1 | 0.016386 | 0.158795 | 3.276619 | 5.35E-08 | 5.68E-07 |
| AC022146.2 | 0.240683 | 0.550997 | 1.19491 | 3.42E-06 | 2.14E-05 |
| ETF1P2 | 0.020498 | 0.154023 | 2.909569 | 2.81E-09 | 4.28E-08 |
| AC055811.1 | 0.021439 | 0.060558 | 1.498072 | 4.20E-07 | 3.40E-06 |
| ARGFXP2 | 0.032798 | 0.110965 | 1.758441 | 4.67E-09 | 6.67E-08 |
| NPM1P29 | 0.035307 | 0.108988 | 1.626153 | 3.04E-05 | 0.000143 |
| AL132656.3 | 0.009162 | 0.028457 | 1.635091 | 7.53E-06 | 4.23E-05 |
| HNRNPA1P14 | 0.09165 | 0.498416 | 2.443148 | 2.38E-13 | 1.21E-11 |
| AP003108.4 | 0.027517 | 0.087122 | 1.662726 | 3.10E-11 | 8.29E-10 |
| MIR5704 | 0.713503 | 2.268118 | 1.668504 | 4.50E-10 | 8.65E-09 |
| AC034102.8 | 0.149739 | 0.302747 | 1.015658 | 4.17E-07 | 3.38E-06 |
| AC234778.2 | 0.031223 | 0.067408 | 1.110293 | 9.20E-05 | 0.000374 |
| IFIT3 | 3.950812 | 8.389843 | 1.086495 | 0.000525 | 0.001699 |
| AL162385.2 | 0.096854 | 0.224788 | 1.21468 | 1.12E-07 | 1.08E-06 |
| RNU1-108P | 0.082997 | 0.347619 | 2.066373 | 0.01595 | 0.03181 |
| AC015871.4 | 0.010095 | 0.038818 | 1.943145 | 0.000839 | 0.002552 |
| AC021739.4 | 0.022644 | 0.063296 | 1.482996 | 5.23E-07 | 4.11E-06 |
| AL035404.2 | 0.03384 | 0.101761 | 1.588385 | 3.02E-05 | 0.000142 |
| RNU6-1053P | 0.409129 | 1.159266 | 1.502583 | 7.36E-14 | 4.45E-12 |
| FAM205A | 0.007424 | 0.018147 | 1.28935 | 1.41E-08 | 1.76E-07 |
| MICD | 0.039559 | 0.092163 | 1.22019 | 2.31E-06 | 1.51E-05 |
| ASPG | 0.093369 | 0.196688 | 1.0749 | 6.80E-17 | 1.23E-14 |
| AC005261.5 | 0.479209 | 1.051297 | 1.133445 | 0.000313 | 0.001083 |
| AC087632.1 | 0.009204 | 0.021116 | 1.19793 | 2.28E-06 | 1.50E-05 |
| HDAC1P2 | 0.014779 | 0.032202 | 1.123574 | 8.55E-06 | 4.73E-05 |
| PFN1P3 | 0.078745 | 0.16778 | 1.091314 | 7.47E-07 | 5.62E-06 |
| AC090617.4 | 0.027395 | 0.095695 | 1.804556 | 5.99E-06 | 3.48E-05 |
| AC114737.1 | 0.017056 | 0.080136 | 2.232172 | 6.71E-06 | 3.83E-05 |
| AL445223.1 | 0.008687 | 0.148544 | 4.095825 | 6.26E-05 | 0.000267 |
| AL513523.1 | 0.028135 | 0.071191 | 1.339309 | 8.05E-08 | 8.13E-07 |
| SLFN12L | 0.085209 | 0.18526 | 1.120482 | 2.88E-06 | 1.84E-05 |
| MIR548AA1 | 0.138627 | 0.289461 | 1.062166 | 3.95E-06 | 2.42E-05 |
| AC093690.1 | 0.060543 | 0.137652 | 1.185001 | 1.09E-08 | 1.41E-07 |
| RNU4-89P | 0.169684 | 0.437888 | 1.367713 | 3.98E-07 | 3.24E-06 |
| RASA3-IT1 | 0.030953 | 0.083596 | 1.433351 | 0.004744 | 0.011312 |
| AL590762.1 | 0.140534 | 0.463667 | 1.722173 | 9.18E-10 | 1.62E-08 |
| RN7SKP243 | 0.023427 | 0.144557 | 2.625383 | 1.34E-09 | 2.27E-08 |
| AC022272.1 | 0.056862 | 0.191582 | 1.75243 | 1.38E-12 | 5.63E-11 |
| AC007919.1 | 0.297148 | 0.915126 | 1.622787 | 7.82E-11 | 1.87E-09 |
| AC098617.1 | 0.077714 | 0.684057 | 3.13787 | 0.001254 | 0.003609 |
| CCDC194 | 0.024934 | 0.146299 | 2.552704 | 1.54E-07 | 1.42E-06 |
| HNRNPA1P36 | 0.066998 | 0.16014 | 1.257139 | 7.91E-06 | 4.41E-05 |
| LINC01210 | 0.068425 | 0.165771 | 1.276602 | 0.02211 | 0.041814 |
| LINC01450 | 0.002304 | 0.006288 | 1.448777 | 7.69E-06 | 4.31E-05 |
| SNX18P9 | 0.019099 | 0.038586 | 1.014552 | 0.023711 | 0.044375 |
| Z99289.1 | 0.025322 | 0.127329 | 2.3301 | 1.43E-08 | 1.78E-07 |
| AC104619.1 | 0.467906 | 0.198668 | -1.23586 | 2.09E-07 | 1.86E-06 |
| CNTFR-AS1 | 0.008561 | 0.017291 | 1.014212 | 0.001319 | 0.003771 |
| NEAT1 | 38.48054 | 78.09586 | 1.021117 | 5.98E-13 | 2.72E-11 |
| AC087521.2 | 0.035241 | 0.086515 | 1.29568 | 1.24E-13 | 6.91E-12 |
| KCTD19 | 0.009029 | 0.027876 | 1.626416 | 0.000114 | 0.000453 |
| KCNT1 | 0.020811 | 0.046806 | 1.16933 | 1.05E-12 | 4.38E-11 |
| AC004775.1 | 0.071968 | 0.15311 | 1.089134 | 0.000196 | 0.000722 |
| AC015911.8 | 0.108861 | 0.316883 | 1.541463 | 6.89E-10 | 1.26E-08 |
| Z98742.3 | 0.032995 | 0.077829 | 1.238064 | 0.001337 | 0.003817 |
| PCBP2P1 | 0.016882 | 0.039652 | 1.231948 | 8.36E-07 | 6.20E-06 |
| SLC34A3 | 0.215242 | 0.450376 | 1.065168 | 2.55E-09 | 3.92E-08 |
| ROS1 | 0.022679 | 0.046096 | 1.023319 | 3.06E-06 | 1.94E-05 |
| AC074135.1 | 0.20867 | 0.505027 | 1.275139 | 1.64E-05 | 8.32E-05 |
| HNRNPH1P1 | 0.105345 | 0.303907 | 1.528512 | 1.57E-11 | 4.62E-10 |
| ERICH4 | 0.027233 | 0.061516 | 1.175582 | 0.000552 | 0.001777 |
| AC018529.3 | 0.024261 | 0.162322 | 2.742144 | 1.84E-08 | 2.24E-07 |
| RPL21P136 | 0.046852 | 0.094129 | 1.00652 | 0.01435 | 0.029038 |
| MAPK6P1 | 0.009612 | 0.049947 | 2.377482 | 1.45E-09 | 2.42E-08 |
| AC093525.8 | 0.353901 | 0.85087 | 1.265594 | 1.77E-22 | 6.96E-19 |
| AL121772.2 | 0.033617 | 0.071579 | 1.090348 | 1.87E-05 | 9.35E-05 |
| AC015908.1 | 0.033472 | 0.104239 | 1.638867 | 4.33E-05 | 0.000194 |
| RNY1P4 | 0.087215 | 0.490429 | 2.491395 | 4.29E-07 | 3.45E-06 |
| AC078778.2 | 0.063434 | 0.157486 | 1.311899 | 2.61E-10 | 5.38E-09 |
| AL158151.2 | 0.142637 | 0.297421 | 1.060152 | 1.89E-07 | 1.70E-06 |
| AC009229.2 | 0.199921 | 0.433113 | 1.115316 | 0.001336 | 0.003814 |
| RNU6-1016P | 1.046274 | 4.981832 | 2.251416 | 6.17E-15 | 5.12E-13 |
| AC073592.1 | 0.140095 | 0.281012 | 1.004226 | 1.57E-06 | 1.08E-05 |
| RNU2-69P | 0.162941 | 0.500067 | 1.61777 | 0.000187 | 0.000695 |
| RN7SL481P | 0.12693 | 0.263454 | 1.05352 | 4.43E-08 | 4.83E-07 |
| TSEN15P2 | 0.030268 | 0.166869 | 2.462847 | 0.001152 | 0.003353 |
| SMG1P1 | 0.035414 | 0.073214 | 1.047815 | 1.54E-09 | 2.55E-08 |
| CASK-AS1 | 0.040389 | 0.082424 | 1.029096 | 4.35E-08 | 4.74E-07 |
| AP001124.1 | 0.042094 | 0.10969 | 1.381737 | 0.015497 | 0.031037 |
| AC016245.1 | 0.003937 | 0.010619 | 1.431565 | 1.79E-05 | 8.99E-05 |
| AK3P5 | 0.021677 | 0.451721 | 4.381172 | 9.22E-06 | 5.06E-05 |
| RNU6-844P | 0.335576 | 0.847587 | 1.336721 | 2.34E-07 | 2.05E-06 |
| RDH10-AS1 | 0.077466 | 0.295364 | 1.930868 | 1.94E-08 | 2.34E-07 |
| AC016747.3 | 0.117718 | 0.349162 | 1.568555 | 2.85E-12 | 1.05E-10 |
| C9orf41-AS1 | 0.074795 | 0.192528 | 1.364055 | 1.84E-09 | 2.97E-08 |
